# OLICH: A reference library of DNA barcodes for Nordic lichens

**DOI:** 10.3897/BDJ.7.e36252

**Published:** 2019-08-19

**Authors:** Gunnhild Marthinsen, Siri Rui, Einar Timdal

**Affiliations:** 1 Natural History Museum, University of Oslo, Oslo, Norway Natural History Museum, University of Oslo Oslo Norway

**Keywords:** DNA barcoding, ITS, Lichenised fungi, Norway, Taxonomy

## Abstract

**Background:**

DNA barcodes are increasingly being used for species identification amongst the lichenised fungi. This paper presents a dataset aiming to provide an authoritative DNA barcode sequence library for a wide array of Nordic lichens.

**New information:**

We present 1324 DNA barcode sequences (nrITS) for 507 species in 175 genera and 25 orders. Thirty-eight species are new to GenBank and, for 25 additional species, ITS sequences are here presented for the first time. The dataset covers 20–21% of the Nordic lichenised species. Barcode gap analyses are given and discussed for the three genera *Cladonia*, *Ramalina* and *Umbilicaria*. The new combination *Bryobilimbia fissuriseda* (Poelt) Timdal, Marthinsen & Rui is proposed for *Mycobilimbia fissuriseda* and Nordic material of the species, currently referred to as *Pseudocyphellaria crocata* and Psoroma tenue ssp. boreale, are shown to belong in *Pseudocyphellaria citrina* and *Psoroma cinnamomeum*, respectively.

## Introduction

Lichens are fungi living in mutualistic symbiosis with green algae and/or cyanobacteria. They are highly polyphyletic and 20–30 independent lichenisation events may have occurred during the evolution of higher Fungi (Schoch et al. 2009, Lücking et al. 2017a). The lichenised fungi currently consists of more than 19,000 species, i.e. about 17% of all known fungal species and 27% of all known ascomycete species (Lücking et al. 2017a). They occur globally in all major terrestrial ecosystems, especially on trees, soil and rock. Although a lichen thallus consists of a symbiotic association between a fungal exhabitant, the mycobiont and one or more photosynthetic algal and/or cyanobacterial inhabitants, the photobionts, the taxonomy and nomenclature follow that of the mycobiont. A lichen thallus may house additional fungi, i.e. parasites, parasymbionts and endophytes (Hawksworth 1982, Richardson 1999, Spribille et al. 2016), making a lichen thallus a complex symbiotic assemblage.

Identifying lichenised fungi has traditionally been based on morphological, anatomical and chemical characters, but DNA-based identification is now increasingly being used. In 2012, the internal transcribed spacer of nuclear ribosomal DNA (ITS) was recommended as the primary fungal DNA barcode marker by the International Fungal Barcoding Consortium (Xu 2016), following studies on its suitability by for example Schoch et al. (2012). For lichenised fungi, studies reporting successful use of the ITS marker for species delimitations include Kelly et al. (2011), Zheng et al. (2011), Parnmen et al. (2013), Leavitt et al. (2014), Magain and Serusiaux (2015) and Divakar et al. (2016). Pino-Bodas et al. (2013), however, found ITS often failing for species delimitations in *Cladonia* and investigated alternative markers.

In this paper, we present a DNA barcode dataset for Nordic lichens, including 1324 records, comprising 507 species in 175 genera and 25 different orders.

## General description

### Purpose

This dataset aims to provide an authoritative DNA barcode sequences library for a wide array of Nordic lichens. The library should facilitate DNA-based identification of species for both traditional molecular studies and DNA-metabarcoding studies and should also be a resource for biosystematic research on Nordic lichens.

### Additional information

Eighty sequences have been published previously, but are included here as they were produced under the OLICH project, i.e. GenBank IDs KT800002–07 (Westberg et al. 2015), KU687445–60 (McCune et al. 2016), KU873926–32 (Timdal et al. 2016), KY682961 (Pykälä et al. 2018), 38 sequences within the series MG838156–201 (Kistenich et al. 2018), MG968256–61 (Haugan and Timdal 2018) and MK567159–64 (Haugan and Timdal 2019). In addition, 151 sequences (KY266831–981) have been submitted to GenBank previously for an unpublished project.

**Results**: Thirty-eight species are new to GenBank: *Arthonia phaeobaea*, *Arthopyrenia salicis*, *Bactrospora corticola*, *Baeomyces carneus*, *Buellia arnoldii*, *B. dives*, *Candelariella arctica*, *Cladonia cyanipes*, *Coccotrema citrinescens*, *Eopyrenula leucoplaca*, *Epilichen glauconigellus*, *E. scabrosus*, *Gyalecta foveolaris*, *Lecanora atrosulphurea*, *L. leptacina*, *Ochrolechia alaskana*, *O. alboflavescens*, *O. gowardii*, *O. mahluensis*, *Orphniospora moriopsis*, *Phaeophyscia kairamoi*, *Pilophorus dovrensis*, *Pseudocyphellaria norvegica*, *Pyrenula occidentalis*, *Ramalina cuspidata*, *Rhizocarpon badioatrum*, *R. bolanderi*, *R. chioneum*, *R. jemtlandicum*, *R. leptolepis*, *R. rittokense*, *R. santessonii*, *R. subgeminatum*, *Rinodina balanina*, *R. disjuncta*, *R. roboris*, *Stereocaulon capitellatum* and *S. symphycheilum*.

Twenty-five additional species are previously represented in GenBank, but not with ITS sequences: *Arthonia didyma*, *Bryobilimbia fissuriseda*, *Buellia epigaea*, *Callome multipartita*, *Dendrographa latebrarum*, *Fuscidea mollis*, *Gyalecta flotowii*, *G. friesii*, *G. geoica*, *Gyrographa gyrocarpa*, *Halecania alpivaga*, *Koerberiella wimmeriana*, *Opegrapha vermicellifera*, *Pertusaria aspergilla*, *P. carneopallida*, *Phlyctis agelaea*, *Placynthiella oligotropha*, *Psoronactis dilleniana*, *Pyrenula laevigata*, *Ramboldia elabens*, *Sagiolechia protuberans*, *Stereocaulon grande*, *Thelotrema macrosporum*, *Tremolecia atrata* and *Varicellaria lactea*.

## Project description

### Title

The name OLICH refers to “contributions from the **O**slo **lich**en herbarium”.

### Personnel

Gunnhild Marthinsen (project coordinator), Siri Rui (project engineer), Einar Timdal (taxonomic supervisor), all affiliated to the Natural History Museum, University of Oslo.

### Study area description

**Figure 1.**
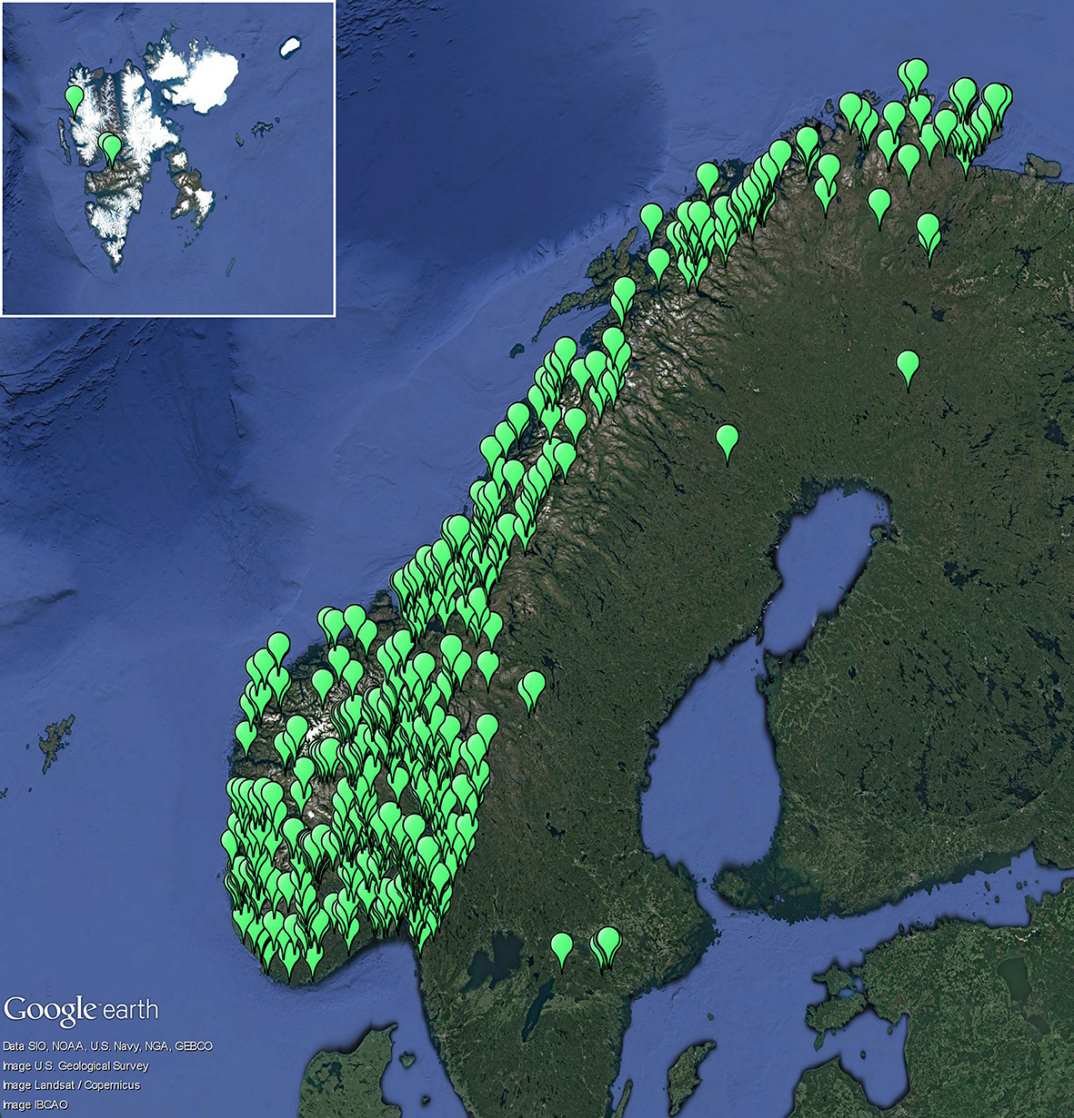
Distribution of the OLICH records

The Nordic countries, including Svalbard: Norway, mainland (97.0% of the specimens), Sweden (2.0%), Finland (0.5%), Norway, Svalbard (0.5%), Fig. 1.

### Funding

This project is funded by the Norwegian Research Council (RCN 226134) through the Norwegian Barcode of Life project (NorBOL). In addition, the Norwegian Species Information Centre (Artsdatabanken) has funded four projects on lichens under the Norwegian Taxonomy Initiative from which material has been collected and/or sampled (project numbers 70184216, 70184227, 70184233, and 70184237).

## Sampling methods

### Sampling description

**Figure 2.**
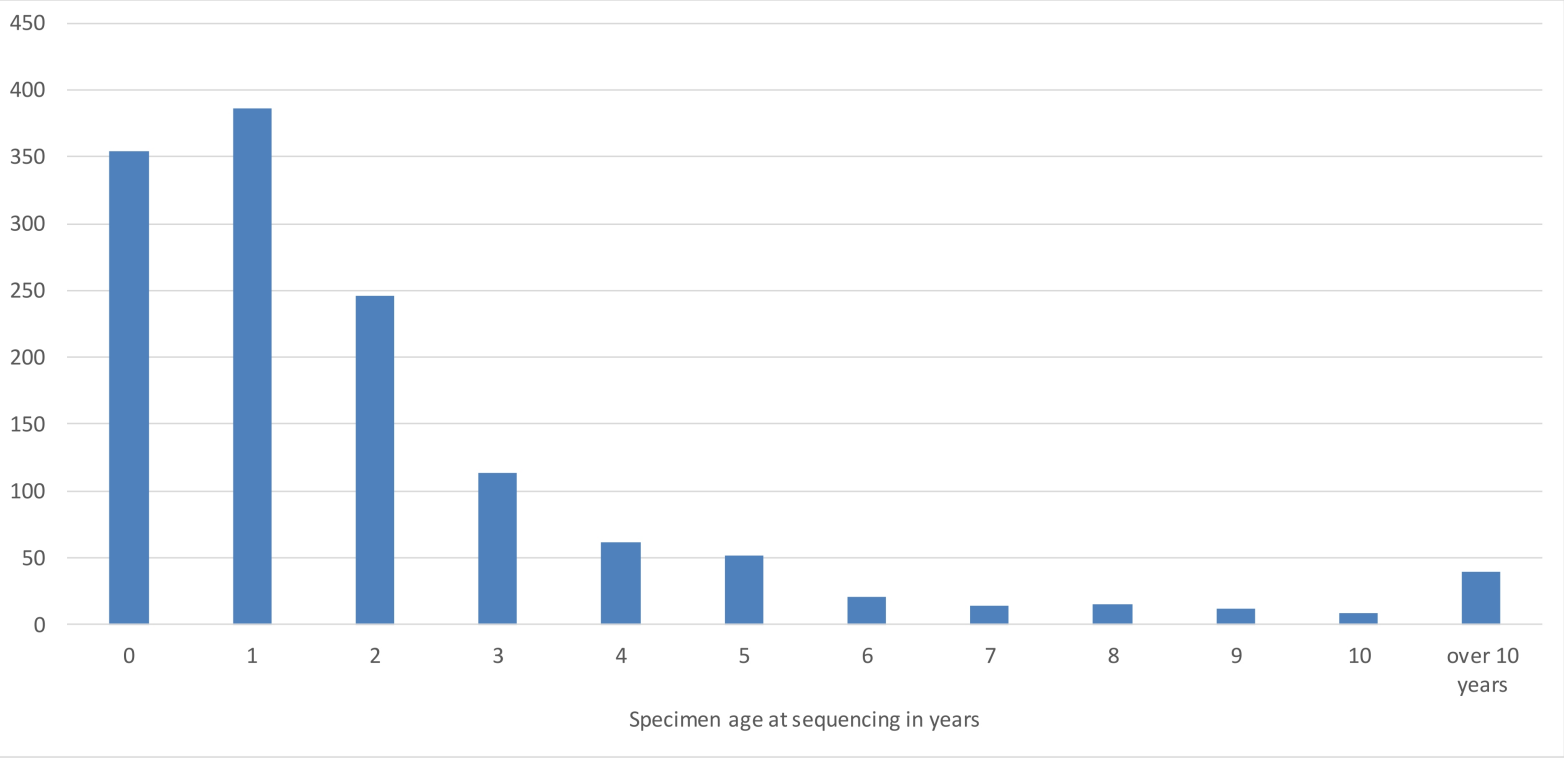
Distribution of specimen age (years after collecting) at sequencing in the OLICH dataset.

The material was partly freshly collected specimens from the field and partly previously curated specimens in university herbaria. The distribution of specimen age at seqencing is given in Fig. 2. The specimens were initially determined by experts based on morphology and secondary chemistry and the identifications were re-examined at the quality control after sequence acquisition. All collected material were, after sampling, deposited in university herbaria and the entire material is housed in BG (2.1%), H (0.1%), O (94.9%), SAV (0.2%), TRH (1.7%) and TROM (1.1%) (abbreviated according to *Index Herbariorum*; http://sweetgum.nybg.org/science/ih).

Thin-layer chromatography (TLC) was performed for identification of secondary compounds on selected specimens in accordance with the methods of Culberson (1972), as modified by Menlove (1974) and Culberson and Johnson (1982). Solvent system B' was used for routine examination, A and C for additional confirmation.

Tissue of thallus or preferably apothecia (about 3 mm^3^) was assembled in 96 well plates obtained from the Canadian Centre for DNA Barcoding (CCDB; http://ccdb.ca). One well was left empty to serve as negative control. The freshly collected material was either sampled in the field or at a field station. In the field, the specimen was first photographed, then tissue was removed with a flame-decontaminated forceps and placed in the well and finally the specimen was collected. Specimens requiring more controlled handling were photographed and sampled either with forceps or scalpel under a binocular lens at a field station. Wells containing fresh material were dried for several days with lids open in a drying chamber containing silica gel. Herbarium material was sampled at the housing institutions under binocular lenses.

Field data was stored on tablets and GPSs. Tissue sampling data and photography data were transferred directly to standard BOLD spreadsheets, whereas the collection data passed through herbarium databases for obtaining institution storing, voucher ID (= sample ID), uniform nomenclature and taxonomical hierarchy and contact information. Material sampled in herbaria used a similar pipeline, although simpler, as collection data was already available.

The plates were sent to CCDB for DNA extraction (following Ivanova et al. 2011) and sequencing of the ITS region (following Kuzmina and Ivanova 2011). Primers used were ITS1-F or ITS5 (forward) and ITS4 (reverse) (Gardes and Bruns 1993, White et al. 1990). Trace files and sequences were uploaded to BOLD (Ratnasingham and Hebert 2007). The sequences were edited at CCDB, but occasionally inspected and re-edited at OLICH.

### Quality control

All OLICH sequences were subjected to a local BLASTn search against a database consisting of all lichen ITS sequences in GenBank (downloaded 26-06-2018) and all unpublished lichen ITS sequences were produced at various projects at our own institution, including the OLICH dataset. We inspected automatically generated distance-based neighbour-joining trees for all genera represented in the OLICH dataset, i.e. we merged all OLICH sequences with all GenBank ITS sequences from the same genus before running ClustalW2 (http://www.clustal.org/clustal2) in batch mode (one run for each genus) and saving the trees. For selected cases (Figs. 5–15), phylogenetic analyses were performed through the following pipeline: selecting relevant additional sequences for comparison from the GenBank download; aligning the dataset using the online version of MAFFT (https://mafft.cbrc.jp/alignment/server); trimming ends and manually editing the alignment using BioEdit 7.1.3.0 (Hall 1999); analysing the alignment by the maximum likelihood method using RAxML v. 8.2.10 (Stamatakis 2014) by applying the “ML + rapid bootstrap” method with 100 bootstrap repeats and the GTRGAMMA substitution model; and editing the best-scoring tree using TreeGraph2 (Stöver and Müller 2010).

To further check that the sequences and species determinations were correct and also to check whether species delimitation, based on traditional characters, coincides with ITS species delimitation, we performed barcode gap analyses. The three genera in our dataset with most sampled species were chosen for these analyses; *Cladonia*, *Ramalina* and *Umbilicaria*. Sequences were aligned separately for each genus using the ClustalW algorithm implemented in MEGA7 (Kumar et al. 2016), with additional manual editing. Pairwise genetic distances between all specimens within each of the three genera were calculated in MEGA7, using the K2P substitution model. The distances were summarised in a bar plot, based on a histogram of all pairwise distances. For each species pair within the genera, we plotted the maximum intraspecific distance against the minimum distance to nearest neighbour-species.

## Geographic coverage

### Description

The Nordic countries, including Svalbard (Fig. 1).

### Coordinates

58.02°N and 78.94°N Latitude; 5.06°E and 31.08°E Longitude.

## Taxonomic coverage

### Description

The OLICH dataset comprises 1324 records of 507 species belonging to 25 orders, 8 classes and 2 phyla of fungi (Table 1). The Nordic lichen flora comprises some 2400–2500 lichenised species (2387 are listed in Santesson et al. (2004)), i.e. the dataset comprises about 20–21% of the currently known Nordic species. Several taxonomic groups are still poorly understood, however, making the production of authoritative DNA-barcodes for many species premature due to uncertain taxonomy and/or lack of fresh material. The dataset is hence biased towards the more well-known groups, mainly the macrolichens in the orders Lecanorales (233 spp.), Peltigerales (62 spp.) and Caliciales (37 spp.) of the class Lecanoromycetes (Ascomycota). The taxonomy and nomenclature follows Nordin et al. (2019) unless commented on under Observations below.

## Temporal coverage

### Notes

**Figure 3.**
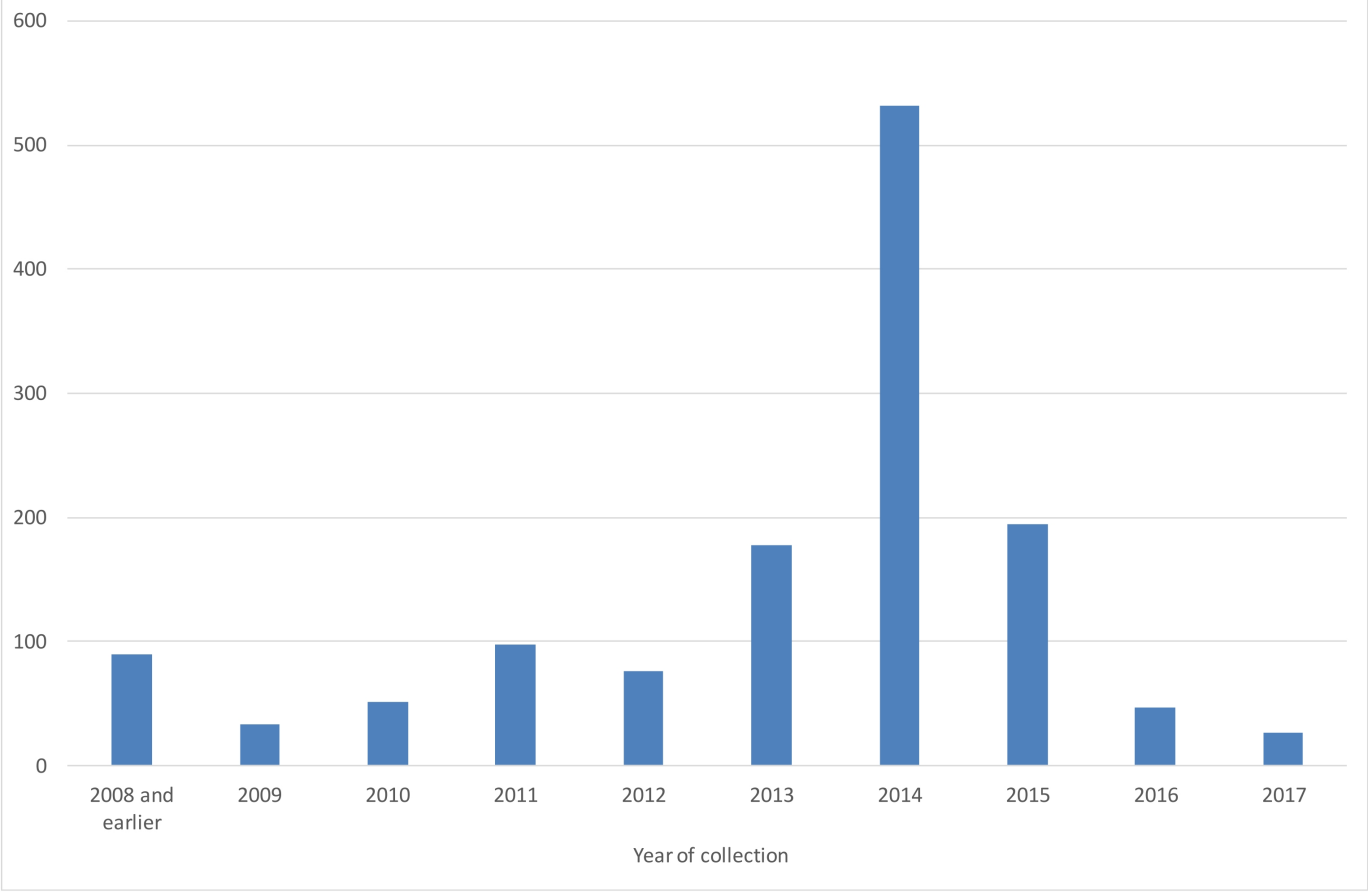
Distribution of year of collection of the specimens in the OLICH dataset

The sampled material was collected in the period 1995–2017, with 90% being after 2010 and with a peak of 532 specimens in 2014 (Fig. 3).

## Usage rights

### Use license

Open Data Commons Attribution License

## Data resources

### Data package title

DNA barcodes of Nordic lichens

### Resource link

dx.doi.org/10.5883/DS-OLICH

### Number of data sets

1

### Data set 1.

#### Data set name

DS-OLICH

#### Data format

dwc, xml, tsv, fasta, trace

#### Number of columns

41

#### Download URL

http://www.boldsystems.org/index.php/Public_SearchTerms?query=DS-OLICH

#### Description

The OLICH dataset can be downloaded from the Public Data Portal of BOLD (www.boldsystems.org) in different formats (data as dwc, xml or tsv, sequences as fasta and trace files as trace). Alternatively, BOLD users can log in and access the dataset via the Workbench platform of BOLD. All records are also searchable within BOLD using the search function of the database.

Depending on future funding, the OLICH project will continue sequencing Nordic lichens for the BOLD database, with the ultimate (although unrealistic) goal of complete coverage. Hence, the dataset will be updated in the future with new sequences and information. The version of the dataset at the time of writing the manuscript is included as Suppl. materials 1, 2, 3, 4 in the form of two text files for record information as downloaded from BOLD, one text file with the collecting and identification data in Darwin Core Standard format (downloaded from BOLD and formatted by the authors) and of a fasta file containing all sequences as downloaded from BOLD.

It should be noted that, as the BOLD database is not compliant with the Darwin Core Standard format, the Darwin Core formatted file (dwc) that can be downloaded from BOLD is not strictly Darwin Core formatted. For a proper Darwin Core formatted file, see Suppl. material 3.

All records have images and geographical coordinates in BOLD.

Column labels below follow the labels downloaded in the tsv format. Columns with no content in our dataset are left out in the list below.

**Data set 1.** 

Column label	Column description
processid	Unique identifier for the sample
sampleid	Unique identifier for the voucher specimen, i.e. museum catalogue number in either lichen herbarium or DNA bank at the Natural History Museum, University of Oslo
recordID	Entry number in the database
catalognum	Same as sampleid; Unique identifier for the voucher specimen, i.e. museum catalogue number in either lichen herbarium or DNA bank at the Natural History Museum, University of Oslo
institution_storing	The full name of the institution where specimen is stored
phylum_taxID	Taxonomic identifier of level Phylum
phylum_name	Phylum name
class_taxID	Taxonomic identifier of level Class
class_name	Class name
order_taxID	Taxonomic identifier of level Order
order_name	Order name
family_taxID	Taxonomic identifier of level Family
family_name	Family name
genus_taxID	Taxonomic identifier of level Genus
genus_name	Genus name
species_taxID	Taxonomic identifier of level Species
species_name	Species name
identification_provided_by	Name of primary individual repsonsible for providing taxonomic identification of the specimen
identification_method	The method(s) used to identify the specimen
identification_reference	Authorship of species name
voucher_status	Status of the specimen in an accessioning process
tissue_type	A brief description of the type of tissue analysed
collectors	Name(s) of the individual(s) responsible for collecting the sample in the field
habitat	A description of the habitat
Notes	Notes regarding species identification of the specimen or other note concerning the specimen.
lat	The geographical latitude (in decimal degrees) of the geographic centre of a location
long	The geographical longitude (in decimal degrees) of the geographic centre of a location
coord_accuracy	An estimation of the uncertainty of the coordinates given in the lat and long columns (metres)
elev	Elevation of sampling site. Measured in metres relative to sea level.
country	Name of country in which the organism was collected
province_state	Name of the province (in Norway "fylke") in which the organism was collected
region	Name of municipality in which the organism was collected
exactsite	Name of exact locality where the organism was collected
image_ids	Entry number in the database
image_urls	URL(s) from which the image(s) of the organism(s) can be accessed
media_descriptors	Term depicting, in this case, side of organism photographed, i.e. dorsal
captions	Short description of part of organism photographed
copyright_years	The year of the licence declaration for the image
copyright_licences	Type of licence associated with the image
copyright_institutions	The primary licence holder's institutional affiliation
photographers	The individual responsible for photographing

## Additional information

### Summary statistics and genetic distances

The dataset contains on average 2.6 records per species, ranging from one (184 species) to 12 (one species) records per species. Average sequence length was 541 bp (including sequences that did not include the whole ITS region due to unreadable trace files). The shortest sequence in the dataset was an incomplete sequence, 229 bp long. The species with the shortest complete ITS region was *Haematomma ocroloeucum* with 481 bp. The longest ITS region was 856 bp from *Leptogium cochleatum*. However, the two sequences of the latter species did not align with the others at the start of the sequence and it is possible that introns in the SSU rDNA are included.

We calculated intra- and interspecific genetic distances for the three genera with most sampled species and performed barcode gap analyses on those. Distances and key statistics are found in Table 2.

In *Ramalina*, there was an overlap between intra- and interspecific distances (Table 2, Fig. 4a). The overlap is caused by the one species pair with higher maximum intraspecific distance than the distance to the nearest neighbour (Fig. 4b); i.e. *R. farinacea* had an intraspecific distance of 0.9% and a distance of 0.4% to *R. subfarinacea*. If this species pair is excluded from the distance-dataset, a barcode gap will occur at 2% (Fig. 4a).

For *Umbilicaria*, there were no species pairs with higher maximum intraspecific distance than the distance to the nearest neighbour, but one species had the same maximum intraspecific distance as the distance to the nearest neighbour, namely *U. proboscidea* whose closest neighbour was *U. virginis* (Fig. 4d). There is thus no barcode gap in our *Umbilicaria* dataset, as the intra- and interspecific distances overlaps around 3% (Fig. 4c).

Whereas *Ramalina* and *Umbilicaria*, despite the above-mentioned issues, show a tendency for displaying a barcode gap, *Cladonia* does not show the same pattern. There is no gap, but rather overlapping, smooth curves of intra- and interspecific distances in the barplot (Table 2, Fig. 4e). Most of the species pairs that disrupt the pattern are complexes where species are known to be sometimes difficult to distinguish with traditional characters and these are found below the line in Fig. 4f: *C. coniocraea/gracilis*, *C. maxima/ecmocyna* and *C. cenotea/subfurcata/crispata/squamosa*.

ITS has previously been shown to give poor resolution amongst *Cladonia* species (Fontaine et al. 2010, Kotelko and Piercey-Normore 2010, Pino-Bodas et al. 2013) and a lack of barcode gap has been demonstrated (Kelly et al. 2011). This probably has to do with their high morphological plasticity (Pino-Bodas et al. 2013) which may have led to erroneous identifications/taxonomy, but there might also be evolutionary explanations, such as incomplete lineage sorting (van Velzen et al. 2012).

### Observations

From the phylogenetically and BLASTn based quality control, some specific taxonomic observations were made that are listed below.

**Figure 5.**
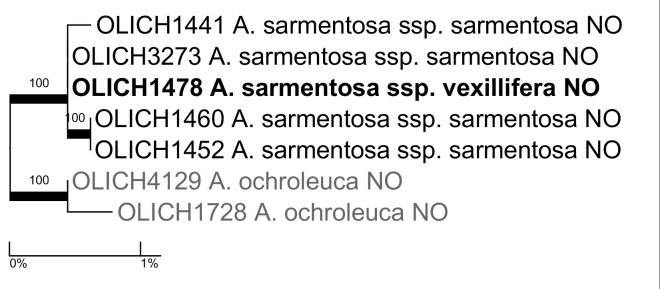
Maximum likelihood tree based on the OLICH sequences of *Alectoria sarmentosa*. Numbers above branches and thick lines indicate bootstrap support ≥ 70%. Bold font indicates focus taxon and grey font the outgroup. The two-letter ISO country codes are included. This applies for all phylogram figures (Figs 5, 6, 7, 8, 9, 10, 11, 12, 13, 14, 15).

**Alectoria sarmentosa (Ach.) Ach. ssp. vexillifera (Nyl.) D.Hawksw.**: This subspecies is currently accepted in the Nordic flora (Velmala and Myllys 2011, Nordin et al. 2019). It differs morphologically from spp. *sarmentosa* in forming a more prostrate thallus with broader and more flattened main branches and, ecologically, in growing on the ground or on rocks mainly in coastal and alpine areas. Subspecies *sarmentosa* grows mainly on trunks and branches of conifers in boreal forests. Our sequence of spp. *vexillifera* (OLICH1478) is nested within our four sequences of spp. *sarmentosa*, however, which does not support the recognition of the subspecies (Fig. 5).

**Figure 6.**
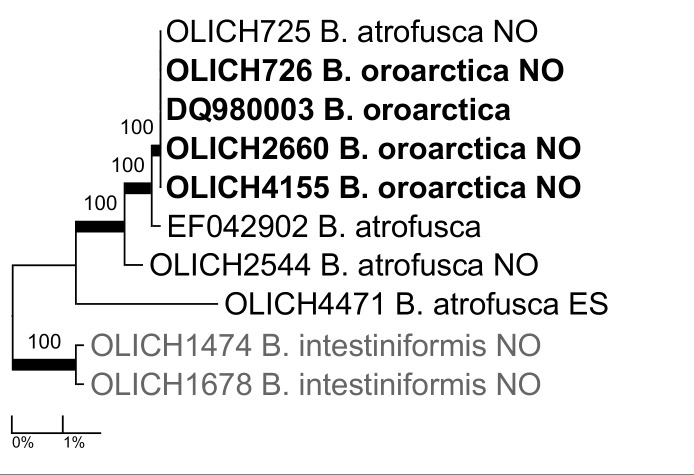
Maximum likelihood tree of *Brodoa atrofusca* and *B. oroarctica* based on all available ITS sequences.

***Brodoa oroarctica* (Krog) Goward**: The species differs from *B. atrofusca* (Schaer.) Goward in the shape of the thallus and lobes and in the lack of physodic acid in most of the thallus except for in some lobe tips (Krog 1974). The former species is circumpolar arctic-alpine, with southernmost occurrences in Europe in the southern Scandinavian mountains, whereas the latter is a European species known mainly from the Alps and Pyrenées and with scattered occurrences in Northern Europe. Our four barcode sequences of *B. oroarctica* are however nested within the four sequences of *B. atrofusca* (Fig. 6), and the complex seems to be in need of revision.

***Bryobilimbia fissuriseda* (Poelt) Timdal, Marthinsen & Rui, comb. nov.**

Mycobank: MB 830330

Basionym: *Lecidea fissuriseda* Poelt, Mitteilungen der Botanischen Staatssammlung München 4: 181, 1961. ≡ *Mycobilimbia fissuriseda* (Poelt) Poelt & Hafellner, Herzogia 8: 56, 1989. Type: Switzerland, Berner Oberland, Gipfelgrader Niesen, Nordhänge, alt. 2300 m, 1955, Poelt B55189 (M!, holotype).

**Figure 7.**
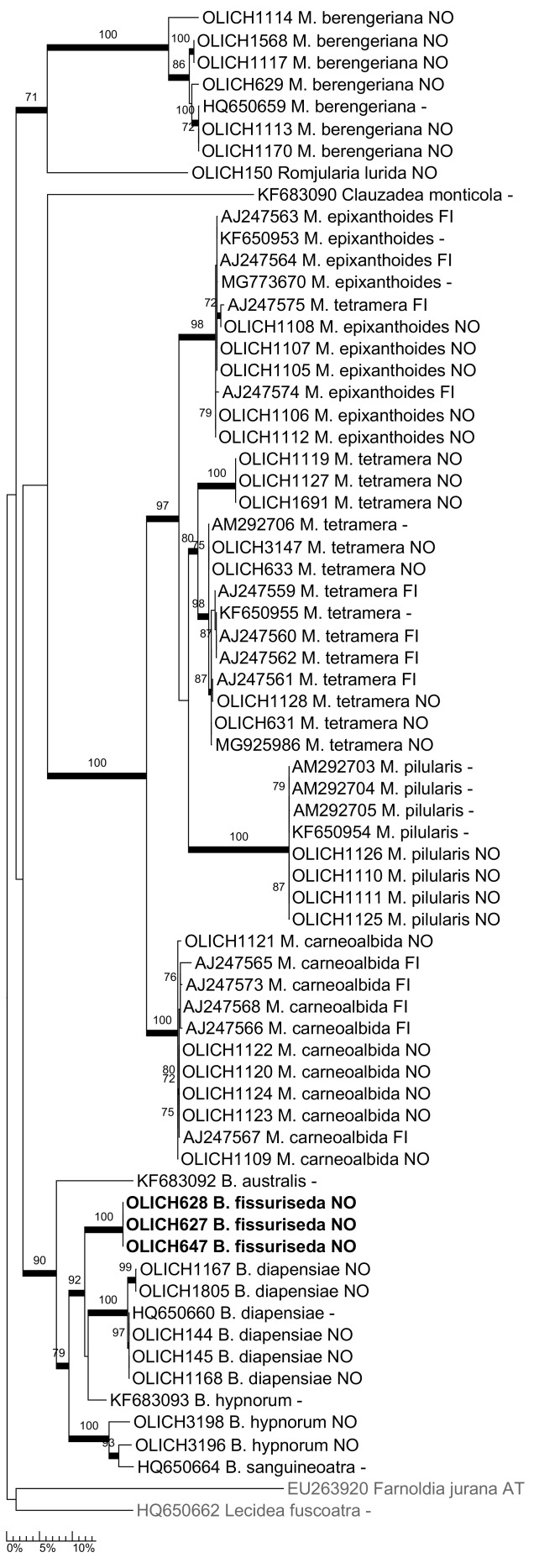
Maximum likelihood tree of *Bryobilimbia* and *Mycobilimbia* based on all available ITS sequences. The presumedly related genera *Clauzadea* and *Romjularia* are included and *Farnoldia* and *Lecidea* are used as the outgroup.

This rarely collected species was described from the Alps in the large, collective genus *Lecidea* Ach. (Poelt 1961). It was later transferred to the segregate genus *Mycobilimbia* Rehm by Poelt & Hafellner (in Hafellner 1989). Partly due to the presence of characteristic blue, K+ green granules in the hymenium and the hypothecium, Poelt (1961) and Timdal (1992) assumed it was closely related to *M. hypnorum* (Lib.) Kalb & Hafellner (syn. *Lecidea fusca* (Schaer.) Th. Fr.). Fryday et al. (2014), based on a molecular study, excluded *M. hypnorum* from *Mycobilimbia* and placed it, with four additional species, in the new genus *Bryobilimbia* Fryday et al. *Mycobilimbia fissuriseda* was not included in that study, but our phylogenetic tree, based on the DNA barcodes (Fig. 7), shows that it is, in fact, closely related to *B. diapensiae* and *B. hypnorum* and it is hence here transferred to *Bryobilimbia*.

**Figure 8.**
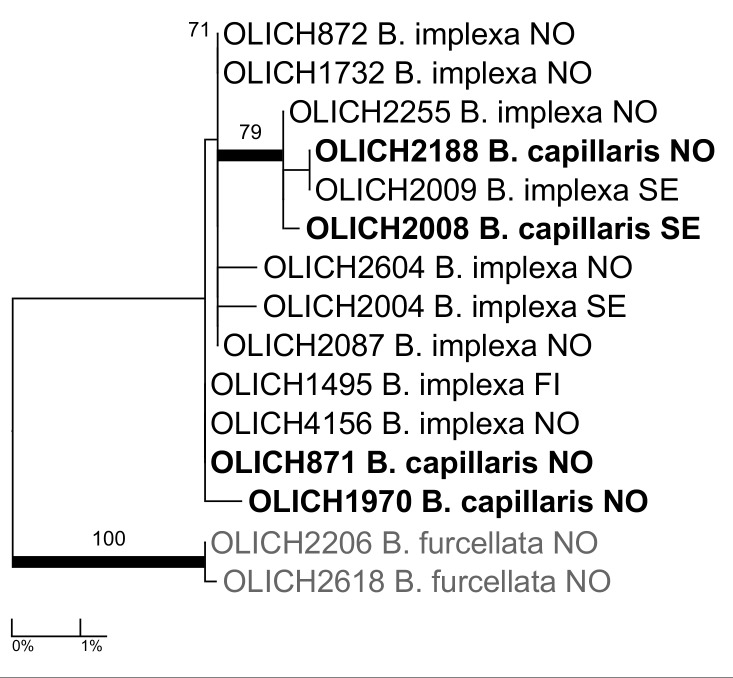
Maximum likelihood tree of *Bryoria capillaris* and *B. implexa* based on the OLICH sequences.

***Bryoria capillaris* (Ach.) Brodo & D.Hawksw.**: The species is still accepted in the Nordic checklist (Nordin et al. 2019), despite several recent molecular phylogenies (Myllys et al. 2011, Velmala et al. 2014, Myllys et al. 2016, Boluda et al. 2019) showing it nested within *B. implexa* (Hoffm.) Brodo & D. Hawksw. The OLICH dataset shows the same topography (Fig. 8): *B. capillaris* nested within *B. implexa*. However, all our specimens of the *B. capillaris/implexa* complex were identified by morphology prior to TLC analysis and the correlation holds: all pale specimens (*B. capillaris*) contain alectorialic acid (plus accessories) and barbatolic acid, whereas no dark specimens (*B. implexa*) do. Fumarprotocetraric and protocetraric acids occur variably in both species. Either the DNA barcode marker and the other markers used in recent phylogenetic studies do not distinguish between two species which may be recognised by the correlation of thallus colour and secondary chemistry or the two "species" are conspecific and their morphological/chemical difference is caused by other factors. For example, the presence/absence of an additional symbiont (basidiomycete yeast) were found likely to trigger the production of vulpinic acid in the cortex of Bryoria fremontii/tortuosa by Spribille et al. (2016).

**Figure 9.**
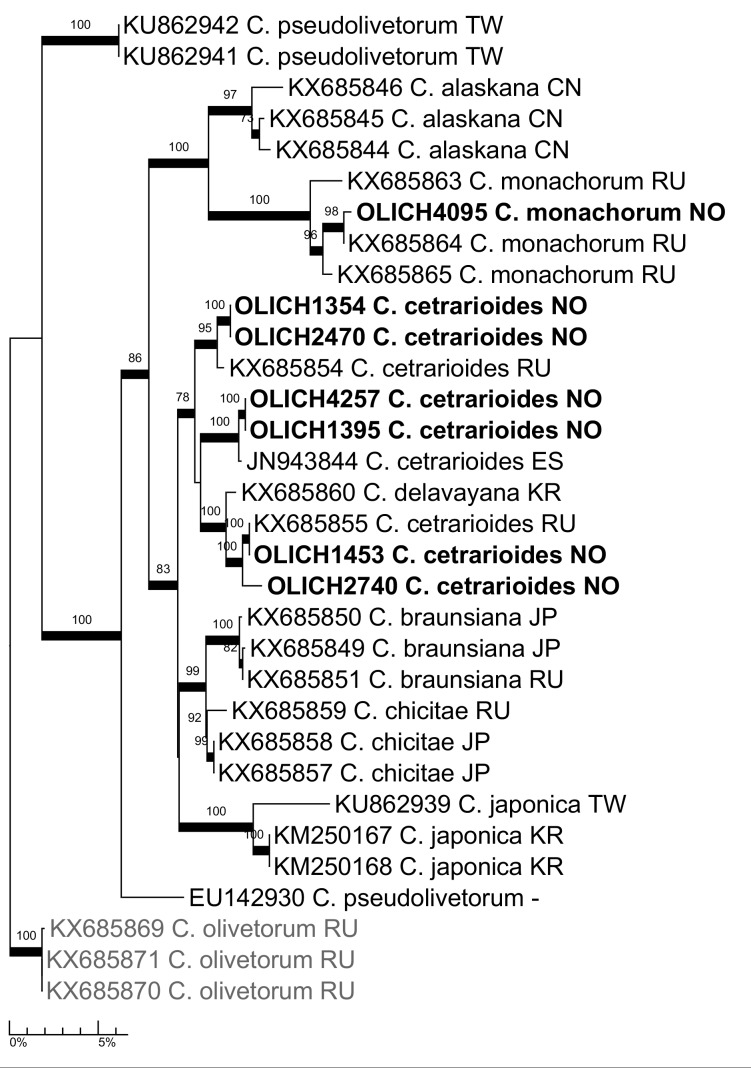
Maximum likelihood tree of the OLICH sequences of *Cetrelia*, with selected ITS sequences from Mark et al. (2019).

***Cetrelia cetrarioides* (Duby) W.L. Culb. & C.F. Culb. and *C. monachorum* (Zahlbr.) W.L. Culb. & C.F. Culb.**: The three chemotypes of *Cetrelia olivetorum* (Nyl.) W.L. Culb. & C.F. Culb. s. lat., currently recognised in the Nordic countries (Bjelland et al. 1997, Obermayer and Mayrhofer 2007, Nordin et al. 2019), were recently shown to represent three distinct species in the phylogenetic analysis by Mark et al. (2019). The diagnostic, main compounds of those species are imbricaric acid in *C. monachorum*, olivetoric acid in *C. olivetorum* s. str. and perlatolic acid in *C. cetrarioides* (Mark et al. 2019). The seven specimens sequenced in the OLICH project fell into the *C. cetrarioides* and *C. monachorum* clades (Fig. 9) and the TLC analyses of the sequenced specimens showed the chemotypes were in accordance with the species identifications (Table 1).

**Figure 10.**
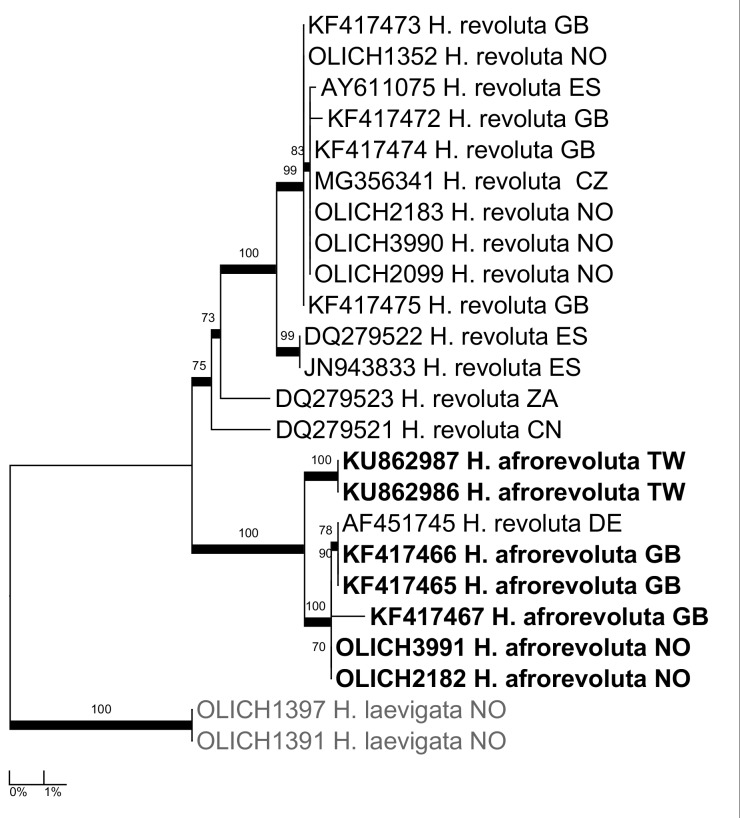
Maximum likelihood tree of the OLICH sequences of *Hypotrachyna afrorevolulta* and *H. revoluta*, with all available ITS sequences in GenBank.

***Hypotrachyna afrorevoluta* (Krog & Swinscow) Krog & Swinscow**: The species is morphologically and chemically quite similar to *H. revoluta* (Flörke) Hale and the OLICH sequences (Fig. 10) confirm the presence of the two species in the Nordic countries. See Elix and Thell (2011) for the subtile morphological differences between the species.

**Figure 11.**
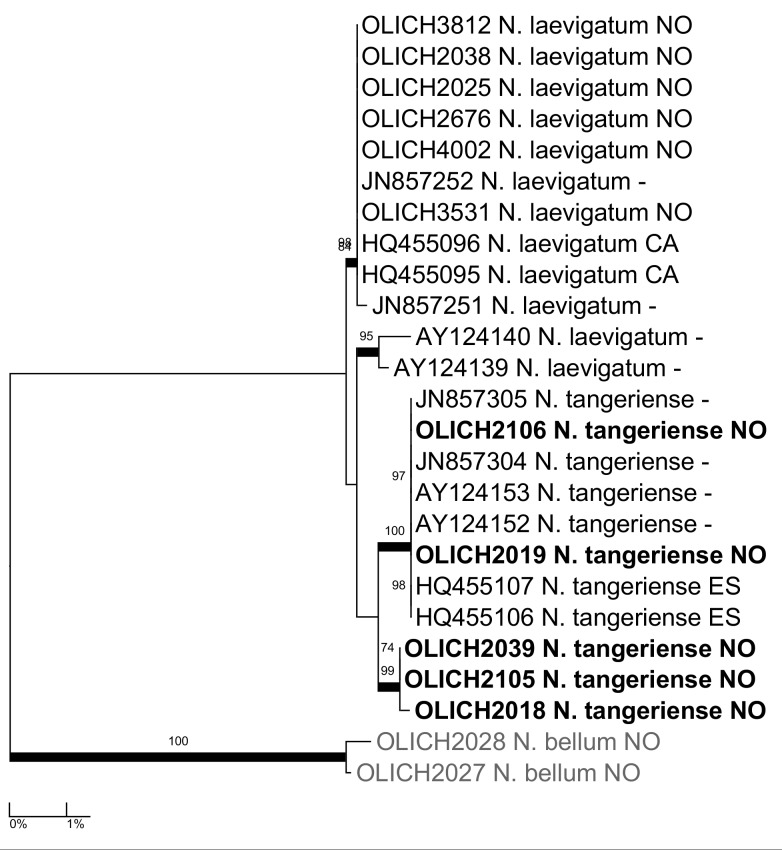
Maximum likelihood tree based on the OLICH sequences of *Nephroma laevigatum* and *N. tangeriense*, with selected ITS sequences from GenBank.

***Nephroma tangeriense* (Maheu & A.Gillet) Zahlbr.**: The species was recently reported as new to the Nordic countries (Klepsland 2013) from two collections from Rogaland county. The OLICH project has sequenced one of those (OLICH2019), as well as four additional collections from Hordaland and Vest-Agder counties and confirms its identity and distinction from *N. laevigatum* Ach. (Fig. 11).

***Peltigera “neocanina***”: BLASTn search revealed the identity of OLICH1878, which was originally identified as *P. canina* (L.) Willd., as *P.* “*neocanina*”. This is still an invalid name despite the taxon, which must be regarded as a morphologically cryptic species, has been known since the phylogenetic study of *Peltigera* by Miadlikowska and Lutzoni (2000). Jüriado et al. (2017) showed its presence in Estonia and Magain et al. (2018) in Norway.

**Figure 12.**
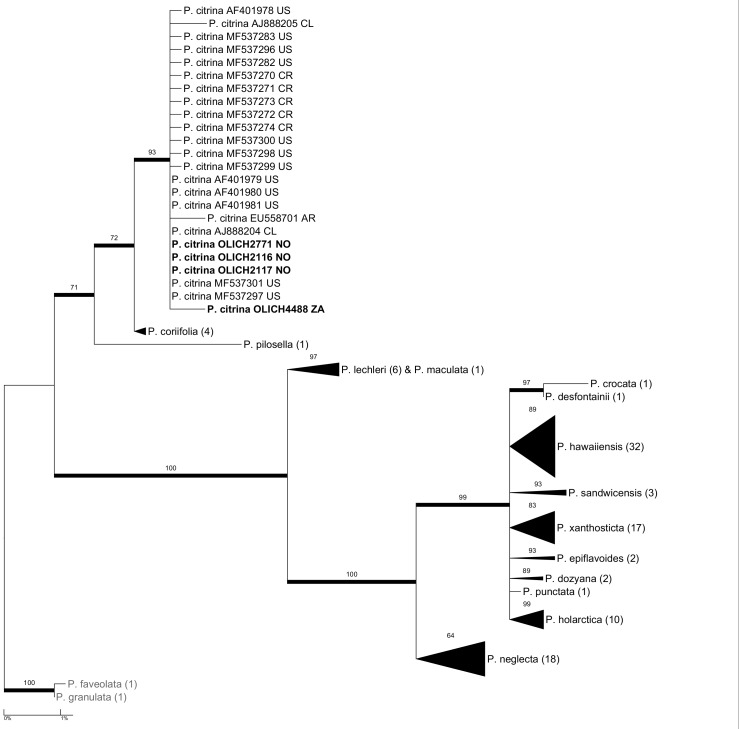
Maximum likelihood tree of the *Pseudocyphellaria crocata* complex with the OLICH sequences added to those of Lücking et al. (2017b).

***Pseudocyphellaria citrina* (Gyeln.) Lücking, Moncada & S. Stenroos**: The *Pseudocyphellaria* species with yellow soralia, occurring in the Nordic countries, is currently named *P. crocata* (L.) Vain. (Nordin et al. 2019). Lücking et al. (2017b) showed that *P. crocata* was heterogeneous and split it into 13 species. European material was not included in that study and it remained unknown which species occurs in our area. The sequences obtained from three Norwegian specimens in the OLICH project all belong in the *P. citrina* clade of Lücking et al. (2017b) (Fig. 12).

**Figure 13.**
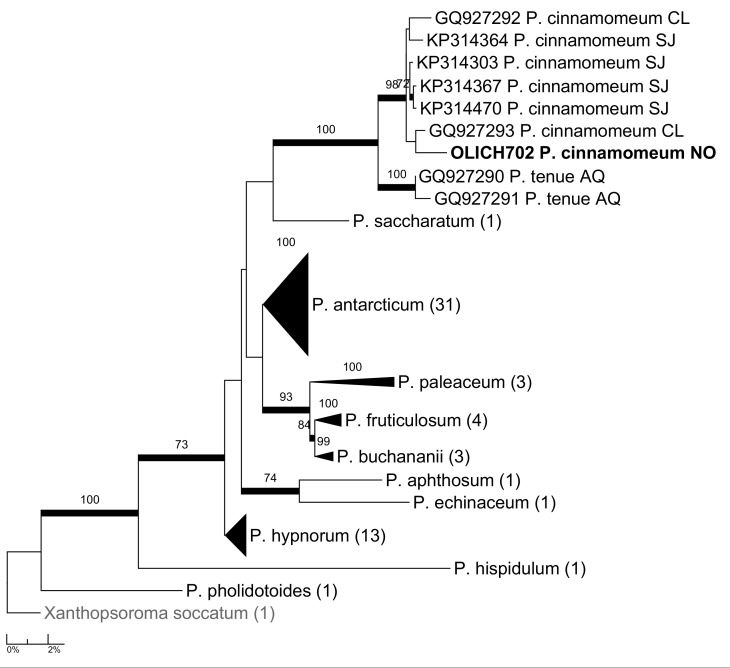
Maximum likelihood tree of *Psoroma* based on all available ITS sequences.

***Psoroma cinnamomeum* Malme**: The specimen from which OLICH702 was generated was originally identified as Psoroma tenue Henssen var. boreale Henssen, but BLASTn search against GenBank indicated its identity as *P. cinnamomeum*. A phylogenetic analysis, based on most of the ITS sequences of *Psoroma* in GenBank (including those used by Park et al. 2018), supported this identification as OLICH702 and came out in a strongly supported clade with the six other specimens of *P. cinnamomeum*, two of which are from southernmost Chile and four from Svalbard (Fig. 13). The clade was the sister of the strongly supported clade of the two specimens of *P. tenue* in GenBank, both from Antarctica. Both *P. cinnamomeum* and *P. tenue* were described from Tierra del Fuego, var. *boreale* from USA, Colorado (cf. Henssen and Renner 1981). OLICH702 contains porphyrilic acid and two unknown compounds (apparently related to porphyrilic acid due to the colour and fluorescence on the chromatograms) and hence fits the current concept of P. tenue var. boreale (cf. Jørgensen 2007). Unless molecular studies show that there are two morphologically very similar species in the Northern Hemisphere, both containing porphyrilic acid, we believe *P. cinnamomeum* is the correct name for the taxon currently known as P. tenue var. boreale.

**Figure 14.**
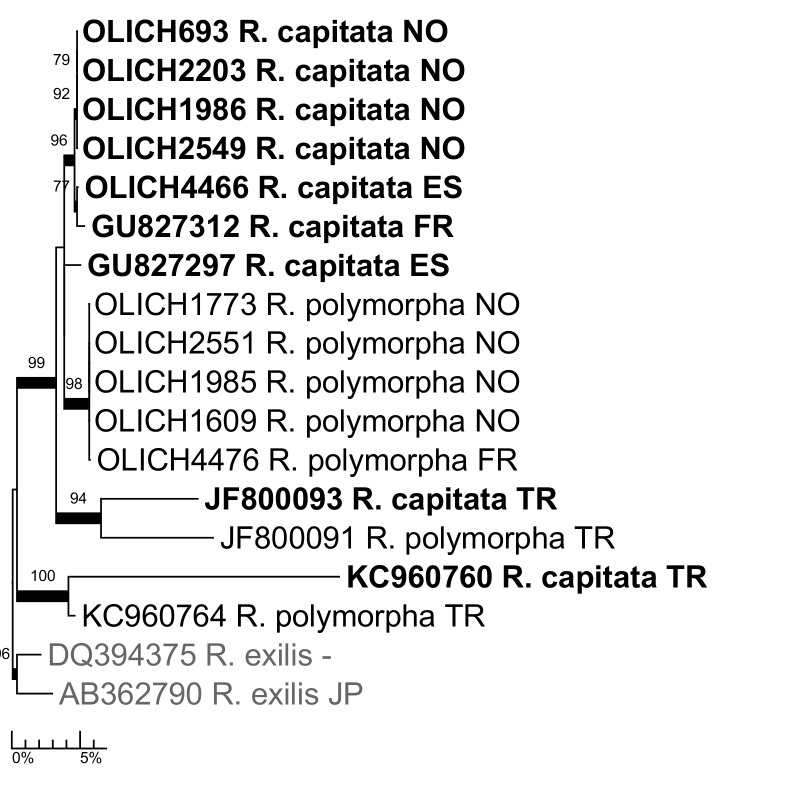
Maximum likelihood tree of the *Ramalina capitata/polymorpha* complex, based on all available ITS sequences.

***Ramalina capitata* (Ach.) Nyl.**: The species has often been included in *R. polymorpha* (Lilj.) Ach. in the past (e.g. Krog and James 1977), but is now usually accepted as distinct (e.g. Nimis et al. 2018, Nordin et al. 2019). In our phylogeny (Fig. 14), Nordic and western European material of the two species are mostly well separated, but the four sequences from Turkey in GenBank are deviant and the complex seems to be in need of further study.

**Figure 15.**
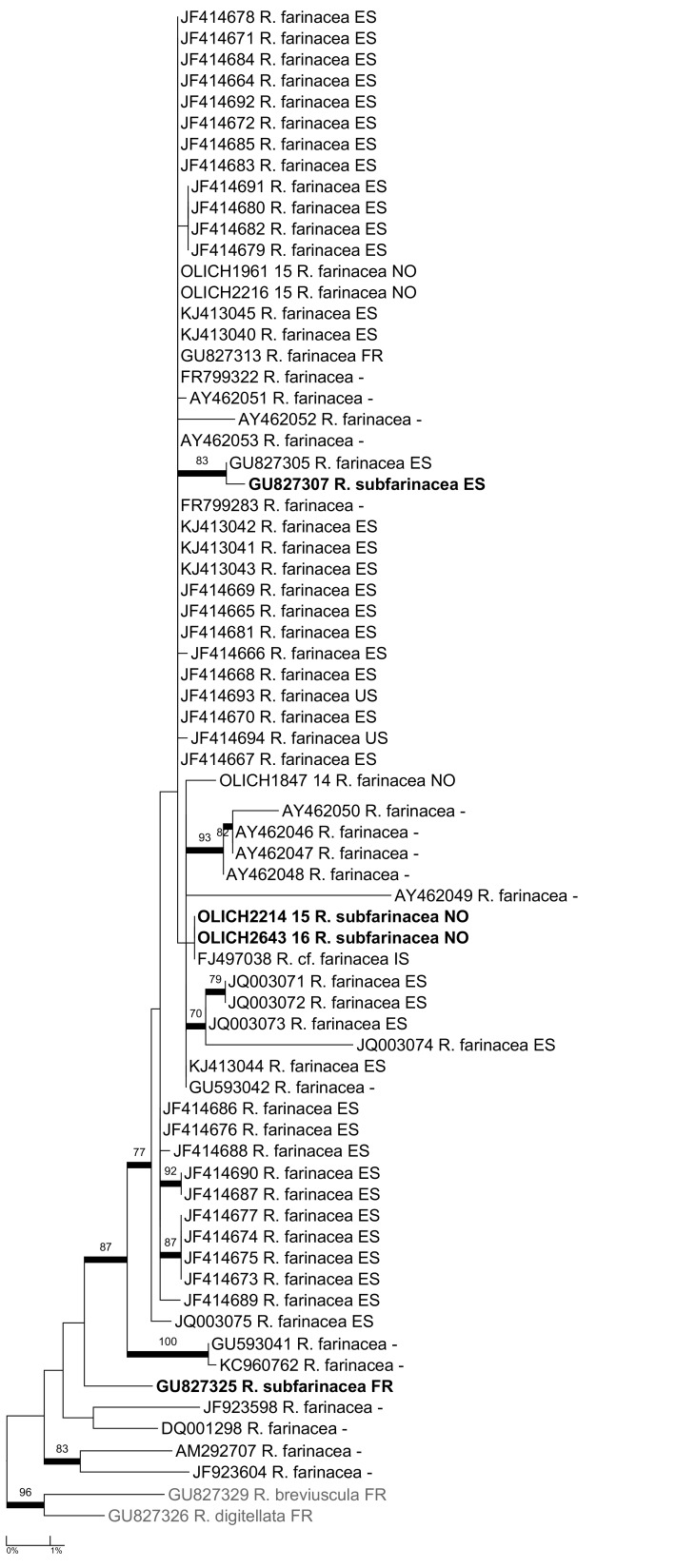
Maximum likelihood tree of the *Ramalina farinacea/subfarinacea* complex, based on all available ITS sequences.

***Ramalina subfarinacea* (Cromb.) Nyl.**: Our two sequences of this species, as well as the two ITS sequences available in GenBank, are nested in the clade of *Ramalina farinacea* (L.) Ach. in our phylogeny (Fig. 15). According to Krog and James (1977), there are subtle morphological differences between the species, but apparently the major distinguishing feature is the ecology: mainly on coastal rock (*R. subfarinacea*) vs. mainly on bark of deciduous, inland trees (*R. farinacea*). More study is needed in order to evaluate the taxonomic status of *R. subfarinacea*.

***Sticta fuliginoides* Magain & Sérus.**: BLASTn search of the sequence of an originally identified *Sticta fuliginosa* (Hoffm.) Ach. (OLICH2638) showed 99.4–100% similarity with the five sequences of *S. fuliginoides* and 90.0–90.2% similarity with the 9 sequences of *S. fuliginosa* in the phylogeny of Magain and Serusiaux (2015). This confirms the presence of *S. fuliginoides* in the Nordic countries, as recently reported by Ekman et al. (2019), based on morphological characters.

## Supplementary Material

749C9139D56D57DA9A1455455E5205FA 10.3897/BDJ.7.e36252.suppl1 Supplementary material 1 OLICH library - Processing details Data type: Record information - processing details on ITS sequences
Brief description: The file includes information about all records in BOLD for the OLICH library, i.e. the DS-OLICH dataset, at time of writing. It contains data on ITS sequences. The data are as downloaded from BOLD without further processing.
File: oo_302451.txt https://binary.pensoft.net/file/302451 Gunnhild Marthinsen, Siri Rui, Einar Timdal

C8B3A057995A58D6B2C15B920307D30D 10.3897/BDJ.7.e36252.suppl2 Supplementary material 2 OLICH library - Specimen details Data type: Record information - specimen data
Brief description: The file includes information about all records in BOLD for the OLICH library, i.e. the DS-OLICH dataset, at time of writing. It contains collecting and identification data. The data are as downloaded from BOLD, without further processing.
File: oo_302452.txt https://binary.pensoft.net/file/302452 Gunnhild Marthinsen, Siri Rui, Einar Timdal

EE8E842DF6EF58A5A30FC8BBCAAA1156 10.3897/BDJ.7.e36252.suppl3 Supplementary material 3 OLICH library - Specimen details - Darwin Core Standard Data type: Record information - specimen data in Darwin Core Standard format
Brief description: The file includes information about all records in BOLD for the OLICH library, i.e. the DS-OLICH dataset, at time of writing. It contains collecting and identification data. The data are downloaded from BOLD and further formatted to the DC standard by the authors.
File: oo_302453.txt https://binary.pensoft.net/file/302453 Gunnhild Marthinsen, Siri Rui, Einar Timdal

8E458F215B4B562D9C9DA61F4F7C6882 10.3897/BDJ.7.e36252.suppl4 Supplementary material 4 OLICH library - DNA sequences Data type: Genomic data, DNA sequences
Brief description: ITS sequences in fasta format. Each sequence is identified by the BOLD ProcessID and species name, separated by pipe. The data are as downloaded from BOLD.
File: oo_301034.fas https://binary.pensoft.net/file/301034 Gunnhild Marthinsen, Siri Rui, Einar Timdal

## Figures and Tables

**Figure 4a.**
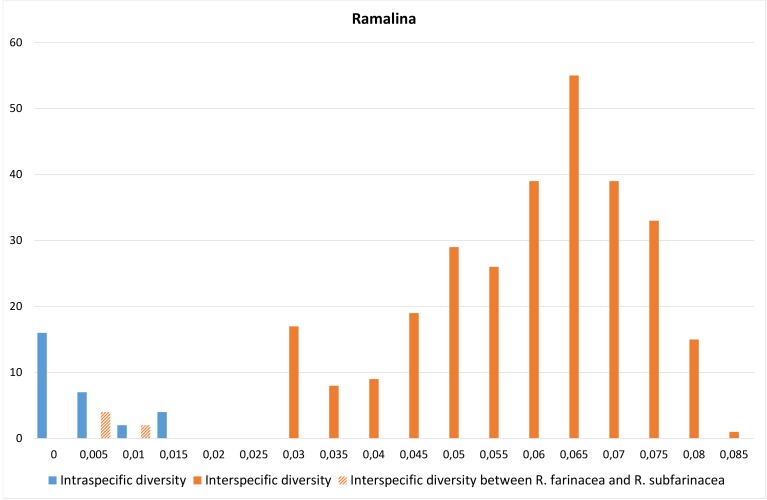
Frequency distribution of intraspecific distances (blue) and interspecific distances (orange). A gap between intra- and interspecific distances illustrates the barcode gap in the genus.

**Figure 4b.**
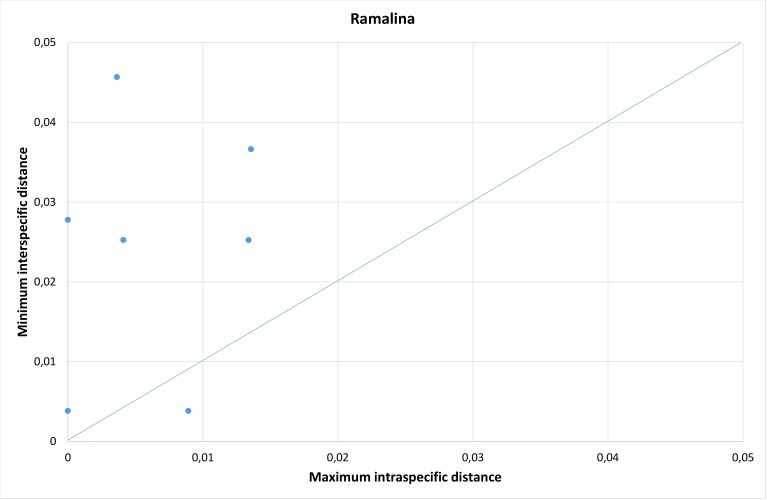
Scatterplots representing maximum intraspecific distances versus minimum distance to closest neighbour for all species with more than one specimen per species, in each of the three genera.

**Figure 4c.**
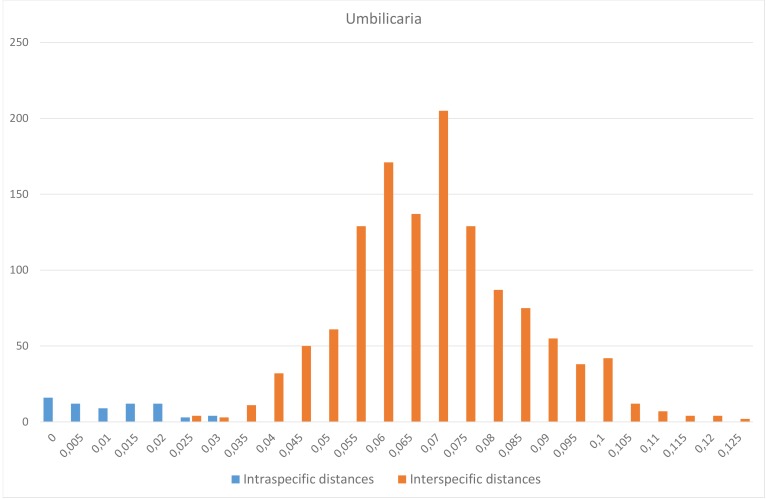
Frequency distribution of intraspecific distances (blue) and interspecific distances (orange). A gap between intra- and interspecific distances illustrates the barcode gap in the genus.

**Figure 4d.**
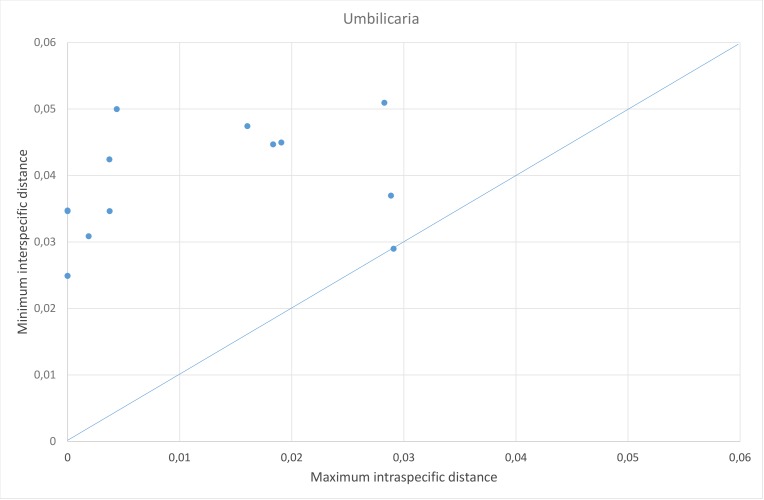
Scatterplots representing maximum intraspecific distances versus minimum distance to closest neighbour for all species with more than one specimen per species, in each of the three genera.

**Figure 4e.**
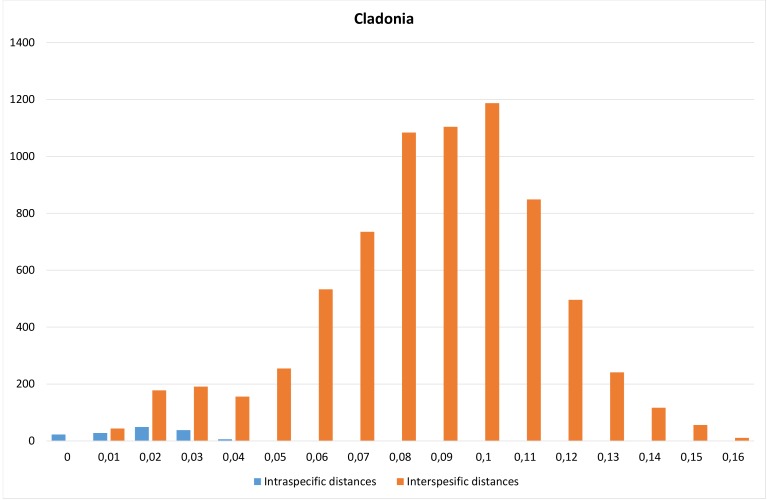
Frequency distribution of intraspecific distances (blue) and interspecific distances (orange). A gap between intra- and interspecific distances illustrates the barcode gap in the genus.

**Figure 4f.**
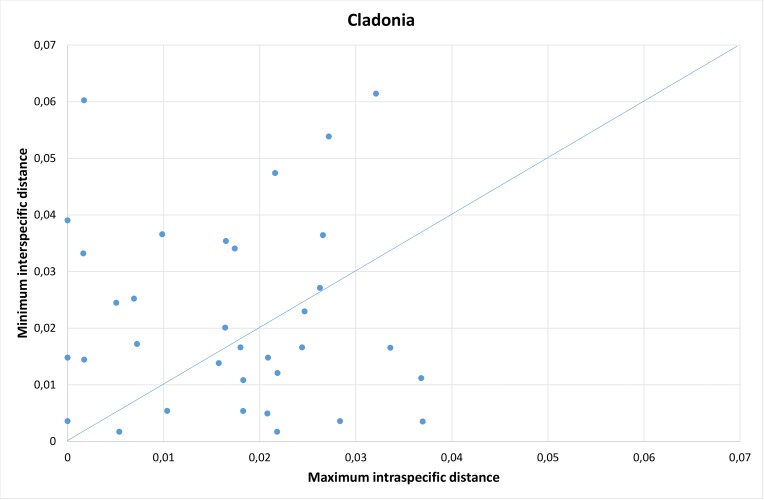
Scatterplots representing maximum intraspecific distances versus minimum distance to closest neighbour for all species with more than one specimen per species, in each of the three genera.

**Table 1.** List of specimens included in the OLICH dataset (sequence length >200bp, 1324 records, 507 species). Thin Layer Chromotagrophy (TLC, Chemistry-column) was only done in species complexes where chemical data is relevant for the identification of the species or where there are chemical strains within the species.

**Species**	**Order**	**Voucher**	**Sample ID**	**Process ID**	**GenBank ID**	**Chemistry**
*Acarospora glaucocarpa*	Acarosporales	NO, Nord-Trøndelag, 2015, R. Haugan	O-L-201295	OLICH3852-17	MK811746	
*Acarospora molybdina*	Acarosporales	NO, Finnmark, 2014, E. Timdal	O-L-195556	OLICH1514-14	KY266941	
*Acarospora wahlenbergii*	Acarosporales	NO, Hedmark, 2013, E. Timdal	O-L-184511	OLICH737-13	MK811773	
*Acarospora wahlenbergii*	Acarosporales	NO, Oppland, 2013, M. Bendiksby et al.	O-L-184363	OLICH742-13	MK812641	
*Acarospora wahlenbergii*	Acarosporales	NO, Telemark, 2013, E. Timdal	O-L-184419	OLICH870-13	MK811926	
*Acolium inquinans*	Caliciales	NO, Nordland, 2012, J.T. Klepsland	O-L-186246	OLICH2077-15	MK812481	
*Acolium inquinans*	Caliciales	NO, Buskerud, 2012, T.H. Hofton	O-L-194494	OLICH2080-15	MK811701	
*Acolium inquinans*	Caliciales	NO, Aust-Agder, 2014, J.T. Klepsland	O-L-200169	OLICH3168-16	MK812233	
*Acolium inquinans*	Caliciales	NO, Hedmark, 2014, R. Haugan	O-L-207991	OLICH3827-17	MK811747	
*Alectoria nigricans*	Lecanorales	NO, Troms, 2014, E. Timdal	O-L-195780	OLICH1652-14	MK812557	
*Alectoria nigricans*	Lecanorales	NO, Finnmark, 2014, M. Westberg	O-L-195858	OLICH1703-14	KY266967	
*Alectoria nigricans*	Lecanorales	NO, Finnmark, 2014, E. Timdal	O-L-195886	OLICH1731-14	KY266922	
*Alectoria nigricans*	Lecanorales	NO, Finnmark, 2014, H. Holien	O-L-195950	OLICH1795-14	KY266831	
*Alectoria nigricans*	Lecanorales	NO, Buskerud, 2014, S. Rui & E. Timdal	O-L-196314	OLICH2244-15	MK812235	
*Alectoria nigricans*	Lecanorales	NO, Buskerud, 2015, S. Rui & E. Timdal	O-L-200895	OLICH2548-16	MK812358	
*Alectoria ochroleuca*	Lecanorales	NO, Finnmark, 2014, R. Haugan	O-L-195746	OLICH1618-14	KY266936	
*Alectoria ochroleuca*	Lecanorales	NO, Finnmark, 2014, E. Timdal	O-L-195883	OLICH1728-14	KY266959	
*Alectoria ochroleuca*	Lecanorales	NO, Buskerud, 2014, E. Timdal	O-L-195823	OLICH2195-15	MK811939	
*Alectoria ochroleuca*	Lecanorales	NO, Buskerud, 2015, S. Rui & E. Timdal	O-L-200894	OLICH2547-16	MK812226	
*Alectoria ochroleuca*	Lecanorales	NO, Oppland, 2012, K.A. Lye	O-L-206884	OLICH4129-17	MK812278	
*Alectoria sarmentosa*	Lecanorales	NO, Nord-Trøndelag, 2012, J.T. Klepsland	O-L-186255	OLICH1441-14	MK812505	
*Alectoria sarmentosa*	Lecanorales	NO, Sogn og Fjordane, 2009, T.H. Hofton	O-L-186870	OLICH1452-14	MK812223	
*Alectoria sarmentosa*	Lecanorales	NO, Østfold, 2013, B.P. Løfall & O. Wergeland Krog	O-L-187364	OLICH1460-14	MK812403	
*Alectoria sarmentosa*	Lecanorales	NO, Sogn og Fjordane, 2014, K.J. Grimstad & O. Olsen	O-L-193185	OLICH1478-14	MK812717	
*Alectoria sarmentosa*	Lecanorales	NO, Sør-Trøndelag, 2014, S. Vatne	O-L-200758	OLICH3273-16	MK812509	
*Allantoparmelia alpicola*	Lecanorales	NO, Finnmark, 2014, R. Haugan	O-L-195748	OLICH1620-14	KY266874	
*Allantoparmelia alpicola*	Lecanorales	NO, Finnmark, 2014, E. Timdal	O-L-195850	OLICH1695-14	KY266926	
*Allantoparmelia alpicola*	Lecanorales	NO, Finnmark, 2014, E. Timdal	O-L-195899	OLICH1744-14	KY266946	
*Allantoparmelia alpicola*	Lecanorales	NO, Buskerud, 2014, S. Rui & E. Timdal	O-L-196324	OLICH2254-15	MK811991	
*Allantoparmelia alpicola*	Lecanorales	NO, Oppland, 2015, E. Timdal	O-L-201391	OLICH4173-17	MK811876	
*Anaptychia ciliaris*	Caliciales	NO, Aust-Agder, 2012, J.T. Klepsland	O-L-186030	OLICH1419-14	MK812701	
*Anaptychia ciliaris*	Caliciales	NO, Hedmark, 2011, T.H. Hofton	O-L-194359	OLICH1483-14	MK812099	
*Anaptychia ciliaris*	Caliciales	NO, Finnmark, 2014, E. Timdal	O-L-195591	OLICH1532-14	MK812249	
*Anaptychia ciliaris*	Caliciales	NO, Finnmark, 2014, G. Thor	O-L-195736	OLICH1608-14	KY266912	
*Anaptychia ciliaris*	Caliciales	NO, Akershus, 2014, E. Timdal	O-L-199982	OLICH1956-15	MK812295	
*Anaptychia ciliaris*	Caliciales	NO, Oslo, 2014, E. Timdal	O-L-198929	OLICH2482-16	MK812296	
*Anaptychia runcinata*	Caliciales	NO, Finnmark, 2014, E. Timdal	O-L-195767	OLICH1639-14	MK812680	
*Anaptychia runcinata*	Caliciales	NO, Sør-Trøndelag, 2014, R. Haugan	O-L-196019	OLICH1864-14	MK811742	
*Anaptychia runcinata*	Caliciales	NO, Østfold, 2016, S. Rui & E. Timdal	O-L-208017	OLICH4189-17	MK811645	
*Arctocetraria andrejevii*	Lecanorales	NO, Finnmark, 2012, H. Henriksen	O-L-180951	OLICH869-13	MK811992	norrangiformic acid (minor), rangiformic acid (major)
*Arctoparmelia centrifuga*	Lecanorales	NO, Troms, 2014, R. Haugan	O-L-198774	OLICH2399-16	MK812567	
*Arctoparmelia centrifuga*	Lecanorales	NO, Telemark, 2015, S. Rui & E. Timdal	O-L-200879	OLICH2536-16	MK812513	
*Arctoparmelia centrifuga*	Lecanorales	NO, Buskerud, 2013, S. Rui & E. Timdal	O-L-184727	OLICH942-13	MK812611	
*Arctoparmelia incurva*	Lecanorales	NO, Finnmark, 2014, T. Tønsberg	O-L-195717	OLICH1589-14	KY266852	
*Arctoparmelia incurva*	Lecanorales	NO, Buskerud, 2014, E. Timdal	O-L-195832	OLICH2204-15	MK811976	
*Arthonia didyma*	Arthoniales	NO, Nord-Trøndelag, 2013, J.T. Klepsland	O-L-197934	OLICH4022-17	MK812348	
*Arthonia phaeobaea*	Arthoniales	NO, Sør-Trøndelag, 2013, J.T. Klepsland	O-L-198129	OLICH3187-16	MK812215	
*Arthopyrenia salicis*	Pleosporales	NO, Nord-Trøndelag, 2016, J.T. Klepsland	O-L-206752	OLICH3471-17	MK812299	
*Arthopyrenia salicis*	Pleosporales	NO, Møre og Romsdal, 2016, J.T. Klepsland	O-L-206774	OLICH3493-17	MK812520	
*Asahinea chrysantha*	Lecanorales	NO, Finnmark, 2014, E. Timdal	O-L-195754	OLICH1626-14	KY266872	
*Bacidia bagliettoana*	Lecanorales	NO, Akershus, 2009, R. Haugan	O-L-161668	OLICH282-13	MG838180	
*Bacidia bagliettoana*	Lecanorales	NO, Troms, 2009, J.T. Klepsland	O-L-164908	OLICH284-13	MG838178	
*Bacidia subincompta*	Lecanorales	NO, Finnmark, 2011, E. Timdal	O-L-170623	OLICH198-13	MG838157	no lichen substances
*Bacidia subincompta*	Lecanorales	NO, Sogn og Fjordane, 2013, J.T. Klepsland	O-L-197862	OLICH2086-15	MG838186	
*Bacidia subincompta*	Lecanorales	NO, Østfold, 2001, B.P. Løfall	O-L-105331	OLICH310-13	MG838165	
*Bacidia subincompta*	Lecanorales	NO, Oslo, 2014, J.T. Klepsland	O-L-200148	OLICH3180-16	MG838175	
*Bacidia subincompta*	Lecanorales	NO, Nordland, 2015, J.T. Klepsland	O-L-206520	OLICH3420-17	MG838176	
*Bacidia vermifera*	Lecanorales	NO, Troms, 2011, J.T. Klepsland	O-L-177162	OLICH264-13	MG838160	
*Bacidia vermifera*	Lecanorales	NO, Troms, 2009, J.T. Klepsland	O-L-165007	OLICH299-13	MG838169	
*Bactrospora corticola*	Arthoniales	NO, Nord-Trøndelag, 2010, H. Holien	TRH-L-13774	OLICH1208-14	MK811961	
*Bactrospora corticola*	Arthoniales	NO, Nord-Trøndelag, 2009, H. Holien	TRH-L-12953	OLICH1209-14	MK811741	
*Bactrospora corticola*	Arthoniales	NO, Nord-Trøndelag, 2009, H. Holien	TRH-L-12974	OLICH1210-14	MK811643	
*Bactrospora corticola*	Arthoniales	NO, Sør-Trøndelag, 2010, G. Gaarder	TRH-L-651772	OLICH1211-14	MK811979	
*Bactrospora corticola*	Arthoniales	NO, Sør-Trøndelag, 2014, S. Vatne	O-L-200761	OLICH2438-16	MK812695	
*Baeomyces carneus*	Baeomycetales	NO, Østfold, 2003, B. P. Løfall	O-L-125189	OLICH4208-17	MK811898	
*Baeomyces carneus*	Baeomycetales	NO, Østfold, 2010, J.T. Klepsland	O-L-174982	OLICH4421-17	MK812113	
*Baeomyces placophyllus*	Baeomycetales	NO, Telemark, 2014, E. Timdal	O-L-196267	OLICH1942-15	MK812574	
*Baeomyces placophyllus*	Baeomycetales	NO, Buskerud, 2014, S. Rui & E. Timdal	O-L-196313	OLICH2243-15	MK812640	
*Baeomyces rufus*	Baeomycetales	NO, Telemark, 2014, S. Rui & E. Timdal	O-L-194160	OLICH1481-14	MK811923	
*Baeomyces rufus*	Baeomycetales	NO, Buskerud, 2014, S. Rui & E. Timdal	O-L-200015	OLICH1989-15	MK812462	
*Baeomyces rufus*	Baeomycetales	NO, Nord-Trøndelag, 2015, M. Bendiksby et al.	O-L-201257	OLICH2765-16	MK812004	
*Baeomyces rufus*	Baeomycetales	NO, Nord-Trøndelag, 2015, M. Bendiksby et al.	O-L-201258	OLICH2766-16	MK812643	
*Baeomyces rufus*	Baeomycetales	NO, Aust-Agder, 2014, J.T. Klepsland	O-L-200581	OLICH3271-16	MK812401	
*Bilimbia lobulata*	Lecanorales	NO, Nordland, 2001, B.P. Løfall	O-L-105772	OLICH469-13	MK812451	
*Bilimbia lobulata*	Lecanorales	NO, Troms, 2009, J.T. Klepsland	O-L-164992	OLICH470-13	MK812712	
*Bilimbia lobulata*	Lecanorales	NO, Nordland, 2000, S. Rui & E. Timdal	O-L-51771	OLICH471-13	MK812122	
*Bilimbia lobulata*	Lecanorales	NO, Oppland, 1995, R. Haugan & E. Timdal	O-L-19936	OLICH630-13	MK812573	
*Bilimbia sabuletorum*	Lecanorales	NO, Buskerud, 2008, J.T. Klepsland	O-L-158388	OLICH206-13	MK812338	
*Bilimbia sabuletorum*	Lecanorales	NO, Telemark, 2010, S. Rui & E. Timdal	O-L-163841	OLICH207-13	MK812048	no lichen substances
*Bilimbia sabuletorum*	Lecanorales	NO, Oslo, 2009, J.T. Klepsland	O-L-164642	OLICH208-13	MK812294	
*Bilimbia sabuletorum*	Lecanorales	NO, Aust-Agder, 2010, J.T. Klepsland	O-L-175077	OLICH209-13	MK812254	
*Bilimbia sabuletorum*	Lecanorales	NO, Hedmark, 2004, R. Haugan	O-L-131717	OLICH632-13	MK811845	
*Blastenia ammiospila*	Teloschistales	NO, Finnmark, 2014, U. Arup	O-L-195889	OLICH1734-14	KY266873	
*Blastenia crenularia*	Teloschistales	NO, Sør-Trøndelag, 2014, R. Haugan	O-L-196018	OLICH1863-14	MK811924	
*Blastenia crenularia*	Teloschistales	NO, Nordland, 2014, J.T. Klepsland	O-L-200301	OLICH3173-16	MK811921	
*Blastenia crenularia*	Teloschistales	NO, Akershus, 2013, S. Rui & E. Timdal	O-L-203051	OLICH3289-16	MK812322	
*Brianaria lutulata*	Lecanorales	NO, Aust-Agder, 2015, J.T. Klepsland	O-L-206489	OLICH3390-17	MK812659	
*Brianaria tuberculata*	Lecanorales	NO, Aust-Agder, 2015, J.T. Klepsland	O-L-206488	OLICH3389-17	MK811824	
*Brodoa atrofusca*	Lecanorales	NO, Buskerud, 2015, S. Rui & E. Timdal	O-L-200891	OLICH2544-16	MK811833	
*Brodoa atrofusca*	Lecanorales	NO, Oppland, 2013, M. Bendiksby et al.	O-L-184308	OLICH725-13	MK811829	atranorin (major), physodic acid (minor), protocetraric acid (major)
*Brodoa intestiniformis*	Lecanorales	NO, Oppland, 2013, M. Bendiksby et al.	O-L-190610	OLICH1474-14	MK811819	
*Brodoa intestiniformis*	Lecanorales	NO, Hordaland, 2014, E. Timdal	O-L-195806	OLICH1678-14	MK811842	
*Brodoa intestiniformis*	Lecanorales	NO, Telemark, 2014, E. Timdal	O-L-195819	OLICH2191-15	MK812115	
*Brodoa intestiniformis*	Lecanorales	NO, Buskerud, 2013, S. Rui & E. Timdal	O-L-184709	OLICH928-13	MK812552	
*Brodoa oroarctica*	Lecanorales	NO, Buskerud, 2015, S. Rui & E. Timdal	O-L-200939	OLICH2660-16	MK811789	
*Brodoa oroarctica*	Lecanorales	NO, Hedmark, 2017, E. Timdal	O-L-207794	OLICH4155-17	MK812012	
*Brodoa oroarctica*	Lecanorales	NO, Oppland, 2013, M. Bendiksby et al.	O-L-184297	OLICH726-13	MK812600	
*Bryobilimbia diapensiae*	Lecideales	NO, Finnmark, 2011, E. Timdal	O-L-170679	OLICH1167-14	MK812130	
*Bryobilimbia diapensiae*	Lecideales	NO, Nordland, 2012, R. Haugan	O-L-182225	OLICH1168-14	MK811983	
*Bryobilimbia diapensiae*	Lecideales	NO, Sør-Trøndelag, 2004, R. Haugan	O-L-142025	OLICH144-11	MK812681	
*Bryobilimbia diapensiae*	Lecideales	NO, Troms, 2003, R. Haugan	O-L-141675	OLICH145-11	MK812677	
*Bryobilimbia diapensiae*	Lecideales	NO, Finnmark, 2014, S. Rui	O-L-195960	OLICH1805-14	KY266915	
*Bryobilimbia fissuriseda*	Lecideales	NO, Oppland, 2005, E. Timdal	O-L-148945	OLICH627-13	MK811695	
*Bryobilimbia fissuriseda*	Lecideales	NO, Oppland, 2006, R. Haugan	O-L-145176	OLICH628-13	MK812621	
*Bryobilimbia fissuriseda*	Lecideales	NO, Oppland, 2013, M. Bendiksby et al.	O-L-184390	OLICH647-13	MK811896	
*Bryobilimbia hypnorum*	Lecideales	NO, Nordland, 2014, J.T. Klepsland	O-L-200331	OLICH3196-16	MK812370	
*Bryobilimbia hypnorum*	Lecideales	NO, Aust-Agder, 2014, J.T. Klepsland	O-L-200584	OLICH3198-16	MK811910	
*Bryocaulon divergens*	Lecanorales	NO, Hedmark, 2013, R. Haugan	O-L-190313	OLICH1472-14	MK812080	
*Bryocaulon divergens*	Lecanorales	NO, Finnmark, 2014, E. Timdal	O-L-195982	OLICH1827-14	KY266917	
*Bryocaulon divergens*	Lecanorales	NO, Sør-Trøndelag, 2014, E. Timdal	O-L-196360	OLICH1919-15	MK812069	
*Bryocaulon divergens*	Lecanorales	NO, Buskerud, 2014, S. Rui & E. Timdal	O-L-196317	OLICH2247-15	MK811658	
*Bryoplaca sinapisperma*	Teloschistales	NO, Troms, 2014, J.T. Klepsland	O-L-200396	OLICH4055-17	MK812160	
*Bryoria bicolor*	Lecanorales	NO, Buskerud, 2009, S. Svantesson	O-L-157404	OLICH1369-14	MK811780	
*Bryoria bicolor*	Lecanorales	NO, Rogaland, 2011, J.T. Klepsland	O-L-176874	OLICH1396-14	MK812469	
*Bryoria bicolor*	Lecanorales	NO, Hedmark, 2012, R. Haugan	O-L-179764	OLICH1400-14	MK811677	
*Bryoria bicolor*	Lecanorales	NO, Oppland, 2011, J.T. Klepsland	O-L-183745	OLICH1403-14	MK812128	
*Bryoria bicolor*	Lecanorales	NO, Hedmark, 2012, R. Haugan	O-L-183997	OLICH1404-14	MK812174	
*Bryoria capillaris*	Lecanorales	NO, Oslo, 2014, E. Timdal	O-L-199996	OLICH1970-15	MK812045	alectorialic acid, barbatolic acid
*Bryoria capillaris*	Lecanorales	SE, Lule Lappmark, 2014, E. Timdal	O-L-195685	OLICH2008-15	MK812229	alectorialic acid, barbatolic acid
*Bryoria capillaris*	Lecanorales	NO, Telemark, 2014, E. Timdal	O-L-195816	OLICH2188-15	MK812443	alectorialic acid, barbatolic acid
*Bryoria capillaris*	Lecanorales	NO, Buskerud, 2013, S. Rui & E. Timdal	O-L-184687	OLICH871-13	MK812425	alectorialic acid, barbatolic acid, fumarprotocetraric acid
*Bryoria fremontii*	Lecanorales	FI, Perä-Pohjanmaa, 2014, E. Timdal	O-L-195488	OLICH1498-14	MK812707	
*Bryoria fremontii*	Lecanorales	SE, Härjedalen, 2014, E. Timdal	O-L-195688	OLICH2011-15	MK812076	
*Bryoria fremontii*	Lecanorales	NO, Hedmark, 2014, R. Haugan	O-L-207993	OLICH3830-17	MK812563	
*Bryoria furcellata*	Lecanorales	FI, Perä-Pohjanmaa, 2014, E. Timdal	O-L-195487	OLICH1497-14	MK812435	
*Bryoria furcellata*	Lecanorales	NO, Buskerud, 2014, E. Timdal	O-L-195834	OLICH2206-15	MK811793	
*Bryoria furcellata*	Lecanorales	NO, Sør-Trøndelag, 2014, J.T. Klepsland	O-L-200527	OLICH2618-16	MK812653	
*Bryoria furcellata*	Lecanorales	NO, Hedmark, 2014, R. Haugan	O-L-207994	OLICH3831-17	MK812397	
*Bryoria implexa*	Lecanorales	FI, Perä-Pohjanmaa, 2014, E. Timdal	O-L-195485	OLICH1495-14	MK812092	fumarprotocetraric acid
*Bryoria implexa*	Lecanorales	NO, Finnmark, 2014, E. Timdal	O-L-195887	OLICH1732-14	KY266878	no lichen substances
*Bryoria implexa*	Lecanorales	SE, Torne Lappmark, 2014, E. Timdal	O-L-195665	OLICH2004-15	MK812090	no lichen substances
*Bryoria implexa*	Lecanorales	SE, Lule Lappmark, 2014, E. Timdal	O-L-195686	OLICH2009-15	MK812159	no lichen substances
*Bryoria implexa*	Lecanorales	NO, Vest-Agder, 2013, J.T. Klepsland	O-L-197823	OLICH2087-15	MK812714	fumarprotocetraric acid
*Bryoria implexa*	Lecanorales	NO, Buskerud, 2014, S. Rui & E. Timdal	O-L-196325	OLICH2255-15	MK812347	fumarprotocetraric acid
*Bryoria implexa*	Lecanorales	NO, Sør-Trøndelag, 2014, J.T. Klepsland	O-L-200539	OLICH2604-16	MK812375	fumarprotocetraric acid
*Bryoria implexa*	Lecanorales	NO, Hedmark, 2017, E. Timdal	O-L-207753	OLICH4156-17	MK811675	no lichen substances
*Bryoria implexa*	Lecanorales	NO, Buskerud, 2013, S. Rui & E. Timdal	O-L-184688	OLICH872-13	MK812231	fumarprotocetraric acid
*Bryoria nadvornikiana*	Lecanorales	NO, Sogn og Fjordane, 2008, G. Gaarder	O-L-155318	OLICH1367-14	MK812079	
*Bryoria nadvornikiana*	Lecanorales	NO, Hedmark, 2012, R. Haugan	O-L-183994	OLICH2172-15	MK811678	
*Bryoria nadvornikiana*	Lecanorales	NO, Oppland, 2011, J.T. Klepsland	O-L-176834	OLICH2173-15	MK812465	
*Bryoria nadvornikiana*	Lecanorales	NO, Sør-Trøndelag, 2014, R. Haugan	O-L-198885	OLICH2397-16	MK812326	
*Bryoria nadvornikiana*	Lecanorales	NO, Hedmark, 2017, E. Timdal	O-L-207754	OLICH4157-17	MK812417	alectorialic acid (major), barbatolic acid (minor), fumarprotocetraric acid (minor)
*Bryoria nadvornikiana*	Lecanorales	NO, Buskerud, 2013, S. Rui & E. Timdal	O-L-184689	OLICH873-13	MK812390	
*Bryoria nitidula*	Lecanorales	NO, Finnmark, 2014, E. Timdal	O-L-195715	OLICH1587-14	KY266934	fumarprotocetraric acid (major), protocetraric acid (minor)
*Bryoria simplicior*	Lecanorales	FI, Inarin Lappi, 2014, E. Timdal	O-L-195506	OLICH1505-14	MK812146	
*Bryoria simplicior*	Lecanorales	NO, Buskerud, 2014, E. Timdal	O-L-195825	OLICH2197-15	MK812415	
*Bryoria simplicior*	Lecanorales	NO, Telemark, 2015, S. Rui & E. Timdal	O-L-200936	OLICH2562-16	MK811887	
*Bryoria smithii*	Lecanorales	NO, Vest-Agder, 2011, J.T. Klepsland	O-L-177044	OLICH2165-15	MK811714	
*Bryoria smithii*	Lecanorales	NO, Rogaland, 2011, J.T. Klepsland	O-L-176881	OLICH2166-15	MK812250	
*Bryoria tenuis*	Lecanorales	NO, Sør-Trøndelag, 2014, E. Timdal	O-L-196345	OLICH1905-15	MK812591	
*Buellia arnoldii*	Caliciales	NO, Aust-Agder, 2015, H. Holien	TRH-L-16501	OLICH3670-17	MK811634	
*Buellia badia*	Caliciales	NO, Vestfold, 2015, E. Timdal	O-L-198941	OLICH2498-16	MK812426	
*Buellia disciformis*	Caliciales	NO, Troms, 2014, J.T. Klepsland	O-L-200381	OLICH4052-17	MK812587	
*Buellia dives*	Caliciales	NO, Nord-Trøndelag, 2015, H. Holien	TRH-L-16419	OLICH3631-17	MK811893	
*Buellia epigaea*	Caliciales	NO, Finnmark, 2014, E. Timdal	O-L-195523	OLICH1507-14	KY266900	
*Buellia erubescens*	Caliciales	NO, Nord-Trøndelag, 2015, U. Nordin	O-L-201275	OLICH3229-16	MK811955	
*Bunodophoron melanocarpum*	Lecanorales	NO, Rogaland, 2011, H. Fjeldstad	O-L-177411	OLICH1399-14	MK812059	
*Bunodophoron melanocarpum*	Lecanorales	NO, Hordaland, 2011, J.T. Klepsland	O-L-177097	OLICH2161-15	MK812620	
*Bunodophoron melanocarpum*	Lecanorales	NO, Sogn og Fjordane, 2010, J.T. Klepsland	O-L-175004	OLICH2163-15	MK811949	
*Calicium adspersum*	Caliciales	SE, Södermanland, 2015, S. Kistenich & E. Timdal	O-L-200822	OLICH2410-16	MK812068	
*Calicium pinicola*	Caliciales	NO, Oppland, 2015, R. Haugan	O-L-204191	OLICH4087-17	MK811674	
*Calicium viride*	Caliciales	NO, Hedmark, 2017, S. Rui & E. Timdal	O-L-207800	OLICH4161-17	MK812371	
*Callome multipartita*	Peltigerales	NO, Buskerud, 2013, S. Reiso & T. Høitomt	O-L-194256	OLICH2054-15	MK811803	
*Calogaya biatorina*	Teloschistales	NO, Sogn og Fjordane, 2013, J.T. Klepsland	O-L-197877	OLICH2459-16	MK812288	
*Calogaya decipiens*	Teloschistales	NO, Oslo, 2014, R. Haugan et al.	O-L-196061	OLICH1877-14	MK812143	
*Calogaya decipiens*	Teloschistales	NO, Hedmark, 1998, R. Haugan	O-L-55723	OLICH4389-17	MK812258	
*Calogaya decipiens*	Teloschistales	NO, Akershus, 2010, R. Haugan	O-L-167011	OLICH4390-17	MK811994	
*Caloplaca chlorina*	Teloschistales	NO, Vest-Agder, 2013, J.T. Klepsland	O-L-198079	OLICH4423-17	MK811786	
*Caloplaca chlorina*	Teloschistales	NO, Telemark, 2014, J.T. Klepsland	O-L-200720	OLICH4425-17	MK811988	
*Caloplaca demissa*	Teloschistales	NO, Rogaland, 2010, J.T. Klepsland	O-L-200085	OLICH3270-16	MK812156	
*Caloplaca demissa*	Teloschistales	NO, Sogn og Fjordane, 2010, S. Vatne	O-L-169301	OLICH3953-17	MK812032	
*Caloplaca monacensis*	Teloschistales	NO, Buskerud, 1998, H. Bratli	O-L-39356	OLICH4422-17	MK811632	
*Calvitimela armeniaca*	Lecanorales	NO, Finnmark, 2014, E. Timdal	O-L-195741	OLICH1613-14	KY266899	alectorialic acid
*Calvitimela livida*	Lecanorales	NO, Finnmark, 2014, E. Timdal	O-L-195842	OLICH1687-14	KY266947	atranorin, stictic acid
*Calvitimela melaleuca*	Lecanorales	NO, Finnmark, 2014, R. Haugan	O-L-195711	OLICH1583-14	KY266838	alectorialic acid, atranorin
*Calvitimela melaleuca*	Lecanorales	NO, Finnmark, 2014, E. Timdal	O-L-195915	OLICH1760-14	KY266897	alectorialic acid, norstictic acid
*Candelaria concolor*	Candelariales	NO, Rogaland, 2015, T. Tønsberg & E. Nygaard	BG-L-98168	OLICH3699-17	MK812350	
*Candelariella arctica*	Candelariales	NO, Finnmark, 2014, J.T. Klepsland	O-L-200509	OLICH3171-16	MK812015	
*Candelariella aurella*	Candelariales	NO, Troms, 1998, R. Armstrong	TROM_L_50030	OLICH3532-17	MK812178	
*Candelariella coralliza*	Candelariales	NO, Akershus, 2014, E. Timdal	O-L-199992	OLICH1966-15	MK812334	
*Candelariella coralliza*	Candelariales	NO, Buskerud, 2014, E. Timdal	O-L-195833	OLICH2205-15	MK812024	
*Candelariella coralliza*	Candelariales	NO, Aust-Agder, 2004, J.T. Klepsland	O-L-186001	OLICH2460-16	MK811884	
*Candelariella coralliza*	Candelariales	NO, Telemark, 2015, E. Timdal & S. Rui	O-L-200875	OLICH3836-17	MK812646	
*Candelariella placodizans*	Candelariales	NO, Troms, 2003, J.W. Bjerke	TROM_L_521176	OLICH3583-17	MK812262	
*Candelariella placodizans*	Candelariales	NO, Troms, 2009, J.T. Klepsland	O-L-164896	OLICH3949-17	MK811687	
*Candelariella placodizans*	Candelariales	NO, Nordland, 2000, E. Timdal	O-L-51849	OLICH3950-17	MK811709	
*Candelariella placodizans*	Candelariales	NO, Oppland, 2009, R. Haugan	O-L-159822	OLICH3951-17	MK812369	
*Candelariella placodizans*	Candelariales	NO, Telemark, 2008, J.T. Klepsland	O-L-164600	OLICH3952-17	MK812523	
*Candelariella vitellina*	Candelariales	NO, Nord-Trøndelag, 1999, H. Bratli	O-L-41044	OLICH3941-17	MK811713	
*Candelariella vitellina*	Candelariales	NO, Vest-Agder, 2011, H. Bratli	O-L-175901	OLICH3943-17	MK811697	
*Candelariella vitellina*	Candelariales	NO, Oslo, 2010, R. Haugan	O-L-166987	OLICH3948-17	MK812582	
*Cetraria aculeata*	Lecanorales	NO, Finnmark, 2014, M. Bendiksby	O-L-195862	OLICH1707-14	KY266883	
*Cetraria aculeata*	Lecanorales	NO, Buskerud, 2014, S. Rui & E. Timdal	O-L-196315	OLICH2245-15	MK811967	
*Cetraria aculeata*	Lecanorales	NO, Sør-Trøndelag, 2014, R. Haugan	O-L-196032	OLICH3808-17	MK811881	
*Cetraria aculeata*	Lecanorales	NO, Østfold, 2013, K.A. Lye	O-L-194757	OLICH4090-17	MK811694	
*Cetraria aculeata*	Lecanorales	NO, Vest-Agder, 2011, H. Bratli	O-L-175907	OLICH4091-17	MK811732	
*Cetraria ericetorum*	Lecanorales	NO, Hedmark, 2013, R. Haugan	O-L-190276	OLICH1469-14	MK811667	
*Cetraria ericetorum*	Lecanorales	NO, Finnmark, 2014, E. Timdal	O-L-195750	OLICH1622-14	KY266868	
*Cetraria ericetorum*	Lecanorales	NO, Buskerud, 2015, E. Timdal & S. Rui	O-L-200904	OLICH3839-17	MK811635	
*Cetraria ericetorum*	Lecanorales	NO, Buskerud, 2013, S. Rui & E. Timdal	O-L-184702	OLICH884-13	MK811886	
*Cetraria islandica*	Lecanorales	NO, Oslo, 2014, E. Timdal	O-L-200003	OLICH1977-15	MK812195	
*Cetraria islandica*	Lecanorales	NO, Telemark, 2014, E. Timdal	O-L-195818	OLICH2190-15	MK812355	
*Cetraria islandica*	Lecanorales	NO, Akershus, 2014, E. Timdal	O-L-196304	OLICH2234-15	MK812550	
*Cetraria islandica*	Lecanorales	NO, Sør-Trøndelag, 2017, E. Timdal	O-L-208279	OLICH4164-17	MK812647	
*Cetraria islandica*	Lecanorales	NO, Sør-Trøndelag, 2017, E. Timdal	O-L-208278	OLICH4165-17	MK812026	
*Cetraria islandica*	Lecanorales	NO, Buskerud, 2013, S. Rui & E. Timdal	O-L-184703	OLICH885-13	MK812121	
*Cetraria muricata*	Lecanorales	NO, Hordaland, 2014, E. Timdal	O-L-195799	OLICH1671-14	MK812154	
*Cetraria muricata*	Lecanorales	NO, Telemark, 2014, E. Timdal	O-L-195815	OLICH2187-15	MK812716	
*Cetraria nigricans*	Lecanorales	NO, Troms, 2012, J.T. Klepsland	O-L-186197	OLICH1436-14	MK812225	
*Cetraria nigricans*	Lecanorales	NO, Finnmark, 2011, E. Timdal	O-L-170662	OLICH2159-15	MK812613	
*Cetraria odontella*	Lecanorales	NO, Buskerud, 2012, T.H. Hofton	O-L-194457	OLICH1485-14	MK812629	
*Cetraria sepincola*	Lecanorales	NO, Hedmark, 2013, R. Haugan	O-L-190020	OLICH1463-14	MK812418	
*Cetraria sepincola*	Lecanorales	NO, Telemark, 2015, S. Rui & E. Timdal	O-L-200881	OLICH2538-16	MK812133	
*Cetraria sepincola*	Lecanorales	NO, Buskerud, 2013, S. Rui & E. Timdal	O-L-184705	OLICH886-13	MK811790	
*Cetrariella commixta*	Lecanorales	NO, Finnmark, 2014, M. Westberg	O-L-195926	OLICH1771-14	KY266843	
*Cetrariella delisei*	Lecanorales	NO, Oppland, 2013, R. Haugan	O-L-190044	OLICH1465-14	MK812542	
*Cetrariella delisei*	Lecanorales	NO, Finnmark, 2014, E. Timdal	O-L-195559	OLICH1517-14	KY266903	
*Cetrariella delisei*	Lecanorales	NO, Finnmark, 2014, H. Holien	O-L-195763	OLICH1635-14	KY266858	
*Cetrariella delisei*	Lecanorales	NO, Finnmark, 2014, M. Westberg	O-L-195882	OLICH1727-14	KY266955	
*Cetrariella delisei*	Lecanorales	NO, Finnmark, 2014, H. Holien	O-L-195946	OLICH1791-14	KY266892	
*Cetrariella delisei*	Lecanorales	NO, Buskerud, 2015, S. Rui & E. Timdal	O-L-200893	OLICH2546-16	MK811762	
*Cetrariella fastigiata*	Lecanorales	NO, Finnmark, 2014, E. Timdal	O-L-195560	OLICH1518-14	KY266875	
*Cetrariella fastigiata*	Lecanorales	NO, Finnmark, 2014, H. Holien	O-L-195762	OLICH1634-14	KY266870	
*Cetrariella fastigiata*	Lecanorales	NO, Finnmark, 2014, R. Haugan	O-L-195985	OLICH1830-14	KY266890	
*Cetrariella fastigiata*	Lecanorales	NO, Finnmark, 2011, E. Timdal	O-L-170481	OLICH2154-15	MK811637	
*Cetrariella fastigiata*	Lecanorales	NO, Troms, 2011, J.T. Klepsland	O-L-177156	OLICH2155-15	MK812493	
*Cetrelia cetrarioides*	Lecanorales	NO, Telemark, 2005, S. Reiso	O-L-141329	OLICH1354-14	MK811823	anziaic acid (minor), atranorin (major), perlatolic acid (major), 4-O-methylolivetoric acid (minor)
*Cetrelia cetrarioides*	Lecanorales	NO, Oppland, 2011, J.T. Klepsland	O-L-176836	OLICH1395-14	MK812086	anziaic acid (minor), atranorin (major), perlatolic acid (major), 4-O-methylolivetoric acid (minor)
*Cetrelia cetrarioides*	Lecanorales	NO, Sogn og Fjordane, 2009, T.H. Hofton	O-L-186891	OLICH1453-14	MK812180	anziaic acid (minor), atranorin (major), perlatolic acid (major), 4-O-methylolivetoric acid (minor)
*Cetrelia cetrarioides*	Lecanorales	NO, Buskerud, 2014, S. Rui & E. Timdal	O-L-200022	OLICH2470-16	MK812062	anziaic acid (minor), atranorin (major), perlatolic acid (major), 4-O-methylolivetoric acid (minor)
*Cetrelia cetrarioides*	Lecanorales	NO, Oppland, 2015, E. Timdal	O-L-201355	OLICH2740-16	MK812302	anziaic acid (minor), atranorin (major), perlatolic acid (major), 4-O-methylolivetoric acid (minor)
*Cetrelia cetrarioides*	Lecanorales	NO, Buskerud, 2016, M. Bendiksby & E. Timdal	O-L-208029	OLICH4257-17	MK812008	anziaic acid (minor), atranorin (major), perlatolic acid (major), 4-O-methylolivetoric acid (minor)
*Cetrelia monachorum*	Lecanorales	NO, Buskerud, 2012, T.H. Hofton	O-L-194448	OLICH4095-17	MK811729	anziaic acid (minor), atranorin (major), imbricaric acid (major), 4-O-denmethylimbricaric acid (minor)
*Chaenotheca chlorella*	Coniocybales	NO, Sør-Trøndelag, 2014, S. Vatne	O-L-200765	OLICH2447-16	MK812013	
*Chaenotheca ferruginea*	Coniocybales	SE, Södermanland, 2015, S. Kistenich & E. Timdal	O-L-200838	OLICH2426-16	MK812287	
*Chaenotheca gracilenta*	Coniocybales	NO, Nord-Trøndelag, 2015, M. Bendiksby et al.	O-L-201318	OLICH2790-16	MK812452	
*Chaenotheca gracillima*	Coniocybales	NO, Sør-Trøndelag, 2014, S. Vatne	O-L-200773	OLICH2446-16	MK812119	
*Cladonia albonigra*	Lecanorales	NO, Troms, 2014, E. Timdal	O-L-195659	OLICH1999-15	MK812147	
*Cladonia amaurocraea*	Lecanorales	NO, Troms, 2014, E. Timdal	O-L-195776	OLICH1648-14	MK811717	
*Cladonia amaurocraea*	Lecanorales	NO, Finnmark, 2014, H. Holien	O-L-195877	OLICH1722-14	KY266961	
*Cladonia amaurocraea*	Lecanorales	NO, Sør-Trøndelag, 2014, E. Timdal	O-L-196364	OLICH1923-15	MK812471	
*Cladonia amaurocraea*	Lecanorales	NO, Buskerud, 2014, S. Rui & E. Timdal	O-L-196316	OLICH2246-15	MK811885	
*Cladonia arbuscula*	Lecanorales	NO, Finnmark, 2014, A. Kosuthova	O-L-195866	OLICH1711-14	KY266933	
*Cladonia arbuscula*	Lecanorales	NO, Sør-Trøndelag, 2014, E. Timdal	O-L-196358	OLICH1917-15	MK812466	
*Cladonia arbuscula*	Lecanorales	NO, Telemark, 2014, E. Timdal	O-L-196268	OLICH1943-15	MK811810	
*Cladonia arbuscula*	Lecanorales	NO, Akershus, 2014, E. Timdal	O-L-196301	OLICH2231-15	MK812036	
*Cladonia arbuscula*	Lecanorales	NO, Vestfold, 2015, E. Timdal	O-L-198942	OLICH2497-16	MK811763	
*Cladonia bellidiflora*	Lecanorales	NO, Finnmark, 2014, H. Holien	O-L-195740	OLICH1612-14	KY266902	
*Cladonia bellidiflora*	Lecanorales	NO, Finnmark, 2014, R. Haugan	O-L-195881	OLICH1726-14	KY266913	
*Cladonia bellidiflora*	Lecanorales	NO, Finnmark, 2014, A. Kosuthova	O-L-195906	OLICH1751-14	KY266845	
*Cladonia bellidiflora*	Lecanorales	NO, Finnmark, 2014, E. Timdal	O-L-195958	OLICH1803-14	KY266921	
*Cladonia caespiticia*	Lecanorales	NO, Østfold, 2012, J.T. Klepsland	O-L-186036	OLICH1420-14	MK811962	
*Cladonia caespiticia*	Lecanorales	NO, Telemark, 2015, S. Rui & E. Timdal	O-L-200916	OLICH2576-16	MK812605	
*Cladonia caespiticia*	Lecanorales	NO, Hordaland, 2011, J.T. Klepsland	O-L-183605	OLICH4081-17	MK811712	
*Cladonia cariosa*	Lecanorales	NO, Buskerud, 2012, E.W. Hanssen et al.	O-L-186370	OLICH1443-14	MK811826	
*Cladonia cariosa*	Lecanorales	NO, Oppland, 2013, M. Bendiksby et al.	O-L-184343	OLICH743-13	MK812622	
*Cladonia carneola*	Lecanorales	NO, Finnmark, 2014, A. Kosuthova	O-L-195963	OLICH1808-14	KY266855	
*Cladonia cenotea*	Lecanorales	NO, Akershus, 2014, E. Timdal	O-L-196307	OLICH2237-15	MK811749	
*Cladonia cenotea*	Lecanorales	NO, Hedmark, 2014, R. Haugan	O-L-198892	OLICH2393-16	MK812468	
*Cladonia cenotea*	Lecanorales	NO, Hedmark, 2014, R. Haugan	O-L-207986	OLICH3824-17	MK812541	
*Cladonia cenotea*	Lecanorales	NO, Nord-Trøndelag, 2015, R. Haugan	O-L-201300	OLICH3855-17	MK812267	
*Cladonia cenotea*	Lecanorales	NO, Buskerud, 2013, S. Rui & E. Timdal	O-L-184714	OLICH932-13	MK812699	
*Cladonia chlorophaea*	Lecanorales	NO, Buskerud, 2016, S. Rui & E. Timdal	O-L-208034	OLICH4262-17	MK812242	
*Cladonia coccifera*	Lecanorales	NO, Finnmark, 2014, H. Holien	O-L-195895	OLICH1740-14	KY266935	
*Cladonia coniocraea*	Lecanorales	NO, Akershus, 2014, E. Timdal	O-L-196310	OLICH2240-15	MK812094	
*Cladonia coniocraea*	Lecanorales	NO, Telemark, 2015, S. Rui & E. Timdal	O-L-200908	OLICH2569-16	MK812138	
*Cladonia cornuta*	Lecanorales	NO, Akershus, 2014, E. Timdal	O-L-196260	OLICH2267-15	MK812497	
*Cladonia cornuta*	Lecanorales	NO, Hedmark, 2015, E. Timdal	O-L-200857	OLICH2508-16	MK812414	
*Cladonia cornuta*	Lecanorales	NO, Hedmark, 2016, E. Timdal	O-L-201516	OLICH3979-17	MK811731	
*Cladonia cornuta*	Lecanorales	NO, Telemark, 2017, E. Timdal	O-L-208059	OLICH4167-17	MK812047	
*Cladonia cornuta*	Lecanorales	NO, Buskerud, 2013, S. Rui & E. Timdal	O-L-184715	OLICH933-13	MK812277	
*Cladonia crispata*	Lecanorales	NO, Finnmark, 2014, R. Haugan	O-L-195880	OLICH1725-14	KY266865	
*Cladonia crispata*	Lecanorales	NO, Buskerud, 2014, E. Timdal	O-L-195824	OLICH2196-15	MK811863	
*Cladonia crispata*	Lecanorales	NO, Telemark, 2016, S. Rui & E. Timdal	O-L-205005	OLICH3995-17	MK811728	
*Cladonia crispata*	Lecanorales	NO, Vest-Agder, 2016, S. Rui & E. Timdal	O-L-204984	OLICH3996-17	MK812514	
*Cladonia crispata*	Lecanorales	NO, Telemark, 2017, E. Timdal	O-L-208058	OLICH4275-17	MK811707	
*Cladonia cyanipes*	Lecanorales	NO, Finnmark, 2014, H. Holien	O-L-195758	OLICH1630-14	KY266889	
*Cladonia cyanipes*	Lecanorales	NO, Finnmark, 2014, E. Timdal	O-L-195981	OLICH1826-14	KY266977	
*Cladonia cyathomorpha*	Lecanorales	NO, Vest-Agder, 2012, J.T. Klepsland	O-L-186087	OLICH1430-14	MK811836	
*Cladonia cyathomorpha*	Lecanorales	NO, Aust-Agder, 2010, J.T. Klepsland	O-L-175272	OLICH2147-15	MK811648	
*Cladonia decorticata*	Lecanorales	SE, Torne Lappmark, 2014, E. Timdal	O-L-195679	OLICH2006-15	MK811738	
*Cladonia ecmocyna*	Lecanorales	NO, Hordaland, 2014, E. Timdal	O-L-195793	OLICH1665-14	MK812344	
*Cladonia ecmocyna*	Lecanorales	NO, Finnmark, 2014, H. Holien	O-L-195956	OLICH1801-14	KY266972	
*Cladonia ecmocyna*	Lecanorales	NO, Telemark, 2015, S. Rui & E. Timdal	O-L-200933	OLICH2559-16	MK812085	
*Cladonia fimbriata*	Lecanorales	NO, Finnmark, 2014, E. Timdal	O-L-195743	OLICH1615-14	KY266953	
*Cladonia fimbriata*	Lecanorales	NO, Vest-Agder, 2011, J.T. Klepsland	O-L-183619	OLICH2145-15	MK812488	fumarprotocetraric acid
*Cladonia fimbriata*	Lecanorales	NO, Telemark, 2015, S. Rui & E. Timdal	O-L-200909	OLICH2570-16	MK811629	
*Cladonia furcata*	Lecanorales	NO, Buskerud, 2011, K.A. Lye & T. Høitomt	O-L-194749	OLICH1492-14	MK811985	
*Cladonia furcata*	Lecanorales	NO, Finnmark, 2014, T. Tønsberg	O-L-195964	OLICH1809-14	KY266930	
*Cladonia furcata*	Lecanorales	NO, Vestfold, 2014, S. Rui & E. Timdal	O-L-196298	OLICH2228-15	MK812624	
*Cladonia furcata*	Lecanorales	NO, Akershus, 2014, E. Timdal	O-L-196308	OLICH2238-15	MK811830	
*Cladonia furcata*	Lecanorales	NO, Nord-Trøndelag, 2015, R. Haugan	O-L-201273	OLICH3847-17	MK812251	
*Cladonia gracilis*	Lecanorales	NO, Finnmark, 2014, E. Timdal	O-L-195764	OLICH1636-14	KY266906	
*Cladonia gracilis*	Lecanorales	NO, Sør-Trøndelag, 2014, E. Timdal	O-L-196359	OLICH1918-15	MK812362	
*Cladonia gracilis*	Lecanorales	NO, Buskerud, 2014, S. Rui & E. Timdal	O-L-196321	OLICH2251-15	MK811813	
*Cladonia gracilis*	Lecanorales	NO, Akershus, 2014, E. Timdal	O-L-196262	OLICH2269-15	MK811638	
*Cladonia gracilis*	Lecanorales	NO, Sør-Trøndelag, 2014, R. Haugan	O-L-196047	OLICH3802-17	MK812342	
*Cladonia grayi*	Lecanorales	NO, Finnmark, 2014, H. Holien	O-L-195757	OLICH1629-14	KY266978	
*Cladonia krogiana*	Lecanorales	NO, Telemark, 2003, B.P. Løfall & A. Ognedal	O-L-127158	OLICH1337-14	MK811907	barbatic acid, chlorovinetorin
*Cladonia krogiana*	Lecanorales	NO, Akershus, 2004, B.P. Løfall	O-L-129021	OLICH1339-14	MK811663	barbatic acid, chlorovinetorin
*Cladonia krogiana*	Lecanorales	NO, Akershus, 2012, J.T. Klepsland	O-L-186068	OLICH2136-15	MK812057	
*Cladonia macrophylla*	Lecanorales	NO, Oslo, 2014, E. Timdal	O-L-200002	OLICH1976-15	MK811782	
*Cladonia macrophylla*	Lecanorales	NO, Buskerud, 2014, S. Rui & E. Timdal	O-L-200021	OLICH2469-16	MK812683	
*Cladonia macrophyllodes*	Lecanorales	NO, Sør-Trøndelag, 2014, E. Timdal	O-L-196379	OLICH1938-15	MK812614	
*Cladonia maxima*	Lecanorales	NO, Buskerud, 2014, E. Timdal	O-L-195829	OLICH2201-15	MK811693	
*Cladonia maxima*	Lecanorales	NO, Oppland, 2013, M. Bendiksby et al.	O-L-184361	OLICH692-13	MK812518	
*Cladonia mitis*	Lecanorales	NO, Buskerud, 2013, S. Rui & E. Timdal	O-L-184706	OLICH887-13	MK811894	
*Cladonia parasitica*	Lecanorales	NO, Hedmark, 2014, R. Haugan	O-L-207988	OLICH3825-17	MK811639	
*Cladonia parasitica*	Lecanorales	NO, Hedmark, 2016, E. Timdal	O-L-201525	OLICH3989-17	MK812470	
*Cladonia phyllophora*	Lecanorales	NO, Sør-Trøndelag, 2014, E. Timdal	O-L-196375	OLICH1934-15	MK812644	
*Cladonia phyllophora*	Lecanorales	NO, Buskerud, 2014, S. Rui & E. Timdal	O-L-196320	OLICH2250-15	MK812551	
*Cladonia pleurota*	Lecanorales	NO, Finnmark, 2014, E. Timdal	O-L-195959	OLICH1804-14	KY266968	usnic acid, zeorin
*Cladonia pulvinata*	Lecanorales	NO, Sør-Trøndelag, 2015, H. Holien	TRH-L-16327	OLICH2920-16	MK811846	fumarprotocetraric acid, psoromic acid
*Cladonia pyxidata*	Lecanorales	NO, Østfold, 2012, J.T. Klepsland	O-L-186049	OLICH1425-14	MK812571	fumarprotocetraric acid
*Cladonia pyxidata*	Lecanorales	NO, Finnmark, 2014, E. Timdal	O-L-195914	OLICH1759-14	KY266877	fumarprotocetraric acid (major), protocetraric acid (minor)
*Cladonia pyxidata*	Lecanorales	NO, Oslo, 2014, S. Rui & E. Timdal	O-L-196073	OLICH1889-14	MK812676	
*Cladonia rangiferina*	Lecanorales	NO, Finnmark, 2014, E. Timdal	O-L-195755	OLICH1627-14	KY266884	
*Cladonia rangiferina*	Lecanorales	NO, Troms, 2014, E. Timdal	O-L-195774	OLICH1646-14	MK811970	
*Cladonia rangiferina*	Lecanorales	NO, Akershus, 2014, E. Timdal	O-L-196302	OLICH2232-15	MK812460	
*Cladonia rangiferina*	Lecanorales	NO, Sør-Trøndelag, 2014, R. Haugan	O-L-196037	OLICH3806-17	MK811708	
*Cladonia rangiferina*	Lecanorales	NO, Nord-Trøndelag, 2015, R. Haugan	O-L-201298	OLICH3854-17	MK812260	
*Cladonia rangiformis*	Lecanorales	NO, Telemark, 2012, S. Reiso	O-L-194209	OLICH2062-15	MK812118	atranorin
*Cladonia rangiformis*	Lecanorales	NO, Rogaland, 2013, J.T. Klepsland	O-L-198309	OLICH2351-15	MK811656	
*Cladonia rei*	Lecanorales	NO, Telemark, 2014, S. Rui & E. Timdal	O-L-194142	OLICH1479-14	MK811774	
*Cladonia rei*	Lecanorales	NO, Oslo, 2014, J.T. Klepsland	O-L-200262b	OLICH4086-17	MK812389	fumarprotocetraric acid, homosekikaic acid
*Cladonia squamosa*	Lecanorales	NO, Buskerud, 2013, S. Rui & E. Timdal	O-L-184733	OLICH1415-14	MK812303	
*Cladonia squamosa*	Lecanorales	NO, Troms, 2014, E. Timdal	O-L-195646	OLICH1558-14	MK811958	
*Cladonia squamosa*	Lecanorales	NO, Finnmark, 2014, H. Holien	O-L-195909	OLICH1754-14	KY266973	
*Cladonia squamosa*	Lecanorales	NO, Akershus, 2014, E. Timdal	O-L-196309	OLICH2239-15	MK812360	
*Cladonia stellaris*	Lecanorales	NO, Finnmark, 2014, A. Kosuthova	O-L-195905	OLICH1750-14	KY266887	
*Cladonia stellaris*	Lecanorales	NO, Akershus, 2014, E. Timdal	O-L-196263	OLICH2270-15	MK812506	
*Cladonia stellaris*	Lecanorales	NO, Buskerud, 2013, S. Rui & E. Timdal	O-L-184707	OLICH926-13	MK812280	
*Cladonia straminea*	Lecanorales	NO, Finnmark, 2014, E. Timdal	O-L-195976	OLICH1821-14	KY266880	
*Cladonia strepsilis*	Lecanorales	NO, Oslo, 2014, E. Timdal	O-L-200000	OLICH1974-15	MK812544	
*Cladonia strepsilis*	Lecanorales	NO, Østfold, 2017, S. Rui & E. Timdal	O-L-208340	OLICH4270-17	MK812494	
*Cladonia stygia*	Lecanorales	NO, Telemark, 2014, E. Timdal	O-L-196269	OLICH1944-15	MK812684	
*Cladonia stygia*	Lecanorales	NO, Akershus, 2014, E. Timdal	O-L-196303	OLICH2233-15	MK812337	
*Cladonia stygia*	Lecanorales	NO, Buskerud, 2013, S. Rui & E. Timdal	O-L-184708	OLICH927-13	MK812669	
*Cladonia subcervicornis*	Lecanorales	NO, Rogaland, 2013, S. Vatne	O-L-194122	OLICH2072-15	MK812018	
*Cladonia subcervicornis*	Lecanorales	NO, Sør-Trøndelag, 2014, R. Haugan	O-L-196028	OLICH3794-17	MK812661	
*Cladonia subcervicornis*	Lecanorales	NO, Sør-Trøndelag, 2014, R. Haugan	O-L-196053	OLICH3811-17	MK812155	
*Cladonia subcervicornis*	Lecanorales	NO, Rogaland, 2016, S. Rui & E. Timdal	O-L-204985	OLICH3998-17	MK811825	
*Cladonia subcervicornis*	Lecanorales	NO, Vest-Agder, 2011, J.T. Klepsland	O-L-176962	OLICH4113-17	MK812379	
*Cladonia subfurcata*	Lecanorales	NO, Finnmark, 2014, H. Holien	O-L-195987	OLICH1832-14	KY266923	
*Cladonia subfurcata*	Lecanorales	NO, Buskerud, 2014, S. Rui & E. Timdal	O-L-196319	OLICH2249-15	MK812649	
*Cladonia subfurcata*	Lecanorales	NO, Telemark, 2015, S. Rui & E. Timdal	O-L-200934	OLICH2560-16	MK812597	
*Cladonia subulata*	Lecanorales	NO, Sør-Trøndelag, 2013, R. Haugan	O-L-190281	OLICH1471-14	MK812581	
*Cladonia subulata*	Lecanorales	FI, Inarin Lappi, 2014, E. Timdal	O-L-195490	OLICH1500-14	MK811811	
*Cladonia subulata*	Lecanorales	NO, Akershus, 2014, E. Timdal	O-L-196299	OLICH2229-15	MK812279	
*Cladonia subulata*	Lecanorales	NO, Telemark, 2015, S. Rui & E. Timdal	O-L-200914	OLICH2574-16	MK811787	
*Cladonia sulphurina*	Lecanorales	NO, Finnmark, 2014, A. Kosuthova	O-L-195911	OLICH1756-14	KY266846	
*Cladonia symphycarpa*	Lecanorales	NO, Finnmark, 2014, E. Timdal	O-L-195597	OLICH1535-14	MK811971	atranorin
*Cladonia turgida*	Lecanorales	NO, Akershus, 2014, S. Rui & E. Timdal	O-L-199980	OLICH1954-15	MK811657	
*Cladonia turgida*	Lecanorales	NO, Telemark, 2015, S. Rui & E. Timdal	O-L-200932	OLICH2558-16	MK812027	
*Cladonia uncialis*	Lecanorales	NO, Østfold, 2013, R. Haugan	O-L-190182	OLICH1467-14	MK812589	
*Cladonia uncialis*	Lecanorales	NO, Finnmark, 2014, H. Holien	O-L-195939	OLICH1784-14	KY266974	
*Cladonia verticillata*	Lecanorales	NO, Hordaland, 2014, E. Timdal	O-L-195797	OLICH1669-14	MK812461	
*Cladonia verticillata*	Lecanorales	NO, Buskerud, 2014, S. Rui & E. Timdal	O-L-196312	OLICH2242-15	MK811806	
*Cladonia verticillata*	Lecanorales	NO, Sør-Trøndelag, 2015, H. Holien	TRH-L-16326	OLICH2921-16	MK812385	fumarprotocetraric acid
*Cladonia zopfii*	Lecanorales	NO, Sør-Trøndelag, 2014, R. Haugan	O-L-196044	OLICH3804-17	MK811805	
*Coccotrema citrinescens*	Pertusariales	NO, Nordland, 2015, T. Tønsberg & A. Botnen	BG-L-99172	OLICH3741-17	MK811814	
*Coccotrema citrinescens*	Pertusariales	NO, Nordland, 2015, T. Tønsberg	BG-L-98897	OLICH3754-17	MK811895	
*Coccotrema citrinescens*	Pertusariales	NO, Møre og Romsdal, 2015, T. Tønsberg & Z. Palice	BG-L-99069	OLICH3774-17	MK812329	
*Coccotrema citrinescens*	Pertusariales	NO, Nordland, 2013, J.T. Klepsland	O-L-198020	OLICH4212-17	MK812183	
*Coccotrema citrinescens*	Pertusariales	NO, Sør-Trøndelag, 2011, R. Haugan	O-L-173864	OLICH4403-17	MK812186	stictic acid
*Cornicularia normoerica*	Lecanorales	NO, Aust-Agder, 2014, J.T. Klepsland	O-L-200754	OLICH2603-16	MK812042	
*Cornicularia normoerica*	Lecanorales	NO, Nord-Trøndelag, 2015, R. Haugan	O-L-201280	OLICH3850-17	MK811980	
*Cystocoleus ebeneus*	Capnodiales	NO, Sør-Trøndelag, 1998, R. Haugan	O-L-55770	OLICH3942-17	MK811795	
*Dactylina ramulosa*	Lecanorales	NO, Troms, 2012, J.T. Klepsland	O-L-186199	OLICH1438-14	MK812632	
*Dendrographa latebrarum*	Arthoniales	NO, Møre og Romsdal, 2016, H. Holien	TRH-L-16972	OLICH3682-17	MK811843	
*Dermatocarpon bachmannii*	Verrucariales	NO, Østfold, 2000, B. P. Løfall et al.	O-L-78257	OLICH2297-15	MK812540	
*Dermatocarpon bachmannii*	Verrucariales	NO, Østfold, 2005, B. P. Løfall	O-L-147654	OLICH2305-15	MK812444	
*Dermatocarpon deminuens*	Verrucariales	NO, Østfold, 2000, B. P. Løfall	O-L-78264	OLICH2298-15	MK812502	
*Dermatocarpon deminuens*	Verrucariales	NO, Østfold, 1998, B. P. Løfall	O-L-36303	OLICH2299-15	MK812476	
*Dermatocarpon deminuens*	Verrucariales	NO, Østfold, 2000, B. P. Løfall et al.	O-L-78258	OLICH2304-15	MK812075	
*Dermatocarpon luridum*	Verrucariales	NO, Buskerud, 2009, T.H. Hofton	O-L-187021	OLICH1455-14	MK812236	
*Dermatocarpon meiophyllizium*	Verrucariales	NO, Østfold, 2006, B. P. Løfall	O-L-147725	OLICH2296-15	MK812273	
*Dermatocarpon meiophyllizium*	Verrucariales	NO, Hedmark, 2006, R. Haugan	O-L-145251	OLICH2300-15	MK812522	
*Dermatocarpon meiophyllizium*	Verrucariales	NO, Østfold, 2001, B. P. Løfall	O-L-105979	OLICH2306-15	MK811820	
*Dermatocarpon miniatum*	Verrucariales	NO, Sør-Trøndelag, 2014, R. Haugan	O-L-196009	OLICH1854-14	MK812245	
*Dibaeis baeomyces*	Pertusariales	NO, Buskerud, 2014, S. Rui & E. Timdal	O-L-200009	OLICH1983-15	MK812560	
*Dibaeis baeomyces*	Pertusariales	NO, Nord-Trøndelag, 2015, M. Bendiksby et al.	O-L-201296	OLICH2796-16	MK812219	
*Diploschistes scruposus*	Ostropales	NO, Finnmark, 2014, S. Fernandez	O-L-195913	OLICH1758-14	KY266920	
*Diploschistes scruposus*	Ostropales	NO, Aust-Agder, 2013, J.T. Klepsland	O-L-198319	OLICH2350-15	MK811671	
*Diplotomma alboatrum*	Caliciales	NO, Finnmark, 2014, E. Timdal	O-L-195577	OLICH1527-14	MK812220	
*Eopyrenula leucoplaca*	Vezdaeales	NO, Telemark, 2016, J.T. Klepsland	O-L-206771	OLICH3490-17	MK811948	
*Epilichen glauconigellus*	Rhizocarpales	NO, Oppland, 2012, S. Rui & E. Timdal	O-L-179907	OLICH1100-14	MK812566	
*Epilichen glauconigellus*	Rhizocarpales	NO, Troms, 2003, R. Haugan	O-L-141634	OLICH1101-14	MK811705	
*Epilichen scabrosus*	Rhizocarpales	NO, Hedmark, 2012, S. Rui & E. Timdal	O-L-179955	OLICH1102-14	MK812558	
*Epilichen scabrosus*	Rhizocarpales	NO, Troms, 2009, J.T. Klepsland	O-L-164936	OLICH832-13	MK812386	
*Evernia divaricata*	Lecanorales	NO, Buskerud, 2011, T.H. Hofton	O-L-194431	OLICH1484-14	MK812536	
*Evernia divaricata*	Lecanorales	NO, Oppland, 2012, T.H. Hofton	O-L-194526	OLICH2315-15	MK811891	
*Evernia mesomorpha*	Lecanorales	NO, Hedmark, 2012, T.H. Hofton	O-L-194511	OLICH1489-14	MK812555	
*Evernia mesomorpha*	Lecanorales	NO, Buskerud, 2014, S. Rui & E. Timdal	O-L-200008	OLICH1982-15	MK811689	
*Evernia mesomorpha*	Lecanorales	NO, Oppland, 2011, J.T. Klepsland	O-L-176825	OLICH2308-15	MK811679	
*Evernia mesomorpha*	Lecanorales	NO, Sør-Trøndelag, 2014, J.T. Klepsland	O-L-200526	OLICH2614-16	MK812464	
*Evernia prunastri*	Lecanorales	NO, Oslo, 2014, S. Rui & E. Timdal	O-L-196076	OLICH1892-14	MK811993	
*Evernia prunastri*	Lecanorales	NO, Akershus, 2014, E. Timdal	O-L-199988	OLICH1962-15	MK812247	
*Evernia prunastri*	Lecanorales	NO, Vestfold, 2014, S. Rui & E. Timdal	O-L-196292	OLICH2222-15	MK812058	
*Evernia prunastri*	Lecanorales	NO, Hedmark, 2015, E. Timdal	O-L-200856	OLICH2507-16	MK811838	
*Evernia prunastri*	Lecanorales	NO, Telemark, 2015, S. Rui & E. Timdal	O-L-200923	OLICH2531-16	MK811722	
*Flavocetraria cucullata*	Lecanorales	NO, Østfold, 2013, B.P. Løfall	O-L-187362	OLICH1459-14	MK812129	
*Flavocetraria cucullata*	Lecanorales	NO, Finnmark, 2014, S. Rui	O-L-195752	OLICH1624-14	KY266924	
*Flavocetraria cucullata*	Lecanorales	NO, Troms, 2014, E. Timdal	O-L-195778	OLICH1650-14	MK811878	
*Flavocetraria cucullata*	Lecanorales	NO, Sør-Trøndelag, 2014, E. Timdal	O-L-196338	OLICH2276-15	MK812660	
*Flavocetraria cucullata*	Lecanorales	NO, Svalbard, 2015, S. Rui	O-L-203288	OLICH2707-16	MK812458	
*Flavocetraria cucullata*	Lecanorales	NO, Buskerud, 2015, E. Timdal & S. Rui	O-L-200903	OLICH3838-17	MK812490	
*Flavocetraria cucullata*	Lecanorales	NO, Buskerud, 2013, S. Rui & E. Timdal	O-L-184721	OLICH938-13	MK812603	
*Flavocetraria nivalis*	Lecanorales	NO, Østfold, 2013, B.P. Løfall	O-L-187361	OLICH1458-14	MK812252	
*Flavocetraria nivalis*	Lecanorales	NO, Finnmark, 2014, S. Rui	O-L-195723	OLICH1595-14	KY266896	
*Flavocetraria nivalis*	Lecanorales	NO, Troms, 2014, E. Timdal	O-L-195777	OLICH1649-14	MK811972	
*Flavocetraria nivalis*	Lecanorales	NO, Buskerud, 2014, S. Rui & E. Timdal	O-L-196311	OLICH2241-15	MK811652	
*Flavocetraria nivalis*	Lecanorales	NO, Sør-Trøndelag, 2014, E. Timdal	O-L-196339	OLICH2277-15	MK812419	
*Flavocetraria nivalis*	Lecanorales	NO, Svalbard, 2015, S. Rui	O-L-203283	OLICH2702-16	MK811681	
*Flavocetraria nivalis*	Lecanorales	NO, Hedmark, 2014, R. Haugan	O-L-207989	OLICH3829-17	MK812064	
*Flavocetraria nivalis*	Lecanorales	NO, Buskerud, 2015, E. Timdal & S. Rui	O-L-200902	OLICH3837-17	MK812356	
*Flavoparmelia soredians*	Lecanorales	NO, Rogaland, 2013, E. Nygaard	BG-L-98015	OLICH2437-16	MK812200	
*Flavoplaca citrina*	Teloschistales	NO, Nord-Trøndelag, 2015, V. Alstrup	O-L-201322	OLICH2845-16	MK811952	
*Flavoplaca citrina*	Teloschistales	NO, Sør-Trøndelag, 2015, R. Haugan	O-L-204037	OLICH3891-17	MK812194	
*Flavoplaca flavocitrina*	Teloschistales	NO, Hedmark, 2015, R. Haugan	O-L-204199	OLICH3887-17	MK811821	
*Flavoplaca marina*	Teloschistales	NO, Rogaland, 2010, J.T. Klepsland	O-L-200087	OLICH3206-16	MK811686	
*Flavoplaca marina*	Teloschistales	NO, Rogaland, 2014, J.T. Klepsland	O-L-200616	OLICH3207-16	MK811757	
*Frutidella caesioatra*	Lecanorales	NO, Troms, 2014, J.T. Klepsland	O-L-200359	OLICH3257-16	MK812163	
*Frutidella caesioatra*	Lecanorales	NO, Sogn og Fjordane, 2010, E. Timdal	O-L-163395	OLICH387-13	MK811625	
*Frutidella caesioatra*	Lecanorales	NO, Nordland, 2010, E. Timdal	O-L-163746	OLICH388-13	MK812446	
*Frutidella caesioatra*	Lecanorales	NO, Nordland, 2010, E. Timdal	O-L-163752	OLICH390-13	MK812346	
*Frutidella caesioatra*	Lecanorales	NO, Oppland, 2013, M. Bendiksby et al.	O-L-184399	OLICH657-13	MK812561	
*Fuscidea mollis*	Umbilicariales	NO, Finnmark, 2014, M. Westberg	O-L-195707	OLICH1579-14	KY266980	divaricatic acid
*Fuscidea mollis*	Umbilicariales	NO, Finnmark, 2014, E. Timdal	O-L-195917	OLICH1762-14	KY266834	divaricatic acid
*Fuscopannaria ahlneri*	Peltigerales	NO, Nordland, 2008, T.H. Hofton	O-L-186679	OLICH2464-16	MK812382	
*Fuscopannaria confusa*	Peltigerales	NO, Nordland, 2013, J.T. Klepsland	O-L-198017	OLICH2465-16	MK812003	
*Fuscopannaria ignobilis*	Peltigerales	NO, Nord-Trøndelag, 2015, E. Timdal et al.	O-L-201286	OLICH2675-16	MK811940	
*Fuscopannaria ignobilis*	Peltigerales	NO, Nord-Trøndelag, 2015, M. Bendiksby et al.	O-L-201281	OLICH2778-16	MK812023	
*Fuscopannaria ignobilis*	Peltigerales	NO, Sør-Trøndelag, 2015, H. Holien	TRH-L-16696	OLICH3629-17	MK812459	
*Fuscopannaria ignobilis*	Peltigerales	NO, Sør-Trøndelag, 2012, H. Fjeldstad	O-L-203080	OLICH4062-17	MK812103	
*Fuscopannaria mediterranea*	Peltigerales	NO, Sogn og Fjordane, 2013, J.T. Klepsland	O-L-197869	OLICH2096-15	MK812182	
*Fuscopannaria mediterranea*	Peltigerales	NO, Hordaland, 2013, B. Nordén & J.B. Jordal	O-L-199530	OLICH3166-16	MK811995	
*Fuscopannaria praetermissa*	Peltigerales	NO, Telemark, 2014, E. Timdal	O-L-196265	OLICH1940-15	MK812190	
*Fuscopannaria praetermissa*	Peltigerales	NO, Nordland, 2013, J.T. Klepsland	O-L-198031	OLICH2097-15	MK811734	
*Fuscopannaria praetermissa*	Peltigerales	NO, Finnmark, 2003, E. Timdal	O-L-123194	OLICH3883-17	MK812710	
*Fuscopannaria praetermissa*	Peltigerales	NO, Troms, 2009, J.T. Klepsland	O-L-174932	OLICH3884-17	MK811837	
*Gyalecta flotowii*	Ostropales	NO, Hordaland, 2013, J.B. Jordal	O-L-199472	OLICH3165-16	MK811791	
*Gyalecta flotowii*	Ostropales	NO, Sogn og Fjordane, 2013, H. Fjeldstad	O-L-203030	OLICH4063-17	MK811730	
*Gyalecta foveolaris*	Ostropales	NO, Finnmark, 2014, T.B. Wheeler	O-L-195735	OLICH1607-14	KY266833	
*Gyalecta friesii*	Ostropales	NO, Sør-Trøndelag, 2014, S. Vatne	O-L-200781	OLICH2442-16	MK812083	
*Gyalecta geoica*	Ostropales	NO, Sør-Trøndelag, 2014, J.T. Klepsland	O-L-200544	OLICH3164-16	MK811956	
*Gyalecta jenensis*	Ostropales	NO, Finnmark, 2014, M. Westberg	O-L-195708	OLICH1580-14	KY266916	
*Gyalecta jenensis*	Ostropales	NO, Finnmark, 2014, A. Millanes	O-L-195860	OLICH1705-14	KY266964	
*Gyalecta jenensis*	Ostropales	NO, Nordland, 2013, J.T. Klepsland	O-L-198022	OLICH4034-17	MK811684	
*Gyalecta ulmi*	Ostropales	NO, Sør-Trøndelag, 2014, S. Vatne	O-L-200778	OLICH2443-16	MK812657	
*Gyalolechia bracteata*	Teloschistales	NO, Finnmark, 2011, E. Timdal	O-L-170861	OLICH3215-16	MK811756	
*Gyalolechia flavorubescens*	Teloschistales	NO, Telemark, 2014, E. Timdal	O-L-195786	OLICH1658-14	MK811715	
*Gyrographa gyrocarpa*	Arthoniales	NO, Nord-Trøndelag, 2015, H. Holien	TRH-L-16879	OLICH3651-17	MK812164	
*Gyrographa gyrocarpa*	Arthoniales	NO, Aust-Agder, 2015, J.T. Klepsland	O-L-204523	OLICH4234-17	MK812125	
*Haematomma ochroleucum*	Lecanorales	NO, Hordaland, 2015, T. Tønsberg	BG-L-99143	OLICH3704-17	MK812424	
*Haematomma ochroleucum*	Lecanorales	NO, Nordland, 2015, T. Tønsberg	BG-L-99181	OLICH3749-17	MK812361	
*Haematomma ochroleucum*	Lecanorales	NO, Sør-Trøndelag, 2014, R. Haugan	O-L-196048	OLICH3817-17	MK812222	
*Halecania alpivaga*	Leprocaulales	NO, Finnmark, 2014, M. Westberg	O-L-195847	OLICH1692-14	KY266832	
*Hertelidea botryosa*	Lecanorales	NO, Hedmark, 2016, E. Timdal	O-L-201530	OLICH3988-17	MK811999	
*Hertelidea botryosa*	Lecanorales	NO, Sør-Trøndelag, 2007, E. Timdal	O-L-158480	OLICH415-13	MK812335	
*Heterodermia speciosa*	Caliciales	NO, Buskerud, 2009, T.H. Hofton	O-L-186983	OLICH1454-14	MK812396	
*Heterodermia speciosa*	Caliciales	NO, Oppland, 2011, J.T. Klepsland	O-L-176833	OLICH2287-15	MK812055	
*Heterodermia speciosa*	Caliciales	NO, Oppland, 2011, J.T. Klepsland	O-L-176831	OLICH2288-15	MK812697	
*Heterodermia speciosa*	Caliciales	NO, Oppland, 2011, J.T. Klepsland	O-L-176827	OLICH2290-15	MK812504	
*Heterodermia speciosa*	Caliciales	NO, Oppland, 2011, T. Høitomt et al.	O-L-176666	OLICH2291-15	MK812537	
*Heterodermia speciosa*	Caliciales	NO, Oppland, 2011, J.T. Klepsland	O-L-176840	OLICH2292-15	MK812549	
*Heterodermia speciosa*	Caliciales	NO, Oppland, 2011, J.T. Klepsland	O-L-176824	OLICH2293-15	MK811755	
*Heterodermia speciosa*	Caliciales	NO, Hedmark, 2011, R. Haugan	O-L-173228	OLICH2294-15	MK812405	
*Hypocenomyce scalaris*	Umbilicariales	SE, Dalarna, 2014, E. Timdal	O-L-195692	OLICH2014-15	MK812269	
*Hypocenomyce scalaris*	Umbilicariales	NO, Telemark, 2013, J.T. Klepsland	O-L-198107	OLICH2352-15	MK811723	
*Hypocenomyce scalaris*	Umbilicariales	NO, Telemark, 2013, J.T. Klepsland	O-L-198111	OLICH2366-15	MK812170	
*Hypocenomyce scalaris*	Umbilicariales	NO, Hedmark, 2016, E. Timdal	O-L-201526	OLICH3987-17	MK811809	
*Hypocenomyce scalaris*	Umbilicariales	SE, Jämtland, 2017, E. Timdal	O-L-208182	OLICH4285-17	MK812211	
*Hypogymnia austerodes*	Lecanorales	NO, Hedmark, 2013, R. Haugan	O-L-190094	OLICH1466-14	MK812650	
*Hypogymnia austerodes*	Lecanorales	NO, Sør-Trøndelag, 2014, E. Timdal	O-L-196349	OLICH1908-15	MK812410	
*Hypogymnia austerodes*	Lecanorales	NO, Sør-Trøndelag, 2014, J.T. Klepsland	O-L-200537	OLICH2613-16	MK812531	
*Hypogymnia bitteri*	Lecanorales	NO, Buskerud, 2013, S. Rui & E. Timdal	O-L-184690	OLICH874-13	MK811649	
*Hypogymnia farinacea*	Lecanorales	NO, Akershus, 2014, E. Timdal	O-L-196258	OLICH2265-15	MK811676	
*Hypogymnia farinacea*	Lecanorales	NO, Hedmark, 2010, R. Haugan	O-L-183979	OLICH2279-15	MK812081	
*Hypogymnia farinacea*	Lecanorales	NO, Hedmark, 2010, R. Haugan	O-L-183983	OLICH2280-15	MK812607	
*Hypogymnia farinacea*	Lecanorales	NO, Hedmark, 2010, R. Haugan	O-L-183984	OLICH2281-15	MK812314	
*Hypogymnia farinacea*	Lecanorales	NO, Hedmark, 2011, R. Haugan	O-L-174340	OLICH2283-15	MK812440	
*Hypogymnia farinacea*	Lecanorales	NO, Hedmark, 2010, R. Haugan	O-L-183985	OLICH2285-15	MK812305	
*Hypogymnia farinacea*	Lecanorales	NO, Buskerud, 2013, J.T. Klepsland	O-L-198103	OLICH2369-15	MK812704	
*Hypogymnia farinacea*	Lecanorales	NO, Nord-Trøndelag, 2015, M. Bendiksby et al.	O-L-201310	OLICH2785-16	MK811721	
*Hypogymnia hultenii*	Lecanorales	NO, Nord-Trøndelag, 2015, M. Bendiksby et al.	O-L-201260	OLICH2767-16	MK811640	
*Hypogymnia incurvoides*	Lecanorales	NO, Rogaland, 2012, J.T. Klepsland	O-L-186120	OLICH1433-14	MK811919	atranorin, physodalic acid, physodic acid, protocetraric acid (minor)
*Hypogymnia incurvoides*	Lecanorales	NO, Rogaland, 2012, J.T. Klepsland	O-L-186139	OLICH1435-14	MK812392	atranorin, physodalic acid, physodic acid, protocetraric acid (minor)
*Hypogymnia incurvoides*	Lecanorales	NO, Nord-Trøndelag, 2014, J.T. Klepsland	O-L-200733	OLICH2620-16	MK812655	atranorin, physodalic acid, physodic acid
*Hypogymnia incurvoides*	Lecanorales	NO, Hordaland, 2014, J.T. Klepsland	O-L-200683	OLICH3161-16	MK812638	atranorin, physodalic acid, physodic acid
*Hypogymnia physodes*	Lecanorales	NO, Finnmark, 2014, E. Timdal	O-L-195888	OLICH1733-14	KY266854	
*Hypogymnia physodes*	Lecanorales	NO, Finnmark, 2014, M. Westberg	O-L-195921	OLICH1766-14	KY266919	
*Hypogymnia physodes*	Lecanorales	NO, Telemark, 2015, S. Rui & E. Timdal	O-L-200883	OLICH2565-16	MK811818	
*Hypogymnia physodes*	Lecanorales	NO, Hordaland, 2014, J.T. Klepsland	O-L-200741	OLICH2621-16	MK812700	3-hydroxyphysodic acid, atranorin, physodalic acid, physodic acid
*Hypogymnia physodes*	Lecanorales	NO, Nord-Trøndelag, 2014, J.T. Klepsland	O-L-200731	OLICH3158-16	MK812007	3-hydroxyphysodic acid, atranorin, physodalic acid, physodic acid
*Hypogymnia physodes*	Lecanorales	NO, Nordland, 2014, J.T. Klepsland	O-L-200736	OLICH3159-16	MK812416	3-hydroxyphysodic acid, atranorin, physodalic acid, physodic acid
*Hypogymnia physodes*	Lecanorales	NO, Akershus, 2014, J.T. Klepsland	O-L-200255	OLICH3160-16	MK812648	
*Hypogymnia physodes*	Lecanorales	NO, Buskerud, 2013, S. Rui & E. Timdal	O-L-184691	OLICH875-13	MK812139	
*Hypogymnia tubulosa*	Lecanorales	NO, Telemark, 2014, S. Rui & E. Timdal	O-L-194145	OLICH1480-14	MK811733	
*Hypogymnia tubulosa*	Lecanorales	NO, Rogaland, 2015, T. Tønsberg & E. Nygaard	BG-L-98167	OLICH3698-17	MK812120	
*Hypogymnia tubulosa*	Lecanorales	NO, Buskerud, 2013, S. Rui & E. Timdal	O-L-184692	OLICH876-13	MK811664	
*Hypogymnia vittata*	Lecanorales	NO, Buskerud, 2008, G. Gaarder	O-L-155319	OLICH1368-14	MK811984	
*Hypogymnia vittata*	Lecanorales	NO, Troms, 2014, E. Timdal	O-L-195648	OLICH1560-14	MK812063	
*Hypogymnia vittata*	Lecanorales	NO, Telemark, 2015, S. Rui & E. Timdal	O-L-200917	OLICH2577-16	MK812228	
*Hypogymnia vittata*	Lecanorales	NO, Sør-Trøndelag, 2014, J.T. Klepsland	O-L-200536	OLICH2612-16	MK812445	atranorin, physodic acid, vittatolic acid
*Hypogymnia vittata*	Lecanorales	NO, Akershus, 2017, S. Rui & E. Timdal	O-L-206863	OLICH4108-17	MK812679	
*Hypotrachyna afrorevoluta*	Lecanorales	NO, Rogaland, 2011, J.T. Klepsland	O-L-176869	OLICH2182-15	MK812595	
*Hypotrachyna afrorevoluta*	Lecanorales	NO, Rogaland, 2016, S. Rui & E. Timdal	O-L-205001	OLICH3991-17	MK811750	
*Hypotrachyna laevigata*	Lecanorales	NO, Rogaland, 2010, J.T. Klepsland	O-L-175129	OLICH1391-14	MK812534	
*Hypotrachyna laevigata*	Lecanorales	NO, Rogaland, 2011, J.T. Klepsland	O-L-176890	OLICH1397-14	MK812711	
*Hypotrachyna revoluta*	Lecanorales	NO, Rogaland, 2005, J.T. Klepsland	O-L-141145	OLICH1352-14	MK812078	
*Hypotrachyna revoluta*	Lecanorales	NO, Vest-Agder, 2013, J.T. Klepsland	O-L-197837	OLICH2099-15	MK812441	
*Hypotrachyna revoluta*	Lecanorales	NO, Rogaland, 2011, T. Høitomt	O-L-176684	OLICH2183-15	MK812141	
*Hypotrachyna revoluta*	Lecanorales	NO, Rogaland, 2016, S. Rui & E. Timdal	O-L-205002	OLICH3990-17	MK812580	
*Hypotrachyna sinuosa*	Lecanorales	NO, Rogaland, 2016, T. Tønsberg	BG-L-99501	OLICH3758-17	MK812199	
*Icmadophila ericetorum*	Pertusariales	NO, Buskerud, 2013, S. Rui & E. Timdal	O-L-184734	OLICH1416-14	MK812230	
*Icmadophila ericetorum*	Pertusariales	NO, Finnmark, 2014, E. Timdal	O-L-195557	OLICH1515-14	KY266881	
*Icmadophila ericetorum*	Pertusariales	NO, Finnmark, 2014, H. Holien	O-L-195756	OLICH1628-14	KY266943	
*Icmadophila ericetorum*	Pertusariales	NO, Finnmark, 2014, T. Tønsberg	O-L-195874	OLICH1719-14	KY266876	
*Icmadophila ericetorum*	Pertusariales	NO, Nord-Trøndelag, 2015, H. Holien	TRH-L-16544	OLICH3627-17	MK811879	
*Imshaugia aleurites*	Lecanorales	NO, Akershus, 2014, E. Timdal	O-L-196257	OLICH2264-15	MK812672	
*Japewia subaurifera*	Lecanorales	NO, Nord-Trøndelag, 2012, T. Tønsberg	BG-L-94252	OLICH577-13	MK812331	
*Japewia subaurifera*	Lecanorales	NO, Finnmark, 2012, T. Tønsberg	BG-L-94707	OLICH578-13	MK812698	
*Japewia subaurifera*	Lecanorales	NO, Nordland, 2012, T. Tønsberg	BG-L-94659	OLICH596-13	MK812623	
*Japewia tornoensis*	Lecanorales	NO, Buskerud, 2009, J.T. Klepsland	O-L-165163	OLICH357-13	MK812546	
*Japewia tornoensis*	Lecanorales	NO, Buskerud, 2011, J.T. Klepsland	O-L-176857	OLICH358-13	MK812668	
*Koerberiella wimmeriana*	Lecideales	NO, Hordaland, 2010, E. Timdal	O-L-163472	OLICH414-13	MK812168	
*Lasallia pustulata*	Umbilicariales	NO, Rogaland, 2016, S. Rui & E. Timdal	O-L-204990	OLICH4001-17	MK811849	
*Lecanactis abietina*	Arthoniales	SE, Södermanland, 2015, S. Kistenich & E. Timdal	O-L-200836	OLICH2424-16	MK812703	
*Lecanactis abietina*	Arthoniales	NO, Nord-Trøndelag, 2015, M. Bendiksby et al.	O-L-201261	OLICH2768-16	MK811953	
*Lecania aipospila*	Lecanorales	NO, Finnmark, 2014, J.T. Klepsland	O-L-200506	OLICH3254-16	MK812577	
*Lecania aipospila*	Lecanorales	NO, Finnmark, 2003, E. Timdal	O-L-123172	OLICH3962-17	MK811636	
*Lecania erysibe*	Lecanorales	NO, Oppland, 2011, J.T. Klepsland	O-L-183713	OLICH3956-17	MK812191	
*Lecanora atrosulphurea*	Lecanorales	NO, Finnmark, 2014, E. Timdal	O-L-195558	OLICH1516-14	KY266931	
*Lecanora intricata*	Lecanorales	NO, Finnmark, 2014, U. Arup	O-L-195841	OLICH1686-14	KY266891	
*Lecanora intricata*	Lecanorales	NO, Finnmark, 2014, E. Timdal	O-L-195876	OLICH1721-14	KY266975	
*Lecanora leptacina*	Lecanorales	NO, Finnmark, 2014, E. Timdal	O-L-195896	OLICH1741-14	KY266950	
*Lecidea auriculata*	Lecideales	NO, Finnmark, 2014, E. Timdal	O-L-195916	OLICH1761-14	KY266956	no lichen substances
*Lecidea leucothallina*	Lecideales	NO, Oppland, 2013, T. Tønsberg	BG-L-96170	OLICH1065-14	MG968256	pannarin (thallus)
*Lecidea leucothallina*	Lecideales	NO, Nordland, 2010, E. Timdal	O-L-163621	OLICH179-11	MG968259	pannarin (apothecium), no lichen substances (thallus)
*Lecidea leucothallina*	Lecideales	NO, Nordland, 2010, E. Timdal	O-L-163673	OLICH180-11	MG968258	pannarin (thallus)
*Lecidea leucothallina*	Lecideales	NO, Møre og Romsdal, 2010, R. Haugan	O-L-165815	OLICH181-11	MG968260	no lichen substances (thallus)
*Lecidea toensbergii*	Lecideales	NO, Oppland, 2013, T. Tønsberg	BG-L-95936	OLICH1010-13	MG968261	pannarin (thallus)
*Lecidea toensbergii*	Lecideales	NO, Møre og Romsdal, 2010, R. Haugan	O-L-165806	OLICH182-11	MG968257	pannarin (thallus)
*Leprocaulon quisquiliare*	Leprocaulales	NO, Telemark, 2015, S. Rui & E. Timdal	O-L-200884	OLICH2566-16	MK812636	
*Leprocaulon quisquiliare*	Leprocaulales	NO, Akershus, 2015, S. Rui & E. Timdal	O-L-200816	OLICH2587-16	MK811899	
*Leprocaulon quisquiliare*	Leprocaulales	NO, Østfold, 2017, S. Rui & E. Timdal	O-L-208334	OLICH4265-17	MK812206	
*Leproplaca cirrochroa*	Teloschistales	NO, Oppland, 2011, J.T. Klepsland	O-L-183714	OLICH4395-17	MK812049	
*Leproplaca cirrochroa*	Teloschistales	NO, Telemark, 2009, R. Haugan & S. Reiso	O-L-160423	OLICH4427-17	MK812423	
*Leproplaca cirrochroa*	Teloschistales	NO, Nord-Trøndelag, 1998, R. Haugan, R. Hollas	O-L-44774	OLICH4428-17	MK812709	
*Leproplaca obliterans*	Teloschistales	NO, Akershus, 2013, J.T. Klepsland	O-L-197812	OLICH4426-17	MK812503	
*Leptogium cochleatum*	Peltigerales	NO, Hordaland, 2013, B. Nordén & J.B. Jordal	O-L-199489	OLICH3149-16	MK812473	
*Leptogium cochleatum*	Peltigerales	NO, Hordaland, 2013, B. Nordén & J.B. Jordal	O-L-199545	OLICH3150-16	MK811946	
*Leptogium saturninum*	Peltigerales	NO, Finnmark, 2014, E. Timdal	O-L-195592	OLICH1533-14	MK812662	
*Leptogium saturninum*	Peltigerales	NO, Hordaland, 2013, B. Nordén & J.B. Jordal	O-L-199516	OLICH3250-16	MK812179	
*Leptogium saturninum*	Peltigerales	NO, Sør-Trøndelag, 2014, R. Haugan	O-L-196046	OLICH3800-17	MK811641	
*Letharia vulpina*	Lecanorales	NO, Hedmark, 2012, R. Haugan	O-L-184032	OLICH1405-14	MK812266	unknown acid, vulpinic acid
*Letharia vulpina*	Lecanorales	NO, Hedmark, 2012, R. Haugan	O-L-184029	OLICH2179-15	MK812109	
*Letharia vulpina*	Lecanorales	NO, Hedmark, 2011, B. Skinderhaug	O-L-169053	OLICH2180-15	MK811718	
*Letharia vulpina*	Lecanorales	NO, Hedmark, 2010, R. Haugan	O-L-165378	OLICH2181-15	MK811860	
*Letharia vulpina*	Lecanorales	NO, Hedmark, 2014, R. Haugan	O-L-198890	OLICH2395-16	MK811758	
*Lichenomphalia alpina*	Agaricales	NO, Finnmark, 2014, H. Holien	O-L-195732	OLICH1604-14	KY266895	
*Lichenomphalia velutina*	Agaricales	NO, Oslo, 2014, E. Timdal	O-L-200004	OLICH1978-15	MK811822	
*Lobaria amplissima*	Peltigerales	NO, Sør-Trøndelag, 2014, R. Haugan	O-L-196021	OLICH1866-14	MK812432	
*Lobaria amplissima*	Peltigerales	NO, Sør-Trøndelag, 2010, J.T. Klepsland	O-L-174993	OLICH2176-15	MK812486	
*Lobaria amplissima*	Peltigerales	NO, Sør-Trøndelag, 2013, J.T. Klepsland	O-L-198062	OLICH2326-15	MK811832	
*Lobaria amplissima*	Peltigerales	NO, Buskerud, 2011, T.H. Hofton	O-L-194379	OLICH2327-15	MK811802	
*Lobaria amplissima*	Peltigerales	NO, Sogn og Fjordane, 2010, T.H. Hofton	O-L-187118	OLICH2328-15	MK812689	
*Lobaria amplissima*	Peltigerales	NO, Sør-Trøndelag, 2011, J.T. Klepsland	O-L-176912	OLICH2329-15	MK812498	
*Lobaria amplissima*	Peltigerales	NO, Nord-Trøndelag, 2012, J.T. Klepsland	O-L-186329	OLICH2330-15	MK811725	
*Lobaria amplissima*	Peltigerales	NO, Nord-Trøndelag, 2013, J.T. Klepsland	O-L-198158	OLICH2387-16	MK812569	
*Lobaria amplissima*	Peltigerales	NO, Nordland, 2003, A. Granmo & L. Molster	TROM_L_46394	OLICH3586-17	MK811901	
*Lobaria linita*	Peltigerales	NO, Finnmark, 2014, E. Timdal	O-L-195728	OLICH1600-14	KY266856	
*Lobaria linita*	Peltigerales	NO, Finnmark, 2014, E. Timdal	O-L-195892	OLICH1737-14	KY266932	
*Lobaria pulmonaria*	Peltigerales	NO, Sør-Trøndelag, 2014, R. Haugan	O-L-196024	OLICH1869-14	MK811957	
*Lobaria pulmonaria*	Peltigerales	NO, Sør-Trøndelag, 2014, E. Timdal	O-L-196356	OLICH1915-15	MK811682	
*Lobaria pulmonaria*	Peltigerales	NO, Østfold, 2013, O. Wergeland Krog	O-L-193007	OLICH2335-15	MK812447	
*Lobaria pulmonaria*	Peltigerales	NO, Østfold, 2012, O. Wergeland Krog et al.	O-L-193009	OLICH2336-15	MK812330	
*Lobaria pulmonaria*	Peltigerales	NO, Østfold, 2013, O. Wergeland Krog	O-L-193011	OLICH2337-15	MK812533	
*Lobaria pulmonaria*	Peltigerales	NO, Hedmark, 2013, R. Haugan	O-L-190366	OLICH2338-15	MK812202	
*Lobaria pulmonaria*	Peltigerales	NO, Buskerud, 2011, T.H. Hofton & S. Reiso	O-L-194285	OLICH2339-15	MK812005	
*Lobaria pulmonaria*	Peltigerales	NO, Buskerud, 2012, T.H. Hofton	O-L-194456	OLICH2340-15	MK812631	
*Lobaria pulmonaria*	Peltigerales	NO, Vest-Agder, 2011, J.T. Klepsland	O-L-177093	OLICH2346-15	MK812031	
*Lobaria pulmonaria*	Peltigerales	NO, Hedmark, 2012, R. Haugan	O-L-183966	OLICH2347-15	MK812039	
*Lobaria pulmonaria*	Peltigerales	SE, Södermanland, 2015, S. Kistenich & E. Timdal	O-L-200863	OLICH2515-16	MK811954	
*Lobaria pulmonaria*	Peltigerales	NO, Troms, 2005, A. Granmo	TROM_L_50934	OLICH3536-17	MK811932	
*Lobaria scrobiculata*	Peltigerales	NO, Sør-Trøndelag, 2014, E. Timdal	O-L-196350	OLICH1909-15	MK812570	
*Lobaria scrobiculata*	Peltigerales	NO, Østfold, 2013, B.P. Løfall & O. Wergeland Krog	O-L-187365	OLICH2341-15	MK812073	
*Lobaria scrobiculata*	Peltigerales	NO, Hedmark, 2013, R. Haugan	O-L-190098	OLICH2342-15	MK812310	
*Lobaria scrobiculata*	Peltigerales	NO, Buskerud, 2012, T.H. Hofton	O-L-194516	OLICH2343-15	MK812214	
*Lobaria scrobiculata*	Peltigerales	NO, Rogaland, 2012, J.T. Klepsland	O-L-186129	OLICH2344-15	MK811651	
*Lobaria scrobiculata*	Peltigerales	NO, Nord-Trøndelag, 2010, H. Bratli	O-L-168086	OLICH2345-15	MK812309	
*Lobaria scrobiculata*	Peltigerales	NO, Troms, 2005, A. Granmo	TROM_L_50935	OLICH3537-17	MK812074	
*Lobaria scrobiculata*	Peltigerales	NO, Sør-Trøndelag, 2014, R. Haugan	O-L-196051	OLICH3810-17	MK811855	
*Lobaria virens*	Peltigerales	NO, Nord-Trøndelag, 2015, M. Bendiksby et al.	O-L-201271	OLICH2774-16	MK811660	
*Lobaria virens*	Peltigerales	NO, Nordland, 2014, J.T. Klepsland	O-L-200346	OLICH3249-16	MK811736	
*Lobaria virens*	Peltigerales	NO, Vestfold, 2003, T.H. Hofton et al.	O-L-128546	OLICH4103-17	MK812046	
*Lobothallia alphoplaca*	Pertusariales	NO, Finnmark, 2014, J.T. Klepsland	O-L-200411	OLICH2658-16	MK812484	
*Lobothallia melanaspis*	Pertusariales	SE, Torne Lappmark, 2014, E. Timdal	O-L-195680	OLICH2007-15	MK811796	
*Lobothallia melanaspis*	Pertusariales	NO, Hedmark, 2014, R. Haugan	O-L-198879	OLICH2390-16	MK811944	
*Loxospora elatina*	Sarrameanales	NO, Nord-Trøndelag, 2014, J.T. Klepsland	O-L-200270	OLICH2657-16	MK812411	
*Massalongia carnosa*	Peltigerales	NO, Aust-Agder, 2013, J.T. Klepsland	O-L-198320	OLICH2371-15	MK812187	
*Massalongia carnosa*	Peltigerales	NO, Buskerud, 2011, E. Timdal	O-L-175425	OLICH3285-16	MK811716	
*Megalaria grossa*	Lecanorales	NO, Akershus, 2010, J.T. Klepsland	O-L-174947	OLICH1036-13	MK811839	
*Megalaria grossa*	Lecanorales	NO, Sør-Trøndelag, 2005, E. Timdal	O-L-149362	OLICH1039-13	MK812151	
*Megalaria grossa*	Lecanorales	NO, Nordland, 2014, J.T. Klepsland	O-L-200308	OLICH2656-16	MK812492	
*Megalaria pulverea*	Lecanorales	NO, Sogn og Fjordane, 2009, J.T. Klepsland	O-L-165033	OLICH1033-13	MK812535	
*Megalaria pulverea*	Lecanorales	NO, Sør-Trøndelag, 2009, R. Haugan	O-L-167026	OLICH1034-13	MK812297	
*Megalaria pulverea*	Lecanorales	NO, Nord-Trøndelag, 2008, J.T. Klepsland	O-L-158214	OLICH1035-13	MK812205	
*Megalaria pulverea*	Lecanorales	NO, Hordaland, 2011, J.T. Klepsland	O-L-176983	OLICH1317-14	MK812565	
*Megalaria pulverea*	Lecanorales	NO, Nordland, 2013, J.T. Klepsland	O-L-197986	OLICH2104-15	MK812321	
*Melanelia agnata*	Lecanorales	NO, Sør-Trøndelag, 2014, E. Timdal	O-L-196376	OLICH1935-15	MK812394	
*Melanelia hepatizon*	Lecanorales	NO, Hordaland, 2014, E. Timdal	O-L-195807	OLICH1679-14	MK812512	
*Melanelia hepatizon*	Lecanorales	NO, Finnmark, 2014, M. Westberg	O-L-195864	OLICH1709-14	KY266879	
*Melanelia hepatizon*	Lecanorales	NO, Nord-Trøndelag, 2015, M. Bendiksby et al.	O-L-201254	OLICH2762-16	MK812070	
*Melanelia hepatizon*	Lecanorales	NO, Buskerud, 2013, S. Rui & E. Timdal	O-L-184723	OLICH939-13	MK812188	
*Melanelia stygia*	Lecanorales	NO, Buskerud, 2013, S. Rui & E. Timdal	O-L-184736	OLICH1418-14	MK812608	
*Melanelia stygia*	Lecanorales	NO, Sør-Trøndelag, 2014, E. Timdal	O-L-196377	OLICH1936-15	MK812312	
*Melanelixia fuliginosa*	Lecanorales	NO, Vestfold, 2015, E. Timdal	O-L-198943	OLICH2499-16	MK812485	
*Melanelixia fuliginosa*	Lecanorales	SE, Södermanland, 2015, S. Kistenich & E. Timdal	O-L-200866	OLICH2518-16	MK812619	
*Melanelixia fuliginosa*	Lecanorales	NO, Sør-Trøndelag, 2014, R. Haugan	O-L-196050	OLICH3818-17	MK812455	
*Melanelixia glabratula*	Lecanorales	NO, Hordaland, 2011, J.T. Klepsland	O-L-176965	OLICH4122-17	MK812674	
*Melanelixia subargentifera*	Lecanorales	NO, Buskerud, 2014, E. Timdal	O-L-200810	OLICH2475-16	MK812517	
*Melanelixia subargentifera*	Lecanorales	NO, Hedmark, 2015, E. Timdal	O-L-200854	OLICH2505-16	MK812491	
*Melanelixia subargentifera*	Lecanorales	SE, Södermanland, 2015, S. Kistenich & E. Timdal	O-L-200867	OLICH2519-16	MK811964	
*Melanelixia subargentifera*	Lecanorales	NO, Hedmark, 2014, R. Haugan	O-L-207985	OLICH3820-17	MK812324	
*Melanelixia subaurifera*	Lecanorales	NO, Sør-Trøndelag, 2014, R. Haugan	O-L-195997	OLICH1842-14	MK811631	
*Melanelixia subaurifera*	Lecanorales	NO, Akershus, 2014, E. Timdal	O-L-199993	OLICH1967-15	MK811942	
*Melanelixia subaurifera*	Lecanorales	NO, Oslo, 2014, E. Timdal	O-L-198930	OLICH2483-16	MK812688	
*Melanohalea exasperata*	Lecanorales	NO, Sør-Trøndelag, 2014, R. Haugan	O-L-196008	OLICH1853-14	MK812395	
*Melanohalea exasperata*	Lecanorales	NO, Telemark, 2015, E. Timdal	O-L-208004	OLICH3878-17	MK812307	
*Melanohalea exasperata*	Lecanorales	NO, Sogn og Fjordane, 2004, A. Breili	O-L-151072	OLICH4123-17	MK812184	
*Melanohalea infumata*	Lecanorales	NO, Troms, 2003, E. Timdal	O-L-123135	OLICH4126-17	MK812651	
*Melanohalea olivacea*	Lecanorales	NO, Finnmark, 2014, E. Timdal	O-L-195933	OLICH1778-14	KY266962	
*Melanohalea olivacea*	Lecanorales	NO, Buskerud, 2014, E. Timdal	O-L-195826	OLICH2198-15	MK812054	
*Melanohalea olivacea*	Lecanorales	NO, Telemark, 2015, E. Timdal	O-L-208005	OLICH3879-17	MK812340	
*Melanohalea septentrionalis*	Lecanorales	NO, Akershus, 2017, S. Rui & E. Timdal	O-L-206865	OLICH4110-17	MK812433	
*Menegazzia subsimilis*	Lecanorales	NO, Rogaland, 2007, E. Timdal	O-L-149092	OLICH864-13	MK812421	
*Menegazzia subsimilis*	Lecanorales	NO, Rogaland, 2012, J.T. Klepsland	O-L-186134	OLICH944-13	MK812020	
*Menegazzia terebrata*	Lecanorales	SE, Södermanland, 2015, S. Kistenich & E. Timdal	O-L-200861	OLICH2513-16	MK812093	
*Menegazzia terebrata*	Lecanorales	NO, Telemark, 2015, S. Rui & E. Timdal	O-L-200924	OLICH2532-16	MK812635	
*Menegazzia terebrata*	Lecanorales	NO, Hordaland, 2013, T. Tønsberg	BG-L-95692	OLICH858-13	MK812654	
*Menegazzia terebrata*	Lecanorales	NO, Oppland, 2011, J.T. Klepsland	O-L-176828	OLICH861-13	MK812025	
*Menegazzia terebrata*	Lecanorales	NO, Rogaland, 2012, J.T. Klepsland	O-L-186132	OLICH949-13	MK812145	
*Micarea botryoides*	Lecanorales	NO, Rogaland, 2013, J.I. Johnsen	BG-L-95780	OLICH1006-13	MK812289	
*Micarea denigrata*	Lecanorales	NO, Oppland, 2011, R. Haugan & A.M. Haugan	O-L-174438	OLICH1013-13	MK811696	
*Micarea misella*	Lecanorales	NO, Nord-Trøndelag, 2013, H. Holien	TRH-L-15146	OLICH990-13	MK812399	
*Micarea paratropa*	Lecanorales	NO, Sør-Trøndelag, 2010, R. Haugan	O-L-165711	OLICH101-11	MK812318	no lichen substances
*Montanelia disjuncta*	Lecanorales	NO, Finnmark, 2014, E. Timdal	O-L-195590	OLICH1531-14	MK811852	
*Montanelia disjuncta*	Lecanorales	NO, Sør-Trøndelag, 2014, E. Timdal	O-L-196357	OLICH1916-15	MK811711	
*Montanelia disjuncta*	Lecanorales	NO, Finnmark, 2014, R. Haugan	O-L-198675	OLICH2404-16	KY266910	
*Montanelia panniformis*	Lecanorales	NO, Finnmark, 2014, R. Haugan	O-L-195904	OLICH1749-14	KY266847	
*Montanelia panniformis*	Lecanorales	NO, Buskerud, 2014, S. Rui & E. Timdal	O-L-200016	OLICH1990-15	MK812407	
*Montanelia panniformis*	Lecanorales	NO, Telemark, 2015, S. Rui & E. Timdal	O-L-200878	OLICH2535-16	MK812634	
*Montanelia panniformis*	Lecanorales	NO, Oppland, 2015, E. Timdal	O-L-201338	OLICH2722-16	MK812263	
*Montanelia sorediata*	Lecanorales	NO, Telemark, 2014, E. Timdal	O-L-195791	OLICH1663-14	MK811963	
*Montanelia sorediata*	Lecanorales	NO, Troms, 2014, E. Timdal	O-L-195658	OLICH1998-15	MK811965	
*Montanelia sorediata*	Lecanorales	NO, Buskerud, 2016, M. Stapnes Dahl et al.	O-L-204941	OLICH4115-17	MK811977	
*Montanelia tominii*	Lecanorales	NO, Oppland, 2015, E. Timdal	O-L-201344	OLICH2728-16	MK812373	
*Mycobilimbia berengeriana*	Lecideales	NO, Aust-Agder, 2011, J.T. Klepsland	O-L-177070	OLICH1113-14	MK812040	
*Mycobilimbia berengeriana*	Lecideales	NO, Oppland, 2011, J.T. Klepsland	O-L-176838	OLICH1114-14	MK812343	
*Mycobilimbia berengeriana*	Lecideales	NO, Finnmark, 2011, E. Timdal	O-L-170554	OLICH1117-14	MK811751	no lichen substances
*Mycobilimbia berengeriana*	Lecideales	NO, Aust-Agder, 2009, J.T. Klepsland	O-L-167344	OLICH1170-14	MK811880	
*Mycobilimbia berengeriana*	Lecideales	NO, Finnmark, 2014, E. Llop Vallverdu	O-L-195696	OLICH1568-14	KY266871	
*Mycobilimbia berengeriana*	Lecideales	NO, Sør-Trøndelag, 2004, R. Haugan	O-L-142068	OLICH629-13	MK811889	
*Mycobilimbia carneoalbida*	Lecideales	NO, Telemark, 2010, J.T. Klepsland	O-L-175294	OLICH1109-14	MK812353	
*Mycobilimbia carneoalbida*	Lecideales	NO, Aust-Agder, 2005, J.T. Klepsland	O-L-151121	OLICH1120-14	MK811646	
*Mycobilimbia carneoalbida*	Lecideales	NO, Troms, 2009, J.T. Klepsland	O-L-164904	OLICH1121-14	MK812149	
*Mycobilimbia carneoalbida*	Lecideales	NO, Oppland, 2011, R. Haugan	O-L-174298	OLICH1122-14	MK812290	
*Mycobilimbia carneoalbida*	Lecideales	NO, Hedmark, 2011, R. Haugan	O-L-174282	OLICH1123-14	MK812691	
*Mycobilimbia carneoalbida*	Lecideales	NO, Telemark, 2007, E. Timdal	O-L-149057	OLICH1124-14	MK812639	
*Mycobilimbia epixanthoides*	Lecideales	NO, Hedmark, 2006, J.T. Klepsland	O-L-164598	OLICH1105-14	MK812105	
*Mycobilimbia epixanthoides*	Lecideales	NO, Aust-Agder, 2006, J.T. Klepsland	O-L-151124	OLICH1106-14	MK812388	
*Mycobilimbia epixanthoides*	Lecideales	NO, Troms, 2007, J.T. Klepsland	O-L-154903	OLICH1107-14	MK812645	
*Mycobilimbia epixanthoides*	Lecideales	NO, Telemark, 2007, E. Timdal	O-L-149055	OLICH1108-14	MK812402	no lichen substances
*Mycobilimbia epixanthoides*	Lecideales	NO, Akershus, 2000, R. Haugan	O-L-160303	OLICH1112-14	MK811772	
*Mycobilimbia pilularis*	Lecideales	NO, Vest-Agder, 2009, J.T. Klepsland	O-L-164694	OLICH1110-14	MK812275	
*Mycobilimbia pilularis*	Lecideales	NO, Møre og Romsdal, 2012, B. Nordén & J.B. Jordal	O-L-180158	OLICH1111-14	MK812666	
*Mycobilimbia pilularis*	Lecideales	NO, Hordaland, 2008, J.B. Jordal	O-L-176020	OLICH1125-14	MK812525	
*Mycobilimbia pilularis*	Lecideales	NO, Sogn og Fjordane, 2009, A. Breili	O-L-159489	OLICH1126-14	MK812665	
*Mycobilimbia tetramera*	Lecideales	NO, Finnmark, 2011, E. Timdal	O-L-170621	OLICH1119-14	MK812422	no lichen substances
*Mycobilimbia tetramera*	Lecideales	NO, Nordland, 2011, R. Haugan	O-L-174132	OLICH1127-14	MK812216	no lichen substances
*Mycobilimbia tetramera*	Lecideales	NO, Aust-Agder, 2006, J.T. Klepsland	O-L-183493	OLICH1128-14	MK811935	
*Mycobilimbia tetramera*	Lecideales	NO, Finnmark, 2014, E. Llop Vallverdu	O-L-195846	OLICH1691-14	MK812124	
*Mycobilimbia tetramera*	Lecideales	NO, Akershus, 2014, J.T. Klepsland	O-L-200258	OLICH3147-16	MK812192	
*Mycobilimbia tetramera*	Lecideales	NO, Akershus, 2009, J.T. Klepsland	O-L-164634	OLICH631-13	MK812158	
*Mycobilimbia tetramera*	Lecideales	NO, Hedmark, 2008, R. Haugan	O-L-160476	OLICH633-13	MK812208	
*Mycoblastus sanguinarius*	Lecanorales	NO, Finnmark, 2014, E. Timdal	O-L-195768	OLICH1640-14	MK812482	
*Mycoblastus sanguinarius*	Lecanorales	NO, Nord-Trøndelag, 2015, M. Bendiksby et al.	O-L-201263	OLICH2769-16	MK811859	
*Mycoblastus sanguinarius*	Lecanorales	NO, Hedmark, 2010, E. Timdal	O-L-163524	OLICH391-13	MK811665	
*Mycoblastus sanguinarius*	Lecanorales	NO, Telemark, 2005, E. Timdal	O-L-138597	OLICH410-13	MK812197	
*Mycoblastus sanguinarius*	Lecanorales	NO, Oppland, 2006, A. Breili	O-L-150999	OLICH617-13	MK812135	
*Myriolecis straminea*	Lecanorales	NO, Finnmark, 2014, M. Westberg	O-L-195870	OLICH1715-14	KY266929	
*Myriospora smaragdula*	Acarosporales	NO, Finnmark, 2014, E. Timdal	O-L-195609	OLICH1543-14	MK811918	
*Nephroma arcticum*	Peltigerales	NO, Troms, 2014, E. Timdal	O-L-195654	OLICH1566-14	MK811882	
*Nephroma arcticum*	Peltigerales	NO, Finnmark, 2014, S. Rui	O-L-195724	OLICH1596-14	KY266888	
*Nephroma arcticum*	Peltigerales	NO, Finnmark, 2014, A. Millanes	O-L-195873	OLICH1718-14	KY266963	
*Nephroma arcticum*	Peltigerales	NO, Sør-Trøndelag, 2014, E. Timdal	O-L-196368	OLICH1927-15	MK812091	
*Nephroma arcticum*	Peltigerales	NO, Telemark, 2014, E. Timdal	O-L-196272	OLICH1945-15	MK812336	
*Nephroma arcticum*	Peltigerales	NO, Nord-Trøndelag, 2014, Z. Fackovcova et al.	SAV-36	OLICH3073-16	MK812204	
*Nephroma arcticum*	Peltigerales	NO, Nord-Trøndelag, 2014, Z. Fackovcova et al.	SAV-37	OLICH3074-16	MK812592	
*Nephroma arcticum*	Peltigerales	NO, Finnmark, 2008, A. Granmo & G. Mathiassen	TROM_L_46162	OLICH3570-17	MK811792	
*Nephroma arcticum*	Peltigerales	NO, Hedmark, 2014, R. Haugan	O-L-207992	OLICH3828-17	MK812028	
*Nephroma bellum*	Peltigerales	NO, Nord-Trøndelag, 2010, H. Bratli	O-L-168043	OLICH2027-15	MK811672	triterpenoids
*Nephroma bellum*	Peltigerales	NO, Sogn og Fjordane, 2012, O. Roen	O-L-181644	OLICH2028-15	MK812308	triterpenoids
*Nephroma bellum*	Peltigerales	NO, Sør-Trøndelag, 2014, E. Timdal	O-L-196348	OLICH2373-15	MK812483	triterpenoids
*Nephroma expallidum*	Peltigerales	NO, Finnmark, 2014, M. Westberg	O-L-195701	OLICH1573-14	KY266905	
*Nephroma expallidum*	Peltigerales	NO, Finnmark, 2014, E. Timdal	O-L-195891	OLICH1736-14	KY266844	
*Nephroma expallidum*	Peltigerales	NO, Sør-Trøndelag, 2014, E. Timdal	O-L-196346	OLICH1906-15	MK812408	
*Nephroma expallidum*	Peltigerales	NO, Finnmark, 2011, E. Timdal	O-L-170548	OLICH2365-15	MK811945	
*Nephroma expallidum*	Peltigerales	NO, Svalbard, 2015, S. Rui	O-L-203292	OLICH2712-16	MK812041	
*Nephroma helveticum*	Peltigerales	NO, Buskerud, 2013, T.H. Hofton	O-L-196491	OLICH2036-15	KT800007	triterpenoids
*Nephroma helveticum*	Peltigerales	NO, Oppland, 2011, J.T. Klepsland	O-L-181601	OLICH2037-15	KT800006	triterpenoides (T4, T1)
*Nephroma laevigatum*	Peltigerales	NO, Nord-Trøndelag, 2010, H. Bratli	O-L-168088	OLICH2025-15	MK812687	
*Nephroma laevigatum*	Peltigerales	NO, Sogn og Fjordane, 2010, J.T. Klepsland	O-L-175024	OLICH2038-15	MK812108	pigments, triterpenoid
*Nephroma laevigatum*	Peltigerales	NO, Nord-Trøndelag, 2015, E. Timdal et al.	O-L-201288	OLICH2676-16	MK812261	
*Nephroma laevigatum*	Peltigerales	NO, Troms, 2010, K. Wannebo Nilsen	TROM_L_48759	OLICH3531-17	MK811868	
*Nephroma laevigatum*	Peltigerales	NO, Sør-Trøndelag, 2014, R. Haugan	O-L-196055	OLICH3812-17	MK811969	
*Nephroma laevigatum*	Peltigerales	NO, Rogaland, 2016, S. Rui & E. Timdal	O-L-204997	OLICH4002-17	MK812002	
*Nephroma parile*	Peltigerales	NO, Finnmark, 2014, T. Tønsberg	O-L-195875	OLICH1720-14	KY266927	triterpenoids
*Nephroma resupinatum*	Peltigerales	NO, Hedmark, 2013, R. Haugan	O-L-190528	OLICH2020-15	MK811650	
*Nephroma resupinatum*	Peltigerales	NO, Sogn og Fjordane, 2010, E. Timdal	O-L-163401	OLICH2022-15	MK812367	
*Nephroma resupinatum*	Peltigerales	NO, Troms, 2011, J.T. Klepsland	O-L-177168	OLICH2023-15	MK812612	
*Nephroma resupinatum*	Peltigerales	NO, Nord-Trøndelag, 2011, J.T. Klepsland	O-L-183598	OLICH2024-15	MK811912	no lichen substances
*Nephroma resupinatum*	Peltigerales	NO, Troms, 2005, A. Granmo	TROM_L_50937	OLICH3538-17	MK812301	
*Nephroma resupinatum*	Peltigerales	NO, Finnmark, 2008, A. Granmo & G. Mathiassen	TROM_L_48235	OLICH3588-17	MK812553	
*Nephroma tangeriense*	Peltigerales	NO, Hordaland, 2012, J.T. Klepsland	O-L-186150	OLICH2018-15	MK811986	pigments, triterpenoides (T3, T4, T6)
*Nephroma tangeriense*	Peltigerales	NO, Rogaland, 2010, J.T. Klepsland	O-L-181602	OLICH2019-15	MK812084	pigments, triterpenoides (T2, T3, T6)
*Nephroma tangeriense*	Peltigerales	NO, Vest-Agder, 2011, J.T. Klepsland	O-L-183599	OLICH2039-15	MK812530	pigments, triterpenoids
*Nephroma tangeriense*	Peltigerales	NO, Vest-Agder, 2013, J.T. Klepsland	O-L-197824	OLICH2105-15	MK812579	pigments
*Nephroma tangeriense*	Peltigerales	NO, Vest-Agder, 2013, J.T. Klepsland	O-L-197826	OLICH2106-15	MK812593	
*Nevesia sampaiana*	Peltigerales	NO, Sogn og Fjordane, 2009, H. Fjeldstad	O-L-203003	OLICH3277-16	MK812089	
*Normandina pulchella*	Verrucariales	NO, Rogaland, 2016, S. Rui & E. Timdal	O-L-204993	OLICH4003-17	MK812131	
*Ochrolechia alaskana*	Pertusariales	NO, Finnmark, 2008, A. Granmo & G. Mathiassen	TROM_L_60552	OLICH3582-17	MK811874	
*Ochrolechia alboflavescens*	Pertusariales	NO, Nord-Trøndelag, 2015, F. Jonsson	O-L-201276	OLICH3230-16	MK812244	
*Ochrolechia androgyna*	Pertusariales	NO, Nord-Trøndelag, 2015, H. Holien	TRH-L-16947	OLICH3684-17	MK812264	
*Ochrolechia androgyna*	Pertusariales	NO, Oppland, 2015, R. Haugan	O-L-203919	OLICH3885-17	MK811897	atranorin, gyrophoric acid, lecanoric acid, unknown acid
*Ochrolechia androgyna*	Pertusariales	NO, Oppland, 2015, E. Timdal	O-L-201397	OLICH4178-17	MK812682	gyrophoric acid
*Ochrolechia frigida*	Pertusariales	NO, Finnmark, 2014, A. Launis	O-L-195945	OLICH1790-14	KY266952	
*Ochrolechia gowardii*	Pertusariales	NO, Nord-Trøndelag, 2014, J.T. Klepsland	O-L-200283	OLICH3146-16	MK812101	
*Ochrolechia gowardii*	Pertusariales	NO, Nord-Trøndelag, 2012, J.T. Klepsland	O-L-200100	OLICH3225-16	MK812175	
*Ochrolechia mahluensis*	Pertusariales	NO, Finnmark, 2014, H. Holien	O-L-195912	OLICH1757-14	KY266861	
*Ochrolechia microstictoides*	Pertusariales	NO, Aust-Agder, 2014, J.T. Klepsland	O-L-200753	OLICH3145-16	MK812077	
*Ochrolechia parella*	Pertusariales	NO, Sør-Trøndelag, 2014, R. Haugan	O-L-196015	OLICH1860-14	MK811779	
*Ochrolechia parella*	Pertusariales	NO, Nordland, 2014, J.T. Klepsland	O-L-200300	OLICH3144-16	MK811835	
*Ochrolechia subviridis*	Pertusariales	NO, Rogaland, 2014, J.T. Klepsland	O-L-200638	OLICH2655-16	MK812495	gyrophoric acid
*Ochrolechia szatalaensis*	Pertusariales	NO, Troms, 2011, J.T. Klepsland	O-L-200097	OLICH2653-16	MK811817	murolic acid (complex), variolaric acid
*Ochrolechia szatalaensis*	Pertusariales	NO, Hordaland, 2013, B. Nordén & J.B. Jordal	O-L-199529	OLICH2654-16	MK812256	
*Ochrolechia szatalaensis*	Pertusariales	NO, Nord-Trøndelag, 2014, J.T. Klepsland	O-L-200284	OLICH3143-16	MK811903	
*Ochrolechia szatalaensis*	Pertusariales	NO, Finnmark, 2011, J.T. Klepsland	O-L-200096	OLICH3227-16	MK811865	murolic acid (complex), variolaric acid
*Ochrolechia tartarea*	Pertusariales	NO, Finnmark, 2014, H. Holien	O-L-195951	OLICH1796-14	KY266863	
*Ochrolechia tartarea*	Pertusariales	NO, Sør-Trøndelag, 2014, R. Haugan	O-L-196041	OLICH3795-17	MK812341	
*Ochrolechia upsaliensis*	Pertusariales	NO, Finnmark, 2014, H. Holien	O-L-195967	OLICH1812-14	KY266960	
*Ochrolechia upsaliensis*	Pertusariales	NO, Oppland, 2013, J.T. Klepsland	O-L-198354	OLICH2376-16	MK811642	
*Opegrapha vermicellifera*	Arthoniales	NO, Hordaland, 2016, J.T. Klepsland	O-L-206757	OLICH3476-17	MK811737	
*Ophioparma lapponica*	Umbilicariales	NO, Finnmark, 2014, T.B. Wheeler	O-L-195838	OLICH1683-14	KY266901	
*Ophioparma ventosa*	Umbilicariales	FI, Inarin Lappi, 2014, E. Timdal	O-L-195498	OLICH1503-14	MK812137	
*Ophioparma ventosa*	Umbilicariales	NO, Finnmark, 2014, E. Timdal	O-L-195543	OLICH1512-14	KY266864	
*Ophioparma ventosa*	Umbilicariales	NO, Finnmark, 2014, E. Timdal	O-L-195574	OLICH1525-14	MK812332	
*Ophioparma ventosa*	Umbilicariales	NO, Oppland, 2015, E. Timdal	O-L-201417	OLICH3307-16	MK811904	
*Ophioparma ventosa*	Umbilicariales	NO, Troms, 2000, K. Lerfall	TROM_L_565529	OLICH3550-17	MK811740	
*Ophioparma ventosa*	Umbilicariales	NO, Hedmark, 2013, E. Timdal	O-L-184520	OLICH701-13	MK811771	
*Orphniospora moriopsis*	Umbilicariales	NO, Finnmark, 2014, H. Holien	O-L-195716	OLICH1588-14	KY266869	
*Pannaria conoplea*	Peltigerales	NO, Sør-Trøndelag, 2014, E. Timdal	O-L-196369	OLICH1928-15	MK811685	
*Pannaria conoplea*	Peltigerales	NO, Sogn og Fjordane, 2012, T.H. Hofton & T. Høitomt	O-L-194474	OLICH2379-16	MK812100	
*Pannaria conoplea*	Peltigerales	NO, Hedmark, 2011, R. Haugan	O-L-173219	OLICH2380-16	MK812203	
*Pannaria conoplea*	Peltigerales	NO, Hedmark, 2011, R. Haugan	O-L-173231	OLICH2382-16	MK812521	
*Pannaria conoplea*	Peltigerales	NO, Hedmark, 2011, R. Haugan	O-L-173220	OLICH2383-16	MK811788	
*Pannaria conoplea*	Peltigerales	NO, Telemark, 2015, S. Rui & E. Timdal	O-L-200927	OLICH2553-16	MK812604	
*Pannaria conoplea*	Peltigerales	NO, Nord-Trøndelag, 2015, M. Bendiksby et al.	O-L-201274	OLICH2776-16	MK812480	
*Pannaria hookeri*	Peltigerales	NO, Nordland, 2013, J.T. Klepsland	O-L-198018	OLICH3882-17	MK812291	
*Pannaria rubiginosa*	Peltigerales	NO, Nordland, 2004, A. Elvebakk	TROM_L_1340509	OLICH3563-17	MK812061	
*Pannaria rubiginosa*	Peltigerales	NO, Nord-Trøndelag, 2015, R. Haugan	O-L-201270	OLICH3846-17	MK812317	
*Parmelia ernstiae*	Lecanorales	NO, Nord-Trøndelag, 2015, M. Bendiksby et al.	O-L-201279	OLICH2777-16	MK567160	atranorin, lichesterinic acid, lobaric acid, protolichesterinic acid, salazinic acid
*Parmelia ernstiae*	Lecanorales	NO, Nord-Trøndelag, 2015, R. Haugan	O-L-201277	OLICH3848-17	MK567162	atranorin, lichesterinic acid, lobaric acid, protolichesterinic acid, salazinic acid
*Parmelia fraudans*	Lecanorales	NO, Sør-Trøndelag, 2014, E. Timdal	O-L-196374	OLICH1933-15	MK812511	
*Parmelia omphalodes*	Lecanorales	NO, Finnmark, 2014, H. Holien	O-L-195947	OLICH1792-14	KY266894	
*Parmelia saxatilis*	Lecanorales	NO, Sør-Trøndelag, 2014, R. Haugan	O-L-195994	OLICH1839-14	MK567163	atranorin, lobaric acid, salazinic acid
*Parmelia saxatilis*	Lecanorales	NO, Buskerud, 2013, S. Rui & E. Timdal	O-L-184724	OLICH940-13	MK567164	atranorin, lobaric acid, salazinic acid
*Parmelia serrana*	Lecanorales	NO, Sør-Trøndelag, 2014, R. Haugan	O-L-195995	OLICH1840-14	MK567161	atranorin, lichesterinic acid, lobaric acid, protolichesterinic acid, salazinic acid
*Parmelia serrana*	Lecanorales	NO, Vestfold, 2016, S. Rui & E. Timdal	O-L-205007	OLICH4004-17	MK567159	atranorin, lichesterinic acid, lobaric acid, protolichesterinic acid, salazinic acid
*Parmelia sulcata*	Lecanorales	NO, Telemark, 2014, E. Timdal	O-L-195789	OLICH1661-14	MK811938	
*Parmelia sulcata*	Lecanorales	NO, Sør-Trøndelag, 2014, R. Haugan	O-L-196000	OLICH1845-14	MK812088	
*Parmelia sulcata*	Lecanorales	NO, Oslo, 2014, M. Bendiksby et al.	O-L-196057	OLICH1873-14	MK812065	
*Parmelia sulcata*	Lecanorales	NO, Akershus, 2014, S. Rui & E. Timdal	O-L-196331	OLICH2261-15	MK812499	
*Parmelia sulcata*	Lecanorales	NO, Buskerud, 2013, S. Rui & E. Timdal	O-L-184694	OLICH878-13	MK812475	
*Parmeliella parvula*	Peltigerales	NO, Nord-Trøndelag, 2013, J.T. Klepsland	O-L-197955	OLICH2107-15	MK812304	
*Parmeliella triptophylla*	Peltigerales	NO, Telemark, 2015, E. Timdal	O-L-207999	OLICH3873-17	MK812457	
*Parmelina pastillifera*	Lecanorales	NO, Rogaland, 2013, J.T. Klepsland	O-L-198298	OLICH2377-16	MK812633	
*Parmelina pastillifera*	Lecanorales	NO, Hordaland, 2006, H. Fjeldstad & G. Gaarder	O-L-202999	OLICH3231-16	MK812400	
*Parmelina pastillifera*	Lecanorales	NO, Rogaland, 2016, S. Rui & E. Timdal	O-L-205003	OLICH4005-17	MK812564	
*Parmelina pastillifera*	Lecanorales	NO, Rogaland, 2016, S. Rui & E. Timdal	O-L-204989	OLICH4006-17	MK812554	
*Parmelina pastillifera*	Lecanorales	NO, Rogaland, 2016, S. Rui & E. Timdal	O-L-205000	OLICH4007-17	MK812365	
*Parmelina tiliacea*	Lecanorales	NO, Akershus, 2014, E. Timdal	O-L-199984	OLICH1958-15	MK812323	
*Parmelina tiliacea*	Lecanorales	NO, Vestfold, 2014, S. Rui & E. Timdal	O-L-196287	OLICH2217-15	MK812693	
*Parmelina tiliacea*	Lecanorales	NO, Oslo, 2014, E. Timdal	O-L-198924	OLICH2477-16	MK811628	
*Parmelina tiliacea*	Lecanorales	SE, Södermanland, 2015, S. Kistenich & E. Timdal	O-L-200873	OLICH2525-16	MK812259	
*Parmelina tiliacea*	Lecanorales	NO, Akershus, 2015, S. Rui & E. Timdal	O-L-200818	OLICH2589-16	MK812562	
*Parmelina tiliacea*	Lecanorales	NO, Oppland, 2015, E. Timdal	O-L-201334	OLICH2718-16	MK811862	
*Parmelina tiliacea*	Lecanorales	NO, Oslo, 2016, S. Rui & E. Timdal	O-L-203344	OLICH3314-16	MK812472	
*Parmelina tiliacea*	Lecanorales	NO, Rogaland, 2015, T. Tønsberg & E. Nygaard	BG-L-98117	OLICH3696-17	MK812477	
*Parmeliopsis ambigua*	Lecanorales	NO, Finnmark, 2014, R. Haugan	O-L-195902	OLICH1747-14	KY266965	
*Parmeliopsis ambigua*	Lecanorales	NO, Finnmark, 2014, E. Timdal	O-L-195934	OLICH1779-14	KY266851	
*Parmeliopsis ambigua*	Lecanorales	NO, Finnmark, 2014, H. Holien	O-L-195990	OLICH1835-14	KY266850	
*Parmeliopsis ambigua*	Lecanorales	NO, Buskerud, 2013, S. Rui & E. Timdal	O-L-184695	OLICH879-13	MK811727	
*Parmeliopsis hyperopta*	Lecanorales	NO, Finnmark, 2014, H. Holien	O-L-195989	OLICH1834-14	KY266853	
*Parmeliopsis hyperopta*	Lecanorales	NO, Buskerud, 2013, S. Rui & E. Timdal	O-L-184696	OLICH880-13	MK812515	
*Parmotrema crinitum*	Lecanorales	NO, Vest-Agder, 2013, J.T. Klepsland	O-L-197835	OLICH2109-15	MK812284	
*Parmotrema crinitum*	Lecanorales	NO, Vest-Agder, 2013, J.T. Klepsland	O-L-197840	OLICH2110-15	MK811794	
*Parmotrema perlatum*	Lecanorales	NO, Rogaland, 2014, J.T. Klepsland	O-L-200600	OLICH2651-16	MK812450	
*Parmotrema perlatum*	Lecanorales	NO, Rogaland, 2014, J.T. Klepsland	O-L-200643	OLICH4358-17	MK812630	
*Pectenia cyanoloma*	Peltigerales	NO, Nord-Trøndelag, 2013, H. Holien	O-L-193006	OLICH1477-14	MK811927	
*Pectenia cyanoloma*	Peltigerales	NO, Sogn og Fjordane, 2017, O. Olsen	O-L-207808	OLICH4320-17	MK812029	
*Peltigera aphthosa*	Peltigerales	NO, Finnmark, 2014, E. Timdal	O-L-195726	OLICH1598-14	KY266857	
*Peltigera aphthosa*	Peltigerales	NO, Buskerud, 2014, S. Rui & E. Timdal	O-L-200017	OLICH1991-15	MK811726	
*Peltigera aphthosa*	Peltigerales	NO, Buskerud, 2013, S. Rui & E. Timdal	O-L-184749	OLICH2048-15	MK812596	
*Peltigera aphthosa*	Peltigerales	NO, Sør-Trøndelag, 2014, J.T. Klepsland	O-L-200534	OLICH2611-16	MK811760	
*Peltigera aphthosa*	Peltigerales	NO, Telemark, 2015, E. Timdal	O-L-208000	OLICH3874-17	MK811699	
*Peltigera aphthosa*	Peltigerales	NO, Buskerud, 2016, M. Bendiksby & E. Timdal	O-L-208028	OLICH4256-17	MK811816	
*Peltigera britannica*	Peltigerales	NO, Nord-Trøndelag, 2015, E. Timdal et al.	O-L-201329	OLICH2689-16	MK812626	
*Peltigera canina*	Peltigerales	NO, Sør-Trøndelag, 2014, J.T. Klepsland	O-L-200554	OLICH2610-16	MK811916	
*Peltigera canina*	Peltigerales	NO, Østfold, 2012, K.A. Lye	O-L-206918	OLICH4134-17	MK812107	
*Peltigera canina*	Peltigerales	NO, Østfold, 2012, K.A. Lye	O-L-206873	OLICH4135-17	MK811752	
*Peltigera collina*	Peltigerales	NO, Sør-Trøndelag, 2014, E. Timdal	O-L-196371	OLICH1930-15	MK812439	
*Peltigera collina*	Peltigerales	SE, Södermanland, 2015, S. Kistenich & E. Timdal	O-L-200865	OLICH2517-16	MK811975	
*Peltigera collina*	Peltigerales	NO, Telemark, 2015, S. Rui & E. Timdal	O-L-200926	OLICH2540-16	MK812019	
*Peltigera collina*	Peltigerales	NO, Nord-Trøndelag, 2015, R. Haugan	O-L-201268	OLICH3845-17	MK811743	
*Peltigera degenii*	Peltigerales	NO, Telemark, 2014, E. Timdal	O-L-196276	OLICH1950-15	MK812238	
*Peltigera degenii*	Peltigerales	NO, Akershus, 2014, S. Rui & E. Timdal	O-L-196335	OLICH2273-15	MK812333	
*Peltigera degenii*	Peltigerales	NO, Buskerud, 2016, S. Rui & E. Timdal	O-L-208031	OLICH4259-17	MK812618	
*Peltigera didactyla*	Peltigerales	NO, Telemark, 2015, E. Timdal	O-L-208003	OLICH3877-17	MK811844	
*Peltigera elisabethae*	Peltigerales	NO, Buskerud, 2014, E. Timdal	O-L-196071	OLICH1887-14	MK811869	
*Peltigera extenuata*	Peltigerales	NO, Sør-Trøndelag, 2014, E. Timdal	O-L-196343	OLICH1903-15	MK811873	
*Peltigera extenuata*	Peltigerales	NO, Oslo, 2014, E. Timdal	O-L-200007	OLICH1981-15	MK811908	
*Peltigera extenuata*	Peltigerales	NO, Buskerud, 2014, S. Rui & E. Timdal	O-L-200018	OLICH1992-15	MK811654	
*Peltigera horizontalis*	Peltigerales	NO, Buskerud, 2013, S. Rui & E. Timdal	O-L-184750	OLICH2083-15	MK811925	
*Peltigera horizontalis*	Peltigerales	NO, Hedmark, 2014, R. Haugan	O-L-198894	OLICH2391-16	MK812678	
*Peltigera horizontalis*	Peltigerales	SE, Södermanland, 2015, S. Kistenich & E. Timdal	O-L-200862	OLICH2514-16	MK811673	
*Peltigera horizontalis*	Peltigerales	NO, Østfold, 2016, S. Rui & E. Timdal	O-L-208021	OLICH4193-17	MK812656	
*Peltigera hymenina*	Peltigerales	NO, Buskerud, 2014, S. Rui & E. Timdal	O-L-200019	OLICH1993-15	MK812274	
*Peltigera hymenina*	Peltigerales	NO, Oslo, 2014, E. Timdal	O-L-198933	OLICH2485-16	MK811966	
*Peltigera hymenina*	Peltigerales	NO, Vestfold, 2015, E. Timdal	O-L-198944	OLICH2495-16	MK812144	
*Peltigera hymenina*	Peltigerales	NO, Nord-Trøndelag, 2015, E. Timdal et al.	O-L-201328	OLICH2688-16	MK811735	
*Peltigera lepidophora*	Peltigerales	NO, Telemark, 2015, E. Timdal & S. Rui	O-L-200874	OLICH3835-17	MK812313	
*Peltigera lepidophora*	Peltigerales	NO, Sør-Trøndelag, 2013, E. Timdal	O-L-184530	OLICH732-13	MK812625	
*Peltigera leucophlebia*	Peltigerales	NO, Sør-Trøndelag, 2014, E. Timdal	O-L-196342	OLICH1902-15	MK812292	
*Peltigera leucophlebia*	Peltigerales	NO, Buskerud, 2014, S. Rui & E. Timdal	O-L-200020	OLICH1994-15	MK811981	
*Peltigera leucophlebia*	Peltigerales	NO, Telemark, 2015, S. Rui & E. Timdal	O-L-200929	OLICH2555-16	MK811680	
*Peltigera leucophlebia*	Peltigerales	NO, Nord-Trøndelag, 2015, E. Timdal et al.	O-L-201315	OLICH2684-16	MK812123	
*Peltigera leucophlebia*	Peltigerales	NO, Svalbard, 2015, S. Rui	O-L-203280	OLICH2699-16	MK811857	
*Peltigera leucophlebia*	Peltigerales	NO, Sør-Trøndelag, 2014, R. Haugan	O-L-196030	OLICH3807-17	MK812241	
*Peltigera leucophlebia*	Peltigerales	NO, Oppland, 2015, E. Timdal	O-L-201400	OLICH4181-17	MK812102	
*Peltigera malacea*	Peltigerales	NO, Finnmark, 2014, E. Timdal	O-L-195725	OLICH1597-14	KY266940	
*Peltigera malacea*	Peltigerales	NO, Finnmark, 2014, R. Haugan	O-L-195848	OLICH1693-14	KY266836	
*Peltigera malacea*	Peltigerales	NO, Sør-Trøndelag, 2014, E. Timdal	O-L-196362	OLICH1921-15	MK811739	
*Peltigera malacea*	Peltigerales	NO, Oslo, 2014, E. Timdal	O-L-200006	OLICH1980-15	MK812014	
*Peltigera malacea*	Peltigerales	NO, Buskerud, 2013, S. Rui & E. Timdal	O-L-184751	OLICH2085-15	MK811998	
*Peltigera malacea*	Peltigerales	NO, Nord-Trøndelag, 2015, E. Timdal et al.	O-L-201331	OLICH2690-16	MK811990	
*Peltigera membranacea*	Peltigerales	NO, Sør-Trøndelag, 2014, R. Haugan	O-L-195991	OLICH1836-14	MK811987	
*Peltigera membranacea*	Peltigerales	NO, Buskerud, 2014, S. Rui & E. Timdal	O-L-200024	OLICH2472-16	MK812374	
*Peltigera neckeri*	Peltigerales	NO, Finnmark, 2014, E. Timdal	O-L-195753	OLICH1625-14	KY266937	
*Peltigera neckeri*	Peltigerales	NO, Finnmark, 2014, E. Timdal	O-L-195932	OLICH1777-14	MK811778	
*Peltigera neckeri*	Peltigerales	NO, Telemark, 2015, S. Rui & E. Timdal	O-L-200910	OLICH2582-16	MK811968	
*Peltigera neocanina*	Peltigerales	NO, Buskerud, 2014, R. Haugan & E. Timdal	O-L-196062	OLICH1878-14	MK812413	
*Peltigera neopolydactyla*	Peltigerales	NO, Telemark, 2014, E. Timdal	O-L-196266	OLICH1941-15	MK812243	
*Peltigera neopolydactyla*	Peltigerales	NO, Telemark, 2014, E. Timdal	O-L-196277	OLICH1946-15	MK812022	
*Peltigera neopolydactyla*	Peltigerales	NO, Oslo, 2014, E. Timdal	O-L-199994	OLICH1968-15	MK812548	
*Peltigera neopolydactyla*	Peltigerales	NO, Buskerud, 2013, S. Rui & E. Timdal	O-L-184753	OLICH2084-15	MK811653	
*Peltigera neopolydactyla*	Peltigerales	NO, Buskerud, 2014, S. Rui & E. Timdal	O-L-200025	OLICH2473-16	MK812286	
*Peltigera neopolydactyla*	Peltigerales	NO, Nord-Trøndelag, 2015, E. Timdal et al.	O-L-201332	OLICH2691-16	MK812172	
*Peltigera polydactylon*	Peltigerales	NO, Telemark, 2014, E. Timdal	O-L-196264	OLICH1939-15	MK811815	
*Peltigera polydactylon*	Peltigerales	NO, Telemark, 2014, E. Timdal	O-L-195820	OLICH2192-15	MK812112	
*Peltigera polydactylon*	Peltigerales	NO, Buskerud, 2016, S. Rui & E. Timdal	O-L-206899	OLICH4136-17	MK812583	
*Peltigera praetextata*	Peltigerales	NO, Buskerud, 2013, S. Rui & E. Timdal	O-L-184752	OLICH2044-15	MK811692	
*Peltigera praetextata*	Peltigerales	NO, Oslo, 2014, E. Timdal	O-L-195837	OLICH2209-15	MK811856	
*Peltigera praetextata*	Peltigerales	NO, Akershus, 2009, T. Starholm	O-L-201769	OLICH3880-17	MK812051	
*Peltigera praetextata*	Peltigerales	NO, Østfold, 2016, S. Rui & E. Timdal	O-L-208022	OLICH4194-17	MK812578	
*Peltigera retifoveata*	Peltigerales	NO, Sør-Trøndelag, 2014, J.T. Klepsland	O-L-200533	OLICH2605-16	MK811996	
*Peltigera rufescens*	Peltigerales	NO, Oslo, 2014, S. Rui & E. Timdal	O-L-196072	OLICH1888-14	MK812663	
*Peltigera rufescens*	Peltigerales	NO, Sør-Trøndelag, 2014, E. Timdal	O-L-196341	OLICH1901-15	MK811858	
*Peltigera rufescens*	Peltigerales	NO, Akershus, 2014, E. Timdal	O-L-199989	OLICH1963-15	MK812224	
*Peltigera rufescens*	Peltigerales	NO, Oppland, 2015, E. Timdal	O-L-201347	OLICH2733-16	MK812696	
*Peltigera scabrosa*	Peltigerales	NO, Finnmark, 2014, E. Timdal	O-L-195729	OLICH1601-14	KY266948	
*Peltigera scabrosa*	Peltigerales	NO, Finnmark, 2014, E. Timdal	O-L-195765	OLICH1637-14	KY266970	
*Peltigera scabrosa*	Peltigerales	NO, Telemark, 2015, E. Timdal	O-L-207997	OLICH3871-17	MK812044	
*Peltigera scabrosella*	Peltigerales	NO, Nord-Trøndelag, 2015, M. Bendiksby et al.	O-L-201255	OLICH2763-16	MK812406	
*Peltigera scabrosella*	Peltigerales	NO, Telemark, 2015, E. Timdal	O-L-207998	OLICH3872-17	MK812463	
*Peltula euploca*	Lichinales	NO, Oppland, 2013, J.T. Klepsland	O-L-197859	OLICH2111-15	MK811928	
*Pertusaria alpina*	Pertusariales	NO, Nordland, 2014, J.T. Klepsland	O-L-200312	OLICH3141-16	MK811905	
*Pertusaria alpina*	Pertusariales	NO, Nord-Trøndelag, 2013, J.T. Klepsland	O-L-197933	OLICH4038-17	MK811785	
*Pertusaria aspergilla*	Pertusariales	NO, Sør-Trøndelag, 2015, H. Holien	TRH-L-16775	OLICH3686-17	MK812282	
*Pertusaria aspergilla*	Pertusariales	NO, Nordland, 2015, T. Tønsberg	BG-L-99158	OLICH3716-17	MK812384	
*Pertusaria aspergilla*	Pertusariales	NO, Østfold, 2015, R. Haugan	O-L-204101	OLICH3929-17	MK812496	fumarprotocetraric acid
*Pertusaria carneopallida*	Pertusariales	NO, Nordland, 2013, J.T. Klepsland	O-L-198006	OLICH4039-17	MK812218	
*Pertusaria excludens*	Pertusariales	NO, Nord-Trøndelag, 2015, R. Haugan	O-L-203909	OLICH3927-17	MK811670	norstictic acid
*Pertusaria excludens*	Pertusariales	NO, Sør-Trøndelag, 2014, R. Haugan	O-L-203523	OLICH3935-17	MK812675	norstictic acid
*Pertusaria flavicans*	Pertusariales	NO, Møre og Romsdal, 2015, T. Tønsberg	BG-L-99071	OLICH3777-17	MK812409	
*Pertusaria flavida*	Pertusariales	SE, Södermanland, 2015, S. Kistenich & E. Timdal	O-L-200860	OLICH2512-16	MK811913	
*Pertusaria leioplaca*	Pertusariales	NO, Troms, 2000, S. Werth	TROM_L_565683	OLICH3551-17	MK811922	
*Pertusaria pertusa*	Pertusariales	NO, Vestfold, 2015, E. Timdal	O-L-198948	OLICH2502-16	MK812037	
*Phaeophyscia endococcina*	Caliciales	NO, Finnmark, 2014, R. Haugan	O-L-198738	OLICH2400-16	MK812217	
*Phaeophyscia kairamoi*	Caliciales	NO, Sør-Trøndelag, 2014, E. Timdal	O-L-196355	OLICH1914-15	MK811724	
*Phaeophyscia orbicularis*	Caliciales	NO, Finnmark, 2014, E. Timdal	O-L-195734	OLICH1606-14	KY266882	
*Phaeophyscia orbicularis*	Caliciales	NO, Sør-Trøndelag, 2014, R. Haugan	O-L-196004	OLICH1849-14	MK811854	
*Phaeophyscia orbicularis*	Caliciales	NO, Vestfold, 2014, S. Rui & E. Timdal	O-L-196291	OLICH2221-15	MK811807	
*Phaeophyscia orbicularis*	Caliciales	NO, Akershus, 2014, S. Rui & E. Timdal	O-L-196330	OLICH2259-15	MK812383	
*Phaeophyscia orbicularis*	Caliciales	NO, Hedmark, 2014, R. Haugan	O-L-207984	OLICH3819-17	MK812363	
*Phaeophyscia sciastra*	Caliciales	NO, Finnmark, 2014, E. Timdal	O-L-195721	OLICH1593-14	KY266837	
*Phaeophyscia sciastra*	Caliciales	NO, Sør-Trøndelag, 2014, E. Timdal	O-L-196352	OLICH1911-15	MK812372	
*Phlyctis agelaea*	Ostropales	NO, Hordaland, 2015, J.T. Klepsland	O-L-206746	OLICH3465-17	MK812009	
*Phlyctis argena*	Ostropales	NO, Nordland, 2015, T. Tønsberg & A. Botnen	BG-L-98987	OLICH3760-17	MK811759	
*Phlyctis argena*	Ostropales	NO, Nordland, 2015, T. Tønsberg & A. Botnen	BG-L-99194	OLICH3761-17	MK811753	
*Phlyctis argena*	Ostropales	NO, Buskerud, 2014, T. Tønsberg	BG-L-99532	OLICH3786-17	MK811872	
*Physcia adscendens*	Caliciales	NO, Sør-Trøndelag, 2014, R. Haugan	O-L-196005	OLICH1850-14	MK811630	
*Physcia adscendens*	Caliciales	NO, Vestfold, 2015, E. Timdal	O-L-198947	OLICH2501-16	MK812201	
*Physcia aipolia*	Caliciales	NO, Telemark, 2014, E. Timdal	O-L-195783	OLICH1655-14	MK812601	
*Physcia aipolia*	Caliciales	NO, Sør-Trøndelag, 2014, R. Haugan	O-L-195992	OLICH1837-14	MK811770	
*Physcia aipolia*	Caliciales	NO, Telemark, 2015, S. Rui & E. Timdal	O-L-200890	OLICH2528-16	MK811989	
*Physcia aipolia*	Caliciales	NO, Oslo, 2014, J.T. Klepsland	O-L-200142	OLICH2609-16	MK812087	
*Physcia aipolia*	Caliciales	NO, Østfold, 2012, K.A. Lye	O-L-206860	OLICH4138-17	MK811719	
*Physcia alnophila*	Caliciales	NO, Nord-Trøndelag, 2015, M. Bendiksby et al.	O-L-201269	OLICH2773-16	MK812315	
*Physcia caesia*	Caliciales	NO, Telemark, 2014, E. Timdal	O-L-195792	OLICH1664-14	MK811883	
*Physcia caesia*	Caliciales	NO, Oslo, 2014, M. Bendiksby et al.	O-L-196078	OLICH1894-14	MK812056	
*Physcia caesia*	Caliciales	NO, Akershus, 2014, S. Rui & E. Timdal	O-L-196333	OLICH2263-15	MK812325	
*Physcia caesia*	Caliciales	NO, Sør-Trøndelag, 2014, R. Haugan	O-L-196035	OLICH3791-17	MK812524	
*Physcia dimidiata*	Caliciales	NO, Buskerud, 2015, E. Timdal	O-L-201418	OLICH3299-16	MK812556	
*Physcia dubia*	Caliciales	NO, Oppland, 2013, J.T. Klepsland	O-L-197852	OLICH2113-15	MK812017	
*Physcia dubia*	Caliciales	NO, Oppland, 2015, E. Timdal	O-L-201373	OLICH2748-16	MK811700	
*Physcia magnussonii*	Caliciales	NO, Sogn og Fjordane, 2013, J.T. Klepsland	O-L-197861	OLICH2114-15	MK811875	
*Physcia phaea*	Caliciales	NO, Sør-Trøndelag, 2014, E. Timdal	O-L-196373	OLICH1932-15	MK811892	
*Physcia stellaris*	Caliciales	NO, Troms, 2014, E. Timdal	O-L-195769	OLICH1641-14	MK811861	
*Physcia stellaris*	Caliciales	NO, Østfold, 2012, K.A. Lye	O-L-206903	OLICH4137-17	MK812381	
*Physcia tenella*	Caliciales	NO, Finnmark, 2014, E. Timdal	O-L-195561	OLICH1519-14	KY266867	
*Physcia tenella*	Caliciales	NO, Sør-Trøndelag, 2014, R. Haugan	O-L-196007	OLICH1852-14	MK811920	
*Physcia tenella*	Caliciales	NO, Oslo, 2014, M. Bendiksby et al.	O-L-196079	OLICH1895-14	MK811798	
*Physcia tenella*	Caliciales	NO, Vestfold, 2014, S. Rui & E. Timdal	O-L-196294	OLICH2224-15	MK811848	
*Physcia tenella*	Caliciales	NO, Rogaland, 2013, J.T. Klepsland	O-L-198307	OLICH2357-15	MK811950	
*Physcia tenella*	Caliciales	NO, Sør-Trøndelag, 2014, R. Haugan	O-L-196034	OLICH3798-17	MK812588	
*Physconia distorta*	Caliciales	NO, Akershus, 2014, E. Timdal	O-L-199983	OLICH1957-15	MK811745	
*Physconia distorta*	Caliciales	NO, Vestfold, 2014, S. Rui & E. Timdal	O-L-196290	OLICH2220-15	MK812071	
*Physconia enteroxantha*	Caliciales	NO, Oslo, 2014, R. Haugan et al.	O-L-196058	OLICH1874-14	MK811840	
*Physconia enteroxantha*	Caliciales	NO, Akershus, 2014, E. Timdal	O-L-199986	OLICH1960-15	MK811812	
*Physconia enteroxantha*	Caliciales	NO, Vestfold, 2014, S. Rui & E. Timdal	O-L-196293	OLICH2223-15	MK811936	
*Physconia enteroxantha*	Caliciales	NO, Akershus, 2014, S. Rui & E. Timdal	O-L-196328	OLICH2258-15	MK812142	
*Physconia muscigena*	Caliciales	NO, Finnmark, 2014, E. Timdal	O-L-195722	OLICH1594-14	KY266849	
*Physconia muscigena*	Caliciales	NO, Finnmark, 2014, E. Timdal	O-L-195920	OLICH1765-14	KY266925	
*Physconia muscigena*	Caliciales	NO, Sør-Trøndelag, 2014, E. Timdal	O-L-196344	OLICH1904-15	MK811781	
*Pilophorus dovrensis*	Lecanorales	NO, Troms, 2003, E. Timdal	O-L-123083	OLICH211-13	MK812599	
*Placynthiella oligotropha*	Trapeliales	NO, Oppland, 2012, E. Timdal	O-L-182032	OLICH1176-14	MK811853	
*Placynthiella oligotropha*	Trapeliales	NO, Oppland, 2011, M. Bendiksby et al.	O-L-175738	OLICH1177-14	MK812378	
*Platismatia glauca*	Lecanorales	NO, Troms, 2014, E. Timdal	O-L-195649	OLICH1561-14	MK812715	
*Platismatia glauca*	Lecanorales	NO, Sør-Trøndelag, 2014, R. Haugan	O-L-195993	OLICH1838-14	MK812351	
*Platismatia glauca*	Lecanorales	NO, Telemark, 2015, S. Rui & E. Timdal	O-L-200882	OLICH2564-16	MK812456	
*Platismatia glauca*	Lecanorales	NO, Buskerud, 2013, S. Rui & E. Timdal	O-L-184697	OLICH881-13	MK812210	
*Platismatia norvegica*	Lecanorales	NO, Nord-Trøndelag, 2015, M. Bendiksby et al.	O-L-201267	OLICH2772-16	MK812616	
*Platismatia norvegica*	Lecanorales	NO, Akershus, 2017, S. Rui & E. Timdal	O-L-206864	OLICH4109-17	MK812705	
*Pleopsidium chlorophanum*	Acarosporales	NO, Troms, 2014, E. Timdal	O-L-195643	OLICH1555-14	MK811850	
*Pleopsidium chlorophanum*	Acarosporales	NO, Finnmark, 2014, E. Timdal	O-L-195940	OLICH1785-14	KY266860	
*Pleopsidium chlorophanum*	Acarosporales	NO, Nord-Trøndelag, 2015, M. Bendiksby et al.	O-L-201252	OLICH2759-16	MK812117	
*Pleopsidium chlorophanum*	Acarosporales	NO, Sør-Trøndelag, 2017, E. Timdal	O-L-208244	OLICH4322-17	MK812354	
*Pleopsidium chlorophanum*	Acarosporales	NO, Telemark, 2013, E. Timdal	O-L-184414	OLICH695-13	MK812213	
*Pleopsidium flavum*	Acarosporales	NO, Sør-Trøndelag, 2014, J.T. Klepsland	O-L-200545	OLICH2624-16	MK811960	
*Pleopsidium flavum*	Acarosporales	NO, Telemark, 2013, E. Timdal	O-L-184415	OLICH696-13	MK812038	
*Pleopsidium flavum*	Acarosporales	NO, Hordaland, 2013, E. Timdal	O-L-184439	OLICH697-13	MK811877	
*Pleurosticta acetabulum*	Lecanorales	NO, Akershus, 2014, E. Timdal	O-L-199985	OLICH1959-15	MK812572	
*Pleurosticta acetabulum*	Lecanorales	NO, Vestfold, 2015, E. Timdal	O-L-198935	OLICH2488-16	MK812152	
*Pleurosticta acetabulum*	Lecanorales	SE, Södermanland, 2015, S. Kistenich & E. Timdal	O-L-200869	OLICH2521-16	MK811775	
*Pleurosticta acetabulum*	Lecanorales	NO, Rogaland, 2015, T. Tønsberg & E. Nygaard	BG-L-98170	OLICH3700-17	MK811799	
*Pleurosticta acetabulum*	Lecanorales	NO, Østfold, 2002, B. P. Løfall	O-L-108808	OLICH4128-17	MK812479	
*Polycauliona candelaria*	Teloschistales	NO, Akershus, 2014, E. Timdal	O-L-199981	OLICH1955-15	MK811915	
*Polycauliona candelaria*	Teloschistales	NO, Buskerud, 2014, S. Rui & E. Timdal	O-L-196326	OLICH2256-15	MK812066	
*Polycauliona candelaria*	Teloschistales	NO, Sør-Trøndelag, 2014, E. Timdal	O-L-196340	OLICH2278-15	MK811959	
*Polychidium muscicola*	Peltigerales	NO, Nord-Trøndelag, 2015, M. Bendiksby et al.	O-L-201293	OLICH2795-16	MK812283	
*Protoblastenia rupestris*	Lecanorales	NO, Finnmark, 2014, A. Millanes	O-L-195859	OLICH1704-14	KY266981	
*Protomicarea limosa*	Lecanorales	NO, Hedmark, 2012, R. Haugan	O-L-182289	OLICH1084-14	MK811666	
*Protomicarea limosa*	Lecanorales	NO, Hedmark, 2004, R. Haugan	O-L-131761	OLICH1085-14	MK812532	
*Protomicarea limosa*	Lecanorales	NO, Nord-Trøndelag, 2009, J.T. Klepsland	O-L-165102	OLICH1086-14	MK812181	
*Protomicarea limosa*	Lecanorales	NO, Oppland, 2012, E. Timdal	O-L-182031	OLICH1140-14	MK812033	
*Protomicarea limosa*	Lecanorales	NO, Nordland, 2010, T. Tønsberg	BG-L-92492	OLICH601-13	MK812586	
*Protomicarea limosa*	Lecanorales	NO, Oppland, 2013, M. Bendiksby et al.	O-L-184281	OLICH821-13	MK812176	
*Protopannaria pezizoides*	Peltigerales	NO, Finnmark, 2014, A. Guillermo	O-L-195697	OLICH1569-14	KY266840	
*Protopannaria pezizoides*	Peltigerales	NO, Finnmark, 2014, E. Timdal	O-L-195890	OLICH1735-14	KY266909	
*Protopannaria pezizoides*	Peltigerales	NO, Finnmark, 2014, H. Holien	O-L-195970	OLICH1815-14	KY266886	
*Protopannaria pezizoides*	Peltigerales	NO, Sør-Trøndelag, 2014, E. Timdal	O-L-196347	OLICH1907-15	MK811767	
*Protopannaria pezizoides*	Peltigerales	NO, Nord-Trøndelag, 2013, J.T. Klepsland	O-L-197950	OLICH2115-15	MK812467	
*Protopannaria pezizoides*	Peltigerales	NO, Svalbard, 2015, S. Rui	O-L-203297	OLICH2706-16	MK811974	
*Protoparmelia badia*	Lecanorales	NO, Finnmark, 2014, E. Timdal	O-L-195568	OLICH1522-14	KY266939	
*Protoparmelia badia*	Lecanorales	NO, Finnmark, 2014, A. Launis	O-L-195839	OLICH1684-14	KY266945	
*Pseudephebe minuscula*	Lecanorales	NO, Sør-Trøndelag, 2014, E. Timdal	O-L-196378	OLICH1937-15	MK811997	
*Pseudephebe minuscula*	Lecanorales	NO, Oppland, 2013, M. Bendiksby et al.	O-L-184305	OLICH736-13	MK812067	
*Pseudephebe pubescens*	Lecanorales	NO, Troms, 2014, E. Timdal	O-L-195619	OLICH1548-14	MK812357	
*Pseudephebe pubescens*	Lecanorales	NO, Finnmark, 2014, E. Timdal	O-L-195849	OLICH1694-14	KY266938	
*Pseudephebe pubescens*	Lecanorales	NO, Finnmark, 2014, R. Haugan	O-L-195986	OLICH1831-14	KY266914	
*Pseudephebe pubescens*	Lecanorales	NO, Oppland, 2013, M. Bendiksby et al.	O-L-184396	OLICH694-13	MK812150	
*Pseudephebe pubescens*	Lecanorales	NO, Oppland, 2013, M. Bendiksby et al.	O-L-184306	OLICH735-13	MK811888	
*Pseudephebe pubescens*	Lecanorales	NO, Buskerud, 2013, S. Rui & E. Timdal	O-L-184725	OLICH941-13	MK812652	
*Pseudevernia furfuracea*	Lecanorales	NO, Sør-Trøndelag, 2014, R. Haugan	O-L-195999	OLICH1844-14	MK812319	
*Pseudevernia furfuracea*	Lecanorales	NO, Hedmark, 2016, E. Timdal	O-L-201522	OLICH3984-17	MK812161	
*Pseudevernia furfuracea*	Lecanorales	NO, Aust-Agder, 2016, S. Rui & E. Timdal	O-L-204977	OLICH4008-17	MK812508	
*Pseudevernia furfuracea*	Lecanorales	NO, Telemark, 2016, S. Rui & E. Timdal	O-L-205006	OLICH4009-17	MK811934	
*Pseudevernia furfuracea*	Lecanorales	NO, Buskerud, 2013, S. Rui & E. Timdal	O-L-184698	OLICH882-13	MK812628	
*Pseudocyphellaria citrina*	Peltigerales	NO, Nord-Trøndelag, 2013, J.T. Klepsland	O-L-197920	OLICH2116-15	MK812391	
*Pseudocyphellaria citrina*	Peltigerales	NO, Sør-Trøndelag, 2013, J.T. Klepsland	O-L-198050	OLICH2117-15	MK811890	
*Pseudocyphellaria citrina*	Peltigerales	NO, Nord-Trøndelag, 2015, M. Bendiksby et al.	O-L-201266	OLICH2771-16	MK812527	
*Pseudocyphellaria norvegica*	Peltigerales	NO, Hordaland, 2006, H. Fjeldstad	O-L-203000	OLICH3242-16	MK812692	
*Psilolechia clavulifera*	Lecanorales	NO, Akershus, 2009, J.T. Klepsland	O-L-164653	OLICH1081-14	MK811831	
*Psilolechia leprosa*	Lecanorales	NO, Finnmark, 2014, E. Timdal	O-L-195611	OLICH1544-14	MK812516	
*Psilolechia leprosa*	Lecanorales	NO, Aust-Agder, 2015, J.T. Klepsland	O-L-204517	OLICH4229-17	MK811951	
*Psilolechia leprosa*	Lecanorales	NO, Aust-Agder, 2015, J.T. Klepsland	O-L-204519	OLICH4251-17	MK812436	
*Psilolechia lucida*	Lecanorales	SE, Närke, 2013, M. Bendiksby et al.	O-L-184487	OLICH665-13	MK811768	
*Psora decipiens*	Lecanorales	NO, Oppland, 2013, J.T. Klepsland	O-L-198348	OLICH2358-15	MK812030	
*Psora decipiens*	Lecanorales	NO, Troms, 2014, J.T. Klepsland	O-L-200362	OLICH2646-16	MK811804	
*Psora globifera*	Lecanorales	NO, Nord-Trøndelag, 2011, J.T. Klepsland	O-L-177145	OLICH1319-14	KU873929	no lichen substances
*Psora globifera*	Lecanorales	NO, Sør-Trøndelag, 2012, R. Hjelmstad	O-L-184143	OLICH1320-14	KU873932	no lichen substances
*Psora globifera*	Lecanorales	NO, Buskerud, 2014, J.T. Klepsland	O-L-200597	OLICH2645-16	MK812110	
*Psora globifera*	Lecanorales	NO, Oppland, 2011, J.T. Klepsland	O-L-183774	OLICH782-13	KU873928	no lichen substances
*Psora globifera*	Lecanorales	NO, Oppland, 2013, M. Bendiksby et al.	O-L-184327	OLICH820-13	KU873930	no lichen substances
*Psora rubiformis*	Lecanorales	NO, Oppland, 2012, H. Bratli & R. Haugan	O-L-183466	OLICH1082-14	MK811688	
*Psora rubiformis*	Lecanorales	NO, Nord-Trøndelag, 2011, J.T. Klepsland	O-L-177142	OLICH1321-14	MK812240	
*Psora rubiformis*	Lecanorales	NO, Finnmark, 2014, E. Timdal	O-L-195525	OLICH1508-14	KY266957	
*Psora rubiformis*	Lecanorales	NO, Hordaland, 2013, E. Timdal	O-L-184422	OLICH648-13	MK811706	
*Psora rubiformis*	Lecanorales	NO, Oppland, 2013, M. Bendiksby et al.	O-L-184359	OLICH649-13	MK812000	
*Psora vallesiaca*	Lecanorales	NO, Oppland, 2013, M. Bendiksby et al.	O-L-184392	OLICH650-13	KU873926	norstictic acid
*Psora vallesiaca*	Lecanorales	NO, Oppland, 2011, J.T. Klepsland	O-L-183778	OLICH780-13	KU873927	norstictic acid
*Psora vallesiaca*	Lecanorales	NO, Oppland, 2011, J.T. Klepsland	O-L-183760	OLICH781-13	KU873931	norstictic acid
*Psorinia conglomerata*	Lecanorales	NO, Oppland, 2012, H. Bratli & R. Haugan	O-L-182192	OLICH1061-14	MK812001	stictic acid
*Psoroma cinnamomeum*	Peltigerales	NO, Sør-Trøndelag, 2013, E. Timdal	O-L-184538	OLICH702-13	MK811978	
*Psoroma hypnorum*	Peltigerales	NO, Finnmark, 2014, E. Timdal	O-L-195857	OLICH1702-14	KY266971	
*Psoroma hypnorum*	Peltigerales	NO, Sør-Trøndelag, 2017, E. Timdal	O-L-208202	OLICH4325-17	MK811800	
*Psoroma hypnorum*	Peltigerales	NO, Sør-Trøndelag, 2013, E. Timdal	O-L-184537	OLICH703-13	MK811764	
*Psoronactis dilleniana*	Arthoniales	NO, Nord-Trøndelag, 2015, H. Holien	TRH-L-16932	OLICH3649-17	MK812257	
*Punctelia stictica*	Lecanorales	NO, Oppland, 2015, E. Timdal	O-L-201333	OLICH2698-16	MK812448	
*Punctelia subrudecta*	Lecanorales	NO, Rogaland, 2015, T. Tønsberg	BG-L-98118	OLICH3697-17	MK812035	
*Punctelia subrudecta*	Lecanorales	NO, Rogaland, 2014, J.T. Klepsland	O-L-200662	OLICH4106-17	MK812237	
*Pycnothelia papillaria*	Lecanorales	NO, Aust-Agder, 2006, J.T. Klepsland	O-L-151129	OLICH1364-14	MK811765	atranorin, lichesterinic acid
*Pycnothelia papillaria*	Lecanorales	NO, Oslo, 2014, E. Timdal	O-L-200001	OLICH1975-15	MK812272	
*Pycnothelia papillaria*	Lecanorales	NO, Nord-Trøndelag, 2015, M. Bendiksby et al.	O-L-201299	OLICH2797-16	MK811947	
*Pyrenula laevigata*	Pyrenulales	NO, Hordaland, 2016, J.T. Klepsland	O-L-206758	OLICH3477-17	MK812685	
*Pyrenula laevigata*	Pyrenulales	NO, Møre og Romsdal, 2016, J.T. Klepsland	O-L-206773	OLICH3492-17	MK812185	
*Pyrenula occidentalis*	Pyrenulales	NO, Møre og Romsdal, 2016, J.T. Klepsland	O-L-206777	OLICH3496-17	MK811633	
*Racodium rupestre*	Capnodiales	NO, Rogaland, 2013, J.T. Klepsland	O-L-198464	OLICH3945-17	MK812095	
*Ramalina capitata*	Lecanorales	NO, Buskerud, 2014, S. Rui & E. Timdal	O-L-200012	OLICH1986-15	MK811917	
*Ramalina capitata*	Lecanorales	NO, Buskerud, 2014, E. Timdal	O-L-195831	OLICH2203-15	MK811626	
*Ramalina capitata*	Lecanorales	NO, Buskerud, 2015, S. Rui & E. Timdal	O-L-200896	OLICH2549-16	MK812528	
*Ramalina capitata*	Lecanorales	NO, Oppland, 2013, M. Bendiksby et al.	O-L-184378	OLICH693-13	MK812398	
*Ramalina cuspidata*	Lecanorales	NO, Sør-Trøndelag, 2014, R. Haugan	O-L-196016	OLICH1861-14	MK812006	usnic acid
*Ramalina cuspidata*	Lecanorales	NO, Vest-Agder, 2013, J.T. Klepsland	O-L-197829	OLICH2118-15	MK811797	
*Ramalina farinacea*	Lecanorales	NO, Sør-Trøndelag, 2014, R. Haugan	O-L-196002	OLICH1847-14	MK812207	protocetraric acid, usnic acid
*Ramalina farinacea*	Lecanorales	NO, Akershus, 2014, E. Timdal	O-L-199987	OLICH1961-15	MK812169	protocetraric acid, usnic acid
*Ramalina farinacea*	Lecanorales	NO, Vestfold, 2014, S. Rui & E. Timdal	O-L-196286	OLICH2216-15	MK812449	protocetraric acid , usnic acid
*Ramalina fastigiata*	Lecanorales	NO, Vestfold, 2014, S. Rui & E. Timdal	O-L-196289	OLICH2219-15	MK811906	
*Ramalina fastigiata*	Lecanorales	NO, Vestfold, 2014, J.T. Klepsland	O-L-200570	OLICH2644-16	MK811841	
*Ramalina fastigiata*	Lecanorales	NO, Østfold, 2016, S. Rui & E. Timdal	O-L-208026	OLICH4197-17	MK812196	
*Ramalina fraxinea*	Lecanorales	NO, Vestfold, 2014, S. Rui & E. Timdal	O-L-196288	OLICH2218-15	MK812590	
*Ramalina fraxinea*	Lecanorales	NO, Vestfold, 2014, J.T. Klepsland	O-L-200571	OLICH4105-17	MK812489	
*Ramalina fraxinea*	Lecanorales	NO, Akershus, 2012, K.A. Lye	O-L-206861	OLICH4140-17	MK811900	
*Ramalina fraxinea*	Lecanorales	NO, Østfold, 2016, S. Rui & E. Timdal	O-L-208025	OLICH4196-17	MK812162	
*Ramalina pollinaria*	Lecanorales	NO, Sør-Trøndelag, 2014, E. Timdal	O-L-196372	OLICH1931-15	MK812166	
*Ramalina pollinaria*	Lecanorales	SE, Södermanland, 2015, S. Kistenich & E. Timdal	O-L-200850	OLICH2516-16	MK812114	
*Ramalina pollinaria*	Lecanorales	NO, Telemark, 2015, S. Rui & E. Timdal	O-L-200901	OLICH2552-16	MK811866	
*Ramalina polymorpha*	Lecanorales	NO, Finnmark, 2014, E. Timdal	O-L-195737	OLICH1609-14	KY266885	
*Ramalina polymorpha*	Lecanorales	NO, Finnmark, 2014, E. Timdal	O-L-195928	OLICH1773-14	MK811690	
*Ramalina polymorpha*	Lecanorales	NO, Buskerud, 2014, S. Rui & E. Timdal	O-L-200011	OLICH1985-15	MK811661	
*Ramalina polymorpha*	Lecanorales	NO, Buskerud, 2015, S. Rui & E. Timdal	O-L-200898	OLICH2551-16	MK812673	
*Ramalina siliquosa*	Lecanorales	NO, Sør-Trøndelag, 2014, R. Haugan	O-L-196010	OLICH1855-14	MK811720	protocetraric acid (minor), salazinic acid (major), usnic acid (trace)
*Ramalina subfarinacea*	Lecanorales	NO, Vestfold, 2014, S. Rui & E. Timdal	O-L-196284	OLICH2214-15	MK812104	norstictic acid (major), usnic acid (major)
*Ramalina subfarinacea*	Lecanorales	NO, Nordland, 2014, J.T. Klepsland	O-L-200339	OLICH2643-16	MK812227	norstictic acid (major), usnic acid (major)
*Ramboldia elabens*	Lecanorales	NO, Sør-Trøndelag, 2007, E. Timdal	O-L-158501	OLICH411-13	MK812637	
*Ramboldia elabens*	Lecanorales	NO, Hedmark, 2008, R. Haugan	O-L-160473	OLICH412-13	MK812132	
*Ramboldia elabens*	Lecanorales	NO, Oppland, 2004, T.H. Hofton	O-L-136707	OLICH413-13	MK812702	
*Ramboldia elabens*	Lecanorales	NO, Nord-Trøndelag, 2006, J.T. Klepsland	O-L-183511	OLICH472-13	MK811668	
*Ramboldia elabens*	Lecanorales	NO, Finnmark, 2011, E. Timdal	O-L-170692	OLICH473-13	MK811870	
*Ramboldia elabens*	Lecanorales	NO, Nordland, 2011, T. Tønsberg	BG-L-95299	OLICH580-13	MK812420	
*Rhizocarpon badioatrum*	Rhizocarpales	NO, Oppland, 2010, E. Timdal	O-L-163824	OLICH002-11	KU687450	no lichen substances
*Rhizocarpon badioatrum*	Rhizocarpales	NO, Buskerud, 2010, E. Timdal	O-L-163839	OLICH003-11	KU687453	no lichen substances
*Rhizocarpon bolanderi*	Rhizocarpales	NO, Aust-Agder, 2010, E. Timdal	O-L-163373	OLICH023-11	KU687451	norstictic acid (trace), stictic acid (major)
*Rhizocarpon chioneum*	Rhizocarpales	NO, Finnmark, 2014, E. Timdal	O-L-195535	OLICH1510-14	KY266951	
*Rhizocarpon copelandii*	Rhizocarpales	NO, Oppland, 2013, M. Bendiksby et al.	O-L-184329	OLICH766-13	KU687447	
*Rhizocarpon copelandii*	Rhizocarpales	NO, Hedmark, 2013, E. Timdal	O-L-184521	OLICH790-13	KU687456	norstictic acid
*Rhizocarpon copelandii*	Rhizocarpales	NO, Oppland, 2012, S. Rui & E. Timdal	O-L-180002	OLICH837-13	KU687455	norstictic acid
*Rhizocarpon geminatum*	Rhizocarpales	NO, Finnmark, 2014, E. Timdal	O-L-195564	OLICH1520-14	KY266908	
*Rhizocarpon jemtlandicum*	Rhizocarpales	NO, Telemark, 2010, E. Timdal	O-L-163364	OLICH015-11	KU687445	norstictic acid (trace), stictic acid (major)
*Rhizocarpon jemtlandicum*	Rhizocarpales	NO, Nordland, 2010, E. Timdal	O-L-163756	OLICH019-11	KU687446	norstictic acid (minor), stictic acid (major)
*Rhizocarpon jemtlandicum*	Rhizocarpales	NO, Finnmark, 2011, E. Timdal	O-L-170452	OLICH803-13	KU687460	stictic acid
*Rhizocarpon leptolepis*	Rhizocarpales	FI, Inarin Lappi, 2014, E. Timdal	O-L-195500	OLICH1504-14	KU687449	
*Rhizocarpon pycnocarpoides*	Rhizocarpales	NO, Nordland, 2012, R. Haugan	O-L-183808	OLICH511-13	KT800004	no lichen substances
*Rhizocarpon pycnocarpoides*	Rhizocarpales	NO, Nord-Trøndelag, 2012, R. Haugan	O-L-183810	OLICH512-13	KT800005	no lichen substances
*Rhizocarpon pycnocarpoides*	Rhizocarpales	NO, Oppland, 2012, S. Rui & E. Timdal	O-L-179903	OLICH513-13	KT800002	
*Rhizocarpon pycnocarpoides*	Rhizocarpales	NO, Buskerud, 2013, S. Rui & E. Timdal	O-L-184267	OLICH531-13	KT800003	no lichen substances
*Rhizocarpon rittokense*	Rhizocarpales	NO, Finnmark, 2014, M. Westberg	O-L-195695	OLICH1567-14	KU687458	
*Rhizocarpon rittokense*	Rhizocarpales	NO, Oppland, 2013, M. Bendiksby et al.	O-L-184293	OLICH542-13	KU687459	
*Rhizocarpon santessonii*	Rhizocarpales	NO, Oppland, 2011, R. Haugan & E. Timdal	O-L-169805	OLICH432-13	KU687454	
*Rhizocarpon subgeminatum*	Rhizocarpales	NO, Hedmark, 2010, R. Haugan	O-L-166501	OLICH033-11	KU687457	barbatic acid
*Rhizocarpon subgeminatum*	Rhizocarpales	NO, Sør-Trøndelag, 2010, R. Haugan	O-L-165477	OLICH034-11	KU687452	barbatic acid
*Rhizocarpon suomiense*	Rhizocarpales	NO, Oppland, 2013, M. Bendiksby et al.	O-L-184330	OLICH769-13	KU687448	
*Rhizoplaca melanophthalma*	Lecanorales	NO, Finnmark, 2014, E. Timdal	O-L-195929	OLICH1774-14	MK812478	unknown substances, usnic acid
*Rhizoplaca melanophthalma*	Lecanorales	NO, Oppland, 2015, E. Timdal	O-L-201366	OLICH2743-16	MK811669	
*Rinodina balanina*	Caliciales	NO, Finnmark, 2014, T.B. Wheeler	O-L-195705	OLICH1577-14	KY266842	
*Rinodina disjuncta*	Caliciales	NO, Nord-Trøndelag, 2014, H. Holien	TRH-L-15387	OLICH3675-17	MK812529	
*Rinodina disjuncta*	Caliciales	NO, Nord-Trøndelag, 2011, H. Holien	TRH-L-14116	OLICH3676-17	MK812438	
*Rinodina mniaraea*	Caliciales	NO, Svalbard, 2001, A. Elvebakk	TROM_L_565871	OLICH3552-17	MK812098	
*Rinodina roboris*	Caliciales	NO, Rogaland, 2016, J.T. Klepsland	O-L-206765	OLICH3484-17	MK811851	
*Romjularia lurida*	Lecideales	NO, Akershus, 2010, E. Timdal	O-L-169118	OLICH150-11	MK811769	
*Ropalospora lugubris*	Umbilicariales	NO, Finnmark, 2014, E. Timdal	O-L-195537	OLICH1511-14	KY266893	
*Ropalospora lugubris*	Umbilicariales	NO, Nordland, 2015, T. Tønsberg & A. Botnen	BG-L-98792	OLICH3732-17	MK812617	
*Ropalospora lugubris*	Umbilicariales	NO, Finnmark, 2011, E. Timdal	O-L-170519	OLICH3969-17	MK812431	
*Ropalospora lugubris*	Umbilicariales	NO, Sør-Trøndelag, 2011, R. Haugan	O-L-168439	OLICH3970-17	MK812670	
*Ropalospora lugubris*	Umbilicariales	NO, Sogn og Fjordane, 2011, R. Haugan	O-L-173455	OLICH3971-17	MK812610	
*Rusavskia elegans*	Teloschistales	NO, Sør-Trøndelag, 2014, E. Timdal	O-L-196351	OLICH1910-15	MK812165	
*Rusavskia elegans*	Teloschistales	NO, Oppland, 2013, J.T. Klepsland	O-L-200120	OLICH2630-16	MK812212	
*Rusavskia sorediata*	Teloschistales	NO, Sør-Trøndelag, 2014, E. Timdal	O-L-196353	OLICH1912-15	MK811744	
*Sagiolechia protuberans*	Ostropales	NO, Finnmark, 2014, M. Westberg	O-L-195714	OLICH1586-14	KY266966	
*Sagiolechia protuberans*	Ostropales	NO, Nord-Trøndelag, 2015, R. Haugan	O-L-201297	OLICH3853-17	MK811691	
*Sclerophora peronella*	Coniocybales	SE, Södermanland, 2015, S. Kistenich & E. Timdal	O-L-200846	OLICH2432-16	MK812193	
*Scoliciosporum umbrinum*	Lecanorales	NO, Oslo, 2014, J.T. Klepsland	O-L-200123	OLICH3322-16	MK812255	
*Scoliciosporum umbrinum*	Lecanorales	NO, Akershus, 2012, E. Timdal	O-L-177770	OLICH822-13	MK812271	
*Siphula ceratites*	Pertusariales	NO, Finnmark, 2014, E. Timdal	O-L-195919	OLICH1764-14	KY266949	
*Siphula ceratites*	Pertusariales	NO, Nord-Trøndelag, 2015, E. Timdal et al.	O-L-201291	OLICH2679-16	MK812106	
*Siphula ceratites*	Pertusariales	NO, Sør-Trøndelag, 2014, R. Haugan	O-L-196042	OLICH3790-17	MK812034	
*Solorina saccata*	Peltigerales	NO, Buskerud, 2014, E. Timdal	O-L-203070	OLICH3286-16	MK812538	
*Sphaerophorus fragilis*	Lecanorales	NO, Troms, 2014, E. Timdal	O-L-195650	OLICH1562-14	MK812306	
*Sphaerophorus fragilis*	Lecanorales	NO, Hordaland, 2014, E. Timdal	O-L-195800	OLICH1672-14	MK811867	
*Sphaerophorus fragilis*	Lecanorales	NO, Finnmark, 2014, E. Timdal	O-L-195977	OLICH1822-14	KY266958	
*Sphaerophorus fragilis*	Lecanorales	NO, Sør-Trøndelag, 2014, R. Haugan	O-L-196043	OLICH3799-17	MK812393	
*Sphaerophorus globosus*	Lecanorales	NO, Troms, 2014, E. Timdal	O-L-195651	OLICH1563-14	MK811784	sphaerophorin
*Sphaerophorus globosus*	Lecanorales	NO, Finnmark, 2014, E. Timdal	O-L-195855	OLICH1700-14	KY266841	sphaerophorin
*Sphaerophorus globosus*	Lecanorales	NO, Finnmark, 2014, E. Timdal	O-L-195918	OLICH1763-14	KY266859	sphaerophorin, squamatic acid
*Sphaerophorus globosus*	Lecanorales	NO, Finnmark, 2014, H. Holien	O-L-195949	OLICH1794-14	KY266898	
*Sphaerophorus globosus*	Lecanorales	NO, Oslo, 2014, E. Timdal	O-L-199995	OLICH1969-15	MK812694	sphaerophorin, thamnolic acid, unknowns
*Sphaerophorus globosus*	Lecanorales	NO, Telemark, 2015, S. Rui & E. Timdal	O-L-200918	OLICH2578-16	MK812575	sphaerophorin
*Sphaerophorus globosus*	Lecanorales	NO, Sør-Trøndelag, 2014, R. Haugan	O-L-196029	OLICH3793-17	MK811761	sphaerophorin, squamatic acid
*Sphaerophorus globosus*	Lecanorales	NO, Nord-Trøndelag, 2015, R. Haugan	O-L-201265	OLICH3844-17	MK812327	sphaerophorin, thamnolic acid
*Staurolemma omphalarioides*	Peltigerales	NO, Nordland, 2013, J.T. Klepsland	O-L-197994	OLICH2467-16	MK811827	
*Staurolemma omphalarioides*	Peltigerales	NO, Nordland, 2014, J.T. Klepsland	O-L-200306	OLICH2640-16	MK811929	
*Stereocaulon alpinum*	Lecanorales	NO, Telemark, 2006, E. Timdal	O-L-149404	OLICH925-13	MK812253	atranorin, lobaric acid
*Stereocaulon capitellatum*	Lecanorales	NO, Oppland, 2015, E. Timdal	O-L-201401	OLICH4182-17	MK812320	anziaic acid, atranorin, miriquidic acid, perlatolic acid
*Stereocaulon coniophyllum*	Lecanorales	NO, Sogn og Fjordane, 2010, S. Vatne	O-L-169267	OLICH713-13	MK811930	lobaric acid, atranorin
*Stereocaulon cumulatum*	Lecanorales	NO, Finnmark, 2014, M. Svensson	O-L-195924	OLICH1769-14	KY266904	
*Stereocaulon cumulatum*	Lecanorales	NO, Buskerud, 2015, S. Rui & E. Timdal	O-L-200899	OLICH2529-16	MK812171	
*Stereocaulon cumulatum*	Lecanorales	NO, Hedmark, 2013, E. Timdal	O-L-184515	OLICH674-13	MK812474	
*Stereocaulon cumulatum*	Lecanorales	NO, Oppland, 2012, E. Timdal	O-L-182037	OLICH711-13	MK812671	
*Stereocaulon grande*	Lecanorales	NO, Troms, 2003, E. Timdal	O-L-122991	OLICH917-13	MK812157	atranorin, bourgeanic acid, lobaric acid
*Stereocaulon pileatum*	Lecanorales	NO, Aust-Agder, 2016, S. Rui & E. Timdal	O-L-204980	OLICH4012-17	MK812053	
*Stereocaulon pileatum*	Lecanorales	NO, Østfold, 2002, B.P. Løfall & A. Often	O-L-113318	OLICH908-13	MK812510	atranorin, lobaric acid
*Stereocaulon pileatum*	Lecanorales	NO, Oslo, 2011, H. Bratli & R. Haugan	O-L-174391	OLICH909-13	MK811864	atranorin, lobaric acid
*Stereocaulon rivulorum*	Lecanorales	NO, Troms, 2003, E. Timdal	O-L-127342	OLICH899-13	MK812658	anziaic acid, atranorin, perlatolic acid
*Stereocaulon rivulorum*	Lecanorales	NO, Finnmark, 2003, E. Timdal	O-L-123189	OLICH901-13	MK811662	anziaic acid, atranorin, perlatolic acid
*Stereocaulon symphycheilum*	Lecanorales	NO, Oppland, 2012, H. Bratli	O-L-183927	OLICH706-13	MK811941	lobaric acid
*Stereocaulon symphycheilum*	Lecanorales	NO, Buskerud, 2012, S. Rui & E. Timdal	O-L-184640	OLICH730-13	MK812328	atranorin, lobaric acid
*Stereocaulon symphycheilum*	Lecanorales	NO, Sør-Trøndelag, 2009, E. Timdal	O-L-158545	OLICH895-13	MK812189	atranorin, lobaric acid
*Sticta fuliginoides*	Peltigerales	NO, Rogaland, 2012, B. Nordén & J.B. Jordal	O-L-199414	OLICH2638-16	MK811703	
*Sticta sylvatica*	Peltigerales	NO, Telemark, 2015, S. Rui & E. Timdal	O-L-200925	OLICH2539-16	MK811909	
*Tephromela atra*	Lecanorales	NO, Finnmark, 2014, A. Millanes	O-L-195861	OLICH1706-14	KY266944	
*Tephromela atra*	Lecanorales	NO, Sør-Trøndelag, 2014, R. Haugan	O-L-196054	OLICH3815-17	MK812606	
*Tephromela atra*	Lecanorales	NO, Telemark, 2009, R. Haugan & S. Reiso	O-L-160434	OLICH399-13	MK812380	
*Tephromela grumosa*	Lecanorales	NO, Oppland, 2013, M. Bendiksby et al.	O-L-190787	OLICH2594-16	MK812127	atranorin, lichesterinic acid
*Tetramelas chloroleucus*	Caliciales	NO, Sør-Trøndelag, 2014, H. Holien	TRH-L-15560	OLICH3601-17	MK811644	
*Tetramelas chloroleucus*	Caliciales	NO, Nordland, 2013, J.T. Klepsland	O-L-198004	OLICH4032-17	MK812300	
*Thamnolia vermicularis*	Pertusariales	NO, Finnmark, 2014, E. Timdal	O-L-195710	OLICH1582-14	KY266979	
*Thamnolia vermicularis*	Pertusariales	NO, Troms, 2014, E. Timdal	O-L-195773	OLICH1645-14	MK812339	
*Thamnolia vermicularis*	Pertusariales	NO, Finnmark, 2014, H. Holien	O-L-195948	OLICH1793-14	KY266969	
*Thamnolia vermicularis*	Pertusariales	NO, Sør-Trøndelag, 2014, E. Timdal	O-L-196363	OLICH1922-15	MK811783	
*Thamnolia vermicularis*	Pertusariales	NO, Buskerud, 2015, E. Timdal & S. Rui	O-L-200905	OLICH3840-17	MK811627	
*Thelotrema lepadinum*	Ostropales	NO, Hordaland, 2013, B. Nordén & J.B. Jordal	O-L-199496	OLICH3321-16	MK812434	
*Thelotrema macrosporum*	Ostropales	NO, Hordaland, 2013, B. Nordén & J.B. Jordal	O-L-199493	OLICH3320-16	MK812111	
*Tholurna dissimilis*	Caliciales	NO, Buskerud, 2014, E. Timdal	O-L-195822	OLICH2194-15	MK812234	
*Tholurna dissimilis*	Caliciales	NO, Nord-Trøndelag, 2015, H. Holien	TRH-L-16038	OLICH2760-16	MK811683	
*Timdalia intricata*	Acarosporales	NO, Telemark, 2013, E. Timdal	O-L-194731	OLICH3244-16	MK812713	
*Timdalia intricata*	Acarosporales	NO, Oppland, 2015, E. Timdal	O-L-201379	OLICH4168-17	MK812246	
*Timdalia intricata*	Acarosporales	NO, Hedmark, 2017, E. Timdal	O-L-207764	OLICH4349-17	MK811937	psoromic acid
*Timdalia intricata*	Acarosporales	SE, Jämtland, 2017, E. Timdal	O-L-208174	OLICH4350-17	MK811871	psoromic acid
*Timdalia intricata*	Acarosporales	SE, Härjedalen, 2017, E. Timdal	O-L-208190	OLICH4351-17	MK812454	psoromic acid
*Toensbergia leucococca*	Rhizocarpales	NO, Nordland, 2012, T. Tønsberg	BG-L-94650	OLICH592-13	MK812615	
*Toninia alutacea*	Lecanorales	NO, Finnmark, 2014, E. Timdal	O-L-195931	OLICH1776-14	MG838194	
*Toninia alutacea*	Lecanorales	NO, Buskerud, 2014, J.T. Klepsland	O-L-200591	OLICH2636-16	MG838164	
*Toninia alutacea*	Lecanorales	NO, Oppland, 2013, M. Bendiksby et al.	O-L-184350	OLICH551-13	MG838166	
*Toninia alutacea*	Lecanorales	NO, Troms, 2011, J.T. Klepsland	O-L-177188	OLICH561-13	MG838197	
*Toninia alutacea*	Lecanorales	NO, Oppland, 2011, J.T. Klepsland	O-L-183755	OLICH783-13	MG838156	
*Toninia aromatica*	Lecanorales	NO, Sør-Trøndelag, 2014, J.T. Klepsland	O-L-200557	OLICH3317-16	MG838189	
*Toninia aromatica*	Lecanorales	NO, Troms, 2009, J.T. Klepsland	O-L-167368	OLICH560-13	MG838184	
*Toninia aromatica*	Lecanorales	NO, Nordland, 2011, R. Haugan	O-L-174042	OLICH614-13	MG838161	
*Toninia candida*	Lecanorales	NO, Buskerud, 2014, E. Timdal	O-L-196069	OLICH1885-14	MG838163	
*Toninia candida*	Lecanorales	NO, Buskerud, 2011, K.A. Lye & T. Høitomt	O-L-177883	OLICH557-13	MG838185	
*Toninia coelestina*	Lecanorales	NO, Nordland, 2010, J.T. Klepsland	O-L-175215	OLICH285-13	MG838190	
*Toninia nordlandica*	Lecanorales	NO, Finnmark, 2014, E. Timdal	O-L-195601	OLICH1537-14	MG838198	
*Toninia nordlandica*	Lecanorales	NO, Oppland, 2013, M. Bendiksby et al.	O-L-184351	OLICH552-13	MG838159	
*Toninia opuntioides*	Lecanorales	NO, Finnmark, 2014, E. Timdal	O-L-195603	OLICH1539-14	MG838192	
*Toninia opuntioides*	Lecanorales	NO, Oppland, 2011, J.T. Klepsland	O-L-183630	OLICH559-13	MG838168	triterpenoid (minor)
*Toninia opuntioides*	Lecanorales	NO, Oppland, 2013, M. Bendiksby et al.	O-L-184387	OLICH635-13	MG838172	triterpenoid (possible trace)
*Toninia opuntioides*	Lecanorales	NO, Oppland, 2011, J.T. Klepsland	O-L-183757	OLICH784-13	MG838158	
*Toninia rosulata*	Lecanorales	NO, Nord-Trøndelag, 2011, J.T. Klepsland	O-L-177141	OLICH1323-14	MG838195	
*Toninia sedifolia*	Lecanorales	NO, Finnmark, 2014, E. Timdal	O-L-195599	OLICH1536-14	MG838193	
*Toninia sedifolia*	Lecanorales	NO, Aust-Agder, 2010, J.T. Klepsland	O-L-183585	OLICH558-13	MG838201	no lichen substances
*Toninia sedifolia*	Lecanorales	NO, Oppland, 2013, M. Bendiksby et al.	O-L-184381	OLICH637-13	MG838196	
*Toninia sedifolia*	Lecanorales	NO, Oppland, 2013, M. Bendiksby et al.	O-L-184332	OLICH817-13	MG838162	
*Toninia squalida*	Lecanorales	NO, Oppland, 2011, R. Haugan & E. Timdal	O-L-169774	OLICH553-13	MG838183	
*Toninia squalida*	Lecanorales	NO, Oppland, 2012, H. Bratli & R. Haugan	O-L-182301	OLICH554-13	MG838182	
*Toninia squalida*	Lecanorales	NO, Hordaland, 2013, E. Timdal	O-L-184426	OLICH638-13	MG838181	
*Toninia squalida*	Lecanorales	NO, Sør-Trøndelag, 2013, E. Timdal	O-L-184539	OLICH639-13	MG838200	
*Toninia taurica*	Lecanorales	NO, Oppland, 2013, M. Bendiksby et al.	O-L-184333	OLICH546-13	MG838174	
*Toninia verrucarioides*	Lecanorales	NO, Oppland, 2013, M. Bendiksby et al.	O-L-184389	OLICH641-13	MG838167	
*Toninia verrucarioides*	Lecanorales	NO, Oppland, 2011, J.T. Klepsland	O-L-183717	OLICH850-13	MG838179	
*Trapelia coarctata*	Trapeliales	NO, Oslo, 2014, J.T. Klepsland	O-L-200174	OLICH3316-16	MK812584	
*Trapelia coarctata*	Trapeliales	NO, Hedmark, 2012, R. Haugan et al.	O-L-182063	OLICH562-13	MK812526	
*Trapelia coarctata*	Trapeliales	NO, Telemark, 2012, E. Timdal	O-L-179924	OLICH563-13	MK812177	
*Trapelia corticola*	Trapeliales	NO, Nord-Trøndelag, 2011, H. Holien	TRH-L-14083	OLICH993-13	MK811911	
*Trapelia placodioides*	Trapeliales	NO, Nord-Trøndelag, 2012, R. Haugan	O-L-183857	OLICH1324-14	MK812664	
*Trapelia placodioides*	Trapeliales	NO, Telemark, 1996, H. Bratli & E. Timdal	O-L-20869	OLICH3976-17	MK812430	
*Trapelia placodioides*	Trapeliales	NO, Hedmark, 2015, R. Haugan	O-L-204154	OLICH4366-17	MK812690	gyrophoric acid, lecanoric acid
*Trapelia placodioides*	Trapeliales	NO, Oslo, 2014, J.T. Klepsland	O-L-200245	OLICH4453-17	MK812050	
*Trapelia placodioides*	Trapeliales	NO, Oppland, 2013, M. Bendiksby et al.	O-L-184337	OLICH548-13	MK812359	
*Trapelia placodioides*	Trapeliales	NO, Oppland, 2012, S. Rui & E. Timdal	O-L-179918	OLICH569-13	MK812043	
*Trapeliopsis flexuosa*	Trapeliales	NO, Nord-Trøndelag, 2013, H. Holien	TRH-L-15151	OLICH994-13	MK811801	
*Trapeliopsis granulosa*	Trapeliales	NO, Sør-Trøndelag, 2007, E. Timdal	O-L-158481	OLICH565-13	MK812609	
*Trapeliopsis granulosa*	Trapeliales	NO, Oslo, 2011, R. Haugan	O-L-177342	OLICH566-13	MK811943	
*Trapeliopsis granulosa*	Trapeliales	NO, Troms, 2011, J.T. Klepsland	O-L-177175	OLICH567-13	MK812708	
*Trapeliopsis granulosa*	Trapeliales	NO, Finnmark, 2011, E. Timdal	O-L-170698	OLICH568-13	MK812198	
*Trapeliopsis granulosa*	Trapeliales	NO, Nord-Trøndelag, 2013, H. Holien	TRH-L-15150	OLICH995-13	MK812377	
*Trapeliopsis pseudogranulosa*	Trapeliales	NO, Telemark, 2015, E. Timdal	O-L-208001	OLICH3875-17	MK812568	
*Trapeliopsis pseudogranulosa*	Trapeliales	NO, Rogaland, 2011, J.T. Klepsland	O-L-183692	OLICH852-13	MK812270	
*Tremolecia atrata*	Hymeneliales	NO, Sør-Trøndelag, 2012, M. Bendiksby et al.	O-L-179677	OLICH4017-17	MK812298	
*Tremolecia atrata*	Hymeneliales	NO, Finnmark, 2011, E. Timdal	O-L-170578	OLICH606-13	MK811647	
*Tremolecia atrata*	Hymeneliales	NO, Finnmark, 2011, E. Timdal	O-L-170776	OLICH607-13	MK812097	
*Tuckermanopsis chlorophylla*	Lecanorales	NO, Sør-Trøndelag, 2014, R. Haugan	O-L-196001	OLICH1846-14	MK811914	
*Tuckermanopsis chlorophylla*	Lecanorales	NO, Akershus, 2014, S. Rui & E. Timdal	O-L-196329	OLICH2260-15	MK812387	
*Umbilicaria arctica*	Umbilicariales	NO, Finnmark, 2014, E. Timdal	O-L-195898	OLICH1743-14	KY266911	
*Umbilicaria arctica*	Umbilicariales	NO, Buskerud, 2015, S. Rui & E. Timdal	O-L-200892	OLICH2545-16	MK812428	
*Umbilicaria arctica*	Umbilicariales	NO, Aust-Agder, 2016, S. Rui & E. Timdal	O-L-204983	OLICH4016-17	MK812268	
*Umbilicaria cinereorufescens*	Umbilicariales	NO, Buskerud, 2014, E. Timdal	O-L-195830	OLICH2202-15	MK811655	
*Umbilicaria cinereorufescens*	Umbilicariales	NO, Oppland, 2015, E. Timdal	O-L-201349	OLICH2734-16	MK812545	
*Umbilicaria crustulosa*	Umbilicariales	NO, Hordaland, 2014, E. Timdal	O-L-195804	OLICH1676-14	MK811776	
*Umbilicaria crustulosa*	Umbilicariales	NO, Buskerud, 2015, S. Rui & E. Timdal	O-L-200941	OLICH2662-16	MK812060	
*Umbilicaria cylindrica*	Umbilicariales	NO, Hordaland, 2014, E. Timdal	O-L-195808	OLICH1680-14	MK811748	
*Umbilicaria cylindrica*	Umbilicariales	NO, Finnmark, 2014, T. Tønsberg	O-L-195872	OLICH1717-14	KY266907	
*Umbilicaria cylindrica*	Umbilicariales	NO, Finnmark, 2014, H. Holien	O-L-195957	OLICH1802-14	KY266835	
*Umbilicaria cylindrica*	Umbilicariales	NO, Sør-Trøndelag, 2014, E. Timdal	O-L-196354	OLICH1913-15	MK812072	
*Umbilicaria cylindrica*	Umbilicariales	NO, Buskerud, 2013, S. Rui & E. Timdal	O-L-184711	OLICH929-13	MK812153	
*Umbilicaria decussata*	Umbilicariales	NO, Sør-Trøndelag, 2015, M. Stapnes Dahl et al.	O-L-201307	OLICH2692-16	MK812293	
*Umbilicaria decussata*	Umbilicariales	NO, Oppland, 2015, E. Timdal	O-L-201367	OLICH2744-16	MK812686	
*Umbilicaria dendrophora*	Umbilicariales	NO, Sør-Trøndelag, 2013, E. Timdal	O-L-184529	OLICH731-13	MK812276	
*Umbilicaria deusta*	Umbilicariales	NO, Finnmark, 2014, E. Timdal	O-L-195766	OLICH1638-14	KY266862	
*Umbilicaria deusta*	Umbilicariales	NO, Telemark, 2014, E. Timdal	O-L-195790	OLICH1662-14	MK812011	
*Umbilicaria deusta*	Umbilicariales	NO, Vestfold, 2015, E. Timdal	O-L-198940	OLICH2493-16	MK812096	
*Umbilicaria deusta*	Umbilicariales	NO, Buskerud, 2013, S. Rui & E. Timdal	O-L-184712	OLICH930-13	MK812412	
*Umbilicaria havaasii*	Umbilicariales	NO, Telemark, 2015, S. Rui & E. Timdal	O-L-200930	OLICH2556-16	MK812376	
*Umbilicaria hirsuta*	Umbilicariales	NO, Vestfold, 2014, S. Rui & E. Timdal	O-L-196282	OLICH2212-15	MK812437	
*Umbilicaria hirsuta*	Umbilicariales	NO, Vestfold, 2015, E. Timdal	O-L-198937	OLICH2490-16	MK811754	
*Umbilicaria hirsuta*	Umbilicariales	NO, Oppland, 2015, E. Timdal	O-L-201350	OLICH2735-16	MK812136	
*Umbilicaria hyperborea*	Umbilicariales	NO, Hordaland, 2014, E. Timdal	O-L-195803	OLICH1675-14	MK812311	
*Umbilicaria hyperborea*	Umbilicariales	NO, Finnmark, 2014, R. Haugan	O-L-195879	OLICH1724-14	KY266918	
*Umbilicaria hyperborea*	Umbilicariales	NO, Finnmark, 2014, R. Haugan	O-L-195925	OLICH1770-14	KY266976	
*Umbilicaria hyperborea*	Umbilicariales	NO, Finnmark, 2014, E. Timdal	O-L-195971	OLICH1816-14	KY266928	
*Umbilicaria hyperborea*	Umbilicariales	NO, Troms, 2014, E. Timdal	O-L-195656	OLICH1996-15	MK812016	
*Umbilicaria hyperborea*	Umbilicariales	NO, Buskerud, 2013, S. Rui & E. Timdal	O-L-184713	OLICH931-13	MK811931	
*Umbilicaria lyngei*	Umbilicariales	NO, Sør-Trøndelag, 2000, B. P. Løfall	O-L-78093	OLICH4074-17	MK812368	no lichen substances
*Umbilicaria lyngei*	Umbilicariales	NO, Oppland, 2009, R. Haugan	O-L-159920	OLICH4075-17	MK812559	
*Umbilicaria lyngei*	Umbilicariales	NO, Sør-Trøndelag, 2014, J.T. Klepsland	O-L-200558b	OLICH4096-17	MK811698	
*Umbilicaria nylanderiana*	Umbilicariales	NO, Hedmark, 2015, M. Stapnes Dahl et al.	O-L-201305	OLICH2670-16	MK812167	
*Umbilicaria nylanderiana*	Umbilicariales	NO, Hedmark, 2016, E. Timdal	O-L-201508	OLICH3983-17	MK812209	
*Umbilicaria nylanderiana*	Umbilicariales	NO, Buskerud, 2013, S. Rui & E. Timdal	O-L-184729	OLICH950-13	MK812316	
*Umbilicaria polyrrhiza*	Umbilicariales	NO, Oslo, 2014, E. Timdal	O-L-199999	OLICH1973-15	MK812126	
*Umbilicaria polyrrhiza*	Umbilicariales	NO, Østfold, 2013, R. Braathen & E.W. Hanssen	O-L-201768	OLICH2595-16	MK812352	
*Umbilicaria polyrrhiza*	Umbilicariales	NO, Østfold, 2017, S. Rui & E. Timdal	O-L-208345	OLICH4384-17	MK812239	
*Umbilicaria proboscidea*	Umbilicariales	NO, Finnmark, 2014, H. Holien	O-L-195719	OLICH1591-14	KY266839	
*Umbilicaria proboscidea*	Umbilicariales	NO, Troms, 2014, E. Timdal	O-L-195772	OLICH1644-14	MK811704	
*Umbilicaria proboscidea*	Umbilicariales	NO, Finnmark, 2014, R. Haugan	O-L-195923	OLICH1768-14	KY266848	
*Umbilicaria proboscidea*	Umbilicariales	NO, Finnmark, 2014, A. Millanes	O-L-195936	OLICH1781-14	KY266866	
*Umbilicaria proboscidea*	Umbilicariales	NO, Sør-Trøndelag, 2014, E. Timdal	O-L-196361	OLICH1920-15	MK812366	
*Umbilicaria proboscidea*	Umbilicariales	NO, Buskerud, 2014, S. Rui & E. Timdal	O-L-196322	OLICH2252-15	MK812345	
*Umbilicaria rigida*	Umbilicariales	SE, Torne Lappmark, 2014, E. Timdal	O-L-195668	OLICH2005-15	MK812082	
*Umbilicaria rigida*	Umbilicariales	NO, Møre og Romsdal, 2015, R. Haugan	O-L-203950	OLICH4077-17	MK811777	
*Umbilicaria spodochroa*	Umbilicariales	NO, Vestfold, 2015, E. Timdal	O-L-198938	OLICH2491-16	MK812519	
*Umbilicaria torrefacta*	Umbilicariales	NO, Troms, 2014, E. Timdal	O-L-195634	OLICH1553-14	MK812116	
*Umbilicaria torrefacta*	Umbilicariales	NO, Finnmark, 2014, E. Timdal	O-L-195843	OLICH1688-14	KY266942	
*Umbilicaria torrefacta*	Umbilicariales	NO, Telemark, 2015, S. Rui & E. Timdal	O-L-200900	OLICH2530-16	MK812602	
*Umbilicaria vellea*	Umbilicariales	NO, Telemark, 2014, E. Timdal	O-L-196275	OLICH1949-15	MK811710	
*Umbilicaria virginis*	Umbilicariales	NO, Møre og Romsdal, 2015, R. Haugan	O-L-203963	OLICH3162-16	MK812576	
*Usnea dasypoga*	Lecanorales	NO, Buskerud, 2013, S. Rui & E. Timdal	O-L-184699	OLICH1411-14	MK812140	
*Usnea dasypoga*	Lecanorales	NO, Telemark, 2014, E. Timdal	O-L-196273	OLICH1947-15	MK812173	
*Usnea dasypoga*	Lecanorales	NO, Oslo, 2014, E. Timdal	O-L-200005	OLICH1979-15	MK812052	
*Usnea flammea*	Lecanorales	NO, Vest-Agder, 2013, J.T. Klepsland	O-L-197827	OLICH2127-15	MK811847	constictic acid, stictic acid, usnic acid
*Usnea fragilescens*	Lecanorales	NO, Rogaland, 2014, J.T. Klepsland	O-L-200604	OLICH2634-16	MK812021	
*Usnea subfloridana*	Lecanorales	NO, Buskerud, 2013, S. Rui & E. Timdal	O-L-184700	OLICH1412-14	MK811828	
*Usnea subfloridana*	Lecanorales	NO, Telemark, 2015, S. Rui & E. Timdal	O-L-200814	OLICH2585-16	MK811766	
*Usnea subfloridana*	Lecanorales	NO, Rogaland, 2014, J.T. Klepsland	O-L-200728	OLICH2623-16	MK812453	thamnolic acid, usnic acid
*Usnea wasmuthii*	Lecanorales	NO, Sør-Trøndelag, 2013, J.T. Klepsland	O-L-198061	OLICH2131-15	MK812232	barbatic acid, salazinic acid, usnic acid
*Usnea wasmuthii*	Lecanorales	NO, Rogaland, 2013, J.T. Klepsland	O-L-197890	OLICH2132-15	MK812501	barbatic acid, usnic acid
*Varicellaria lactea*	Pertusariales	NO, Oppland, 2015, R. Haugan	O-L-204064	OLICH4380-17	MK812539	lecanoric acid, variolaric acid
*Variospora flavescens*	Teloschistales	NO, Rogaland, 2014, J.T. Klepsland	O-L-200626	OLICH4054-17	MK812667	
*Variospora thallincola*	Teloschistales	NO, Rogaland, 2014, J.T. Klepsland	O-L-200631	OLICH4056-17	MK812547	
*Verrucaria tenebrosa*	Verrucariales	No, Nord-Trøndelag, 2015, J. Pykälä 48333	H-WG2_2130	OLICH2807-16	KY682961	
*Vulpicida juniperinus*	Lecanorales	NO, Oppland, 2013, M. Bendiksby et al.	O-L-184298	OLICH1406-14	MK812148	
*Vulpicida juniperinus*	Lecanorales	NO, Nord-Trøndelag, 2015, R. Haugan	O-L-201311	OLICH3857-17	MK812404	
*Vulpicida juniperinus*	Lecanorales	NO, Sør-Trøndelag, 2017, E. Timdal	O-L-208203	OLICH4356-17	MK812500	
*Vulpicida pinastri*	Lecanorales	NO, Finnmark, 2014, H. Holien	O-L-195944	OLICH1789-14	KY266954	
*Vulpicida pinastri*	Lecanorales	NO, Telemark, 2015, S. Rui & E. Timdal	O-L-200937	OLICH2563-16	MK811933	
*Vulpicida pinastri*	Lecanorales	NO, Buskerud, 2013, S. Rui & E. Timdal	O-L-184701	OLICH883-13	MK812364	pinastric acid, vulpinic acid
*Xanthomendoza borealis*	Teloschistales	NO, Sør-Trøndelag, 2014, E. Timdal	O-L-196336	OLICH2274-15	MK812442	
*Xanthomendoza borealis*	Teloschistales	NO, Oppland, 2013, J.T. Klepsland	O-L-198333	OLICH2359-15	MK811659	
*Xanthomendoza fulva*	Teloschistales	NO, Vestfold, 2014, S. Rui & E. Timdal	O-L-196295	OLICH2225-15	MK812265	
*Xanthomendoza fulva*	Teloschistales	NO, Hedmark, 2006, B. P. Løfall et al.	O-L-147714	OLICH4440-17	MK812487	
*Xanthomendoza fulva*	Teloschistales	NO, Sogn og Fjordane, 2012, B. Nordén & J.B. Jordal	O-L-199397	OLICH4442-17	MK811973	
*Xanthoparmelia conspersa*	Lecanorales	NO, Telemark, 2014, S. Rui & E. Timdal	O-L-196278	OLICH1951-15	MK812598	
*Xanthoparmelia conspersa*	Lecanorales	NO, Vestfold, 2014, S. Rui & E. Timdal	O-L-196296	OLICH2226-15	MK812585	
*Xanthoparmelia conspersa*	Lecanorales	NO, Rogaland, 2016, S. Rui & E. Timdal	O-L-204999	OLICH3992-17	MK812248	
*Xanthoparmelia loxodes*	Lecanorales	NO, Akershus, 2014, E. Timdal	O-L-199990	OLICH1964-15	MK811808	
*Xanthoparmelia loxodes*	Lecanorales	NO, Vestfold, 2014, S. Rui & E. Timdal	O-L-196281	OLICH2211-15	MK811902	
*Xanthoparmelia loxodes*	Lecanorales	NO, Aust-Agder, 2013, J.T. Klepsland	O-L-198317	OLICH2375-16	MK811982	
*Xanthoparmelia loxodes*	Lecanorales	NO, Østfold, 2012, K.A. Lye	O-L-206876	OLICH4133-17	MK812427	
*Xanthoparmelia loxodes*	Lecanorales	NO, Østfold, 2016, S. Rui & E. Timdal	O-L-208018	OLICH4190-17	MK812543	
*Xanthoparmelia loxodes*	Lecanorales	NO, Østfold, 2017, S. Rui & E. Timdal	O-L-208343	OLICH4272-17	MK812627	
*Xanthoparmelia protomatrae*	Lecanorales	NO, Aust-Agder, 2015, H. Riddervold	O-L-201574	OLICH3243-16	MK812594	
*Xanthoparmelia pulla*	Lecanorales	NO, Sør-Trøndelag, 2014, R. Haugan	O-L-196012	OLICH1857-14	MK812429	
*Xanthoparmelia pulla*	Lecanorales	NO, Østfold, 2016, S. Rui & E. Timdal	O-L-208019	OLICH4191-17	MK812706	
*Xanthoparmelia pulla*	Lecanorales	NO, Østfold, 2017, S. Rui & E. Timdal	O-L-208344	OLICH4273-17	MK812285	
*Xanthoparmelia stenophylla*	Lecanorales	NO, Østfold, 2017, S. Rui & E. Timdal	O-L-208338	OLICH4268-17	MK811834	
*Xanthoparmelia tinctina*	Lecanorales	SE, Bohuslän, 2012, E. Timdal	O-L-184585	OLICH3233-16	MK812507	
*Xanthoparmelia tinctina*	Lecanorales	NO, Østfold, 2017, S. Rui & E. Timdal	O-L-208333	OLICH4264-17	MK812642	
*Xanthoparmelia verruculifera*	Lecanorales	NO, Oppland, 2015, E. Timdal	O-L-201346	OLICH2731-16	MK812281	
*Xanthoria parietina*	Teloschistales	NO, Telemark, 2014, E. Timdal	O-L-195782	OLICH1654-14	MK812349	
*Xanthoria parietina*	Teloschistales	NO, Oslo, 2014, M. Bendiksby et al.	O-L-196081	OLICH1897-14	MK812134	
*Xanthoria parietina*	Teloschistales	NO, Østfold, 2012, K.A. Lye	O-L-206852	OLICH4143-17	MK811702	
*Xylographa parallela*	Trapeliales	NO, Finnmark, 2011, E. Timdal	O-L-170700	OLICH3294-16	MK812221	no lichen substances
*Xylographa vitiligo*	Trapeliales	NO, Nord-Trøndelag, 2014, J.T. Klepsland	O-L-200273	OLICH3315-16	MK812010	

**Table 2.** Key statistics and genetic distances for the three genera with the most sampled species; *Ramalina*, *Umbilicaria* and *Cladonia*.

**Genus**	*** Ramalina ***	*** Umbilicaria ***	*** Cladonia ***
Number of species in genus in this dataset	9	19	47
Number of records	26	52	122
Average number of records per species	2.9	2.7	2.6
Average intraspecific distances	0–1.3%	0–2.3%	0–3.4%
Interspecific distances	0.4–8.2%	2.5–12.1%	0.2–15.6%
